# A convenient route to symmetrically and unsymmetrically substituted 3,5-diaryl-2,4,6-trimethylpyridines via Suzuki–Miyaura cross-coupling reaction

**DOI:** 10.3762/bjoc.12.82

**Published:** 2016-04-28

**Authors:** Dariusz Błachut, Joanna Szawkało, Zbigniew Czarnocki

**Affiliations:** 1 Forensic Laboratory, Internal Security Agency, 1 Sierpnia 30A, 02-134 Warsaw, Poland, Tel.: +48 693830760; 2 Faculty of Chemistry, University of Warsaw, Pasteura 1, 02-093 Warsaw, Poland

**Keywords:** arylpyridines, cross coupling reaction, heteroaromatics, one-pot reaction, palladium catalyst

## Abstract

A series of differently substituted 3,5-diaryl-2,4,6-trimethylpyridines were prepared and characterized using the Suzuki–Miyaura coupling reaction with accordingly selected bromo-derivatives and arylboronic acids. The reaction conditions were carefully optimized allowing high yield of isolated products and also the construction of unsymmetrically substituted diarylpyridines, difficult to access by other methods.

## Introduction

Nitrogen heterocycles are an important class of compounds widely present in agrochemical products [1–2] and pharmaceuticals [3–9]. Pyridine can be considered as one of the simplest yet popular members of this family. Differently substituted pyridine rings are present in several drugs, e.g., clarinex, a dual antagonist of platelet activating factor or in pheniramine and related pyridine-containing antihistamines for allergy treatment [10–13]. Other examples of pharmaceuticals containing a simple pyridine ring are rabeprazole [14–15], pentoprazole [16–17] and lansoprazole [18], which represent the well-known class of proton pump inhibitors. Furthermore, atropisomeric biaryls with a pyridine ring display promising properties in asymmetric catalysis [19–22].

**Figure 1:**
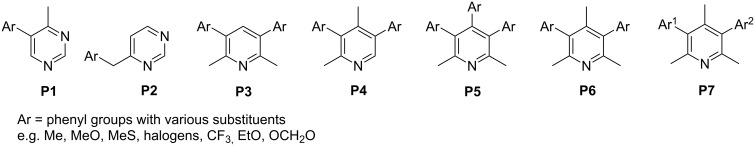
Types of aryl pyridines and pyrimidines already prepared in our group [23–27].

In our ongoing forensic chemistry programme directed toward the identification and synthesis of novel byproducts of illegally produced amphetamine analogues, we described the synthesis of several new nitrogen heterocycles. Among them methylarylpyrimidines **P1**, benzylpyrimidines **P2**, and aryl/methylpyridines **P3**, **P4** attracted our special attention (Figure 1) [23–27].

These compounds were identified as impurities in amphetamine or its analogues synthesized only by the Leuckart method starting from the corresponding 2-arylacetones. Therefore, the forensic community treats them as "route-specific impurities" or "route markers". The formation of these compounds results from the condensation of the drug precursors, like arylacetones with formamide or ammonia, in the presence of formic acid. This leads to di- and tetrahydrobenzyl/arylpyridines or pyrimidines with subsequent aromatization of the heterocyclic ring. We also noticed that the final composition of these markers was dependent on the reaction conditions during the drug synthesis [24].

In order to unequivocally confirm the presence of newly identified markers in the reaction mixture, we decided to develop a convenient method for their preparation on a multimilligram scale. In traditional synthetic methods, the substituted pyridine ring can be constructed, e.g., by Hantzsch reaction [28–29] or by condensation of amino-enone or aminonitrile derivatives with a 1,3-dicarbonyl system [30–31]. An important drawback of this strategy, especially when the access to a wide library of diversely decorated derivatives is necessary, is that the preparation requires an individually optimized synthetic procedure and the use of different substrates. Therefore, a more general strategy leading to a number of different 2,4- and 2,5-diaryldimethylpyridines **P3**, **P4** [25], 4-benzylpyrimidines **P2** [26], and 4-methyl-5-arylpyrimidines **P1** [26] was needed. For this purpose, we successfully used Suzuki and Negishi cross-coupling reactions between arylboronic acids/benzylzinc reagents and halogenated pyridines and pyrimidines.

During our search for new "route markers" of amphetamine analogues synthesized by the Leuckart method, we focused our attention on two groups of heterocycles, that were preliminary identified by GC–MS analysis as 3,4,5-triaryl-2,6-dimethylpyridines **P5** [32–33] and 3,5-diaryl-2,4,6-trimethylpyridines **P6**.

In this study we report on the synthesis of a series of 3,5-diaryl-2,4,6-trimethylpyridines **P6** (**4–29**) with substitution pattern at the phenyl ring as present in the most popular amphetamine-type drugs [23,27,34–36] (4-MeO, 4-MeS, 4-F, 4-Me, 3,4-OCH_2_O). We also decided to explore the possibility of the synthesis of diarylpyridines **P7** (**46–66**) with different aryl rings (Ar^1^ ≠ Ar^2^) as model compounds in a further study on the mechanism of their formation during amphetamine synthesis.

## Results and Discussion

### Symmetrically substituted 3,5-diaryl-2,4,6-trimethylpyridines

The starting material in the synthetic sequence, 3,5-dibromo-2,4,6-trimethylpyridine (**1**), can be easily prepared by simple bromination of 2,4,6-trimethylpyridine in 60% oleum on a multigram scale according to the procedure published by Drzeniek and Tomasikl [37].

In order to optimize the conditions for the preparation of diarylated pyridine **4**, the coupling of dibromopyridine **1** with phenylboronic acid was initially selected as a model reaction to study the effectiveness of various palladium sources, ligands, inorganic bases and solvent systems. Our goal was to maximize the yield of product **4** while suppressing the formation of the intermediate **3** and its des-brominated derivative **2** (Table 1). In a search for an efficient catalytic system, we took into consideration that dibromopyridine **1** having reaction sites at positions 3 and 5 of the pyridine ring, being shielded by methyl groups, might be less susceptible to the cross-coupling reaction. If the steric repulsion of the pyridine methyl group, at least in part, corresponds to its van der Waals radii, the degree of steric repulsion of the selected *ortho*-substituents in arylboronic acids can be listed in the following order: I > Br > Me > Cl > NO_2_ > CO_2_H > OMe > F > H [38–39]. The results of a series of the preliminary reactions are summarized in Table 1 and in Supporting Information File 1.

**Table 1:** Optimization of the reaction conditions.

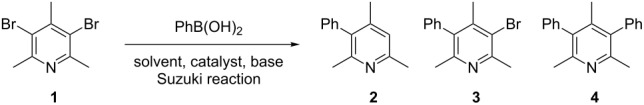								

Entry	Catalyst (mol %)	Base^a^	Solvent^b^	Time [h]	Conversion^c^	Yield [%]^d^		
						**2**	**3**	**4**

1	Pd(OAc)_2_ (10)	K_3_PO_4_	toluene/H_2_O	24	8	6	1	1
2	Pd(OAc)_2_ (10)	K_3_PO_4_	toluene/H_2_O	24	11	7	2	2
3	PdCl_2_ (10)	Na_2_CO_3_	toluene/H_2_O	24	10	6	3	1
4	Pd(OAc)_2_ (4), P(*o*-tol)_3_ (8)	Na_2_CO_3_	toluene/H_2_O/EtOH	0.25	85	4	51	30
5	Pd(OAc)_2_ (4), P(*o*-tol)_3_ (8)	Na_2_CO_3_	toluene/H_2_O/EtOH	0.5	94	3	49	42
6	Pd(OAc)_2_ (4), P(*o*-tol)_3_ (8)	Na_2_CO_3_	toluene/H_2_O/EtOH	1	~100	3	20	77
7	Pd(OAc)_2_ (4), P(*o*-tol)_3_ (8)	Na_2_CO_3_	toluene/H_2_O/EtOH	2	100	3	17	80
8	Pd(OAc)_2_ (4), P(*o*-tol)_3_ (8)	Na_2_CO_3_	toluene/H_2_O/EtOH	6	100	3	12	85
9	Pd(OAc)_2_ (4), P(*o*-tol)_3_ (8)	Na_2_CO_3_	toluene/H_2_O/EtOH	16	100	5	3	92
10	Pd(PPh_3_)_4_ (5)	Na_2_CO_3_	toluene/H_2_O/EtOH	16	100	traces	6	94
11	Pd(PPh_3_)_4_ (5)	NaOH	toluene/H_2_O/EtOH	16	76	75	–	~1
12	Pd(PPh_3_)_4_ (5)	Cs_2_CO_3_	toluene/H_2_O/EtOH	16	100	traces	23	77
13	Pd(PPh_3_)_4_ (5)	K_3_PO_4_	1,4-dioxane	16	100	5	15	80
14	Pd(PPh_3_)_4_ (5)	K_3_PO_4_	DMF	16	58	4	32	9
15	Pd(PPh_3_)_2_Cl_2_ (6)	Cs_2_CO_3_	toluene/H_2_O/EtOH	8	100	–	9	91
16	Pd(PPh_3_)_2_Cl_2_ (6)	K_3_PO_4_	toluene/H_2_O/EtOH	8	100	–	19	81
17	Pd(PPh_3_)_2_Cl_2_ (6)	K_3_PO_4_	toluene/H_2_O/EtOH	16	100	4	6	90
18	Pd(OAc)_2_ (4), P(Cy)_3_ (12)	Cs_2_CO_3_	toluene/H_2_O/EtOH	0.25	78	1	51	26
19	Pd(OAc)_2_ (4), P(Cy)_3_ (12)	Cs_2_CO_3_	toluene/H_2_O/EtOH	0.5	87	1	50	36
20	Pd(OAc)_2_ (4), P(Cy)_3_ (12)	Cs_2_CO_3_	toluene/H_2_O/EtOH	1	~99	2	11	86
21	Pd(OAc)_2_ (4), P(Cy)_3_ (12)	Cs_2_CO_3_	toluene/H_2_O/EtOH	2	100	2	–	98
22	Pd(OAc)_2_ (4), P(Cy)_3_ (12)	Cs_2_CO_3_	toluene/H_2_O/EtOH	8	100	1	–	99
23	Pd(OAc)_2_ (4), P(Cy)_3_ (12)	K_3_PO_4_	toluene/H_2_O/EtOH	3	100	1	–	99
24	Pd(OAc)_2_ (4), P(Cy)_3_ (12)	Na_2_CO_3_	toluene/H_2_O/EtOH	3	100	2	–	98
25	Pd(OAc)_2_ (4), P(Cy)_3_ (12)	K_2_CO_3_	toluene/H_2_O/EtOH	3	100	1	–	99
26	Pd(OAc)_2_ (4), P(Cy)_3_ (12)	K_3_PO_4_	1,4-dioxane	4	100	5	–	95
27	Pd(OAc)_2_ (4), P(Cy)_3_ (12)	CsF	toluene/H_2_O/EtOH	8	100	7	4	88
28	Pd(OAc)_2_ (4), P(Cy)_3_ (12)^e^	Cs_2_CO_3_	toluene/H_2_O/EtOH	16	100	2	–	98
29	Pd(dppf)Cl_2_ × CH_2_Cl_2_ (4)	CsF	1,4-dioxane	8	100	6	5	89
30	Pd(dppf)Cl_2_ × CH_2_Cl_2_ (4)	Cs_2_CO_3_	1,4-dioxane	4	100	3	–	97
31	Pd(dppf)Cl_2_ × CH_2_Cl_2_ (4)	K_3_PO_4_	1,4-dioxane	4	100	–	–	~99
32	Pd(dppf)Cl_2_ × CH_2_Cl_2_ (4)	K_3_PO_4_	DMF	6	100	1	2	97
33	Pd(dppf)Cl_2_ × CH_2_Cl_2_ (4)	KF	1,4-dioxane	16	100	10	2	88
34	Pd_2_(dba)_3_, (4) Symphos (8)	K_3_PO_4_	1,4-dioxane	8	64	14	19	31
35	Pd_2_(dba)_3_ (4), P(Cy)_3_ (12)	K_3_PO_4_	1,4-dioxane	8	100	5	–	95
36	Pd(OAc)_2_ (3), S-Phos (6)	K_3_PO_4_	toluene	1	100	–	–	~99
37	Pd(OAc)_2_ (3), X-Phos (6)	K_3_PO_4_	toluene	1	100	–	–	~99

^a^4 Equiv of base was used. ^b^Temperature of the reaction: toluene, 1,4-dioxane, DMF, toluene/H_2_O: 90 °C; solvent system toluene/H_2_O/EtOH: 85 °C. ^c^The conversion of substrate was measured by GC–MS. It was calculated as a percent ratio of unreacted **1** and the sum of the peak areas of **2**, **3** and **4**. ^d^The yield was estimated by GC–MS by comparison of peak area of particular product with the sum of areas of the rest of the products and unconverted substrate. ^e^Temperature of reaction 30 °C.

The ligand-free system based on palladium acetate (10 mol %) or palladium chloride (10 mol %) in the presence of K_3_PO_4_ and K_2_CO_3_ in a mixture of toluene/H_2_O (6:1) was initially tested. Recently, several ligand-free catalytic systems have been applied successfully in Suzuki cross-coupling reaction between various aryl and heteroaryl halides and triflates with alkenyl and arylboronic acids [40–43]. The evaluation of such catalysts, especially in water-based solvents, is important from the point of view of so-called “green chemistry” and also its economic efficiency [44–45]. In our hands, all attempts to carry out the reaction with both palladium acetate or palladium chloride gave the desired diarylated product **4** with unacceptable yield (1–2%) with low conversion of the precursor (8–11%). In each case, monoarylpyridine **2** was observed as the main product with trace amounts of its bromo derivative **3**. More drastic conditions (xylene, 130 °C, 48 h; not mentioned in Table 1) led only to fast debromination of the starting material **1**.

The screening was then continued with Pd(OAc)_2_ and tri-(*o*-tolyl)phosphine (P(*o*-tol)_3_) used as a catalyst in the mixture toluene/water/ethanol as solvent system and with sodium carbonate as a base. In order to control the progress of the process, the mixture was regularly sampled and the components ratio of was analysed by GC–MS (Table 1, entries 4–9). Full conversion of the starting material **1** was accomplished within 1 hour. However, the transformation of intermediary bromophenylpyridine **3** into **4** required further 15 hours. It was observed that during the last hour of the reaction, a significant acceleration of the dehaloganation process of **3** into monophenylpyridine **2** was observed. This phenomenon can be assigned to the stepwise slow decomposition of the catalytic complex into palladium metal. The effectiveness of commercially available Pd(PPh_3_)_4_ was verified in systems containing various bases and solvents (Table 1, entries 10–14). Full conversion of the substrate and highest yields of **4** were obtained when mild bases (Na_2_CO_3_, K_3_PO_4_, Cs_2_CO_3_) and toluene/EtOH/H_2_O and dioxane were applied (Table 1, entries 10, 12 and 13). However, the bromophenylpyridine **3** was still present in the reaction mixture. Dioxane and a solvent system based on toluene were more effective than DMF (Table 1, entry 14), for which the yield of the main product was only 9%. Surprisingly, the use of NaOH as a base led primarily to monoarylated product **2**. The comparison of these results with those obtained for catalyst Pd(P(*o*-tol)_3_)_4_ clearly confirms that the later compound is more active in the cross-coupling reaction [46].

The results of the cross coupling depend on electronic and steric properties of the ligands coordinated to the metal atom. In the case of phosphine-based ligands, the structure of the aryl and alkyl moiety is of crucial importance. As it was shown by Shen [47], Fu [48–49] and Monteith [50], the use of bulk and strongly electron-donating ligands (i.e., P(Cy)_3_ or P(*t*-Bu)_3_) accelerated the rate of the oxidative addition of aryl halides to the Pd-complex centre promoting the ligands dissociation leading to a more active monophosphine Pd complex. In our case, tricyclohexylphosphine associated with Pd(OAc)_2_ in toluene/H_2_O/EtOH confirmed its utility as a powerful catalytic system, giving fast and clean conversion of substrate **1** within 2–3 hours. When the reaction was performed with 2.8 equiv of phenylboronic acid in the presence of Pd(OAc)_2_/P(Cy)_3_, the final diarylpyridine **4** was obtained in each case in very good yield (95–99%, Table 1, entries 21–26) and the outcome of the reaction was independent on the base and the solvent used. The only exception was entry 27, where CsF as a mild base was applied. Fluorides are slightly less effective as bases in the case of other catalytic systems (Table 1, entries 29 and 33). It is worth to note that the increased activity of the catalyst based on tricyclohexylphosphine led us to perform the reaction at room temperature also in high yield (Table 1, entry 28). The screening was continued with PdCl_2_(dppf)×CH_2_Cl_2_ (4 mol %) in 1,4-dioxane and DMF in the presence of various bases. The results show that the catalyst is highly effective with combination of dioxane and K_3_PO_4_ as a base (Table 1, entry 31). According to Hayashi et al. [51–52], superior activity of dppf is due to favourable bite angle induced by the ligand in the catalytic complex.

As can be seen from Table 1, entries 34 and 35, the nature of the ligand affects the activity of the catalyst. The initially poor result obtained for Pd_2_(dba)_2_/Symphos (Table 1, entry 17) was greatly improved when P(Cy)_3_ was applied.

The best outcome in terms of the reaction time, yield of the diarylated product **4** and the content of byproducts were obtained using Pd(OAc)_2_ as palladium source and Buchwald ligands S-Phos and X-Phos [53–55] in toluene in the presence of K_3_PO_4_. As it was shown by GC–MS, full conversion of the substrate and almost quantitative formation of diarylated pyridine **4** was achieved within 1 hour.

**Scheme 1:**
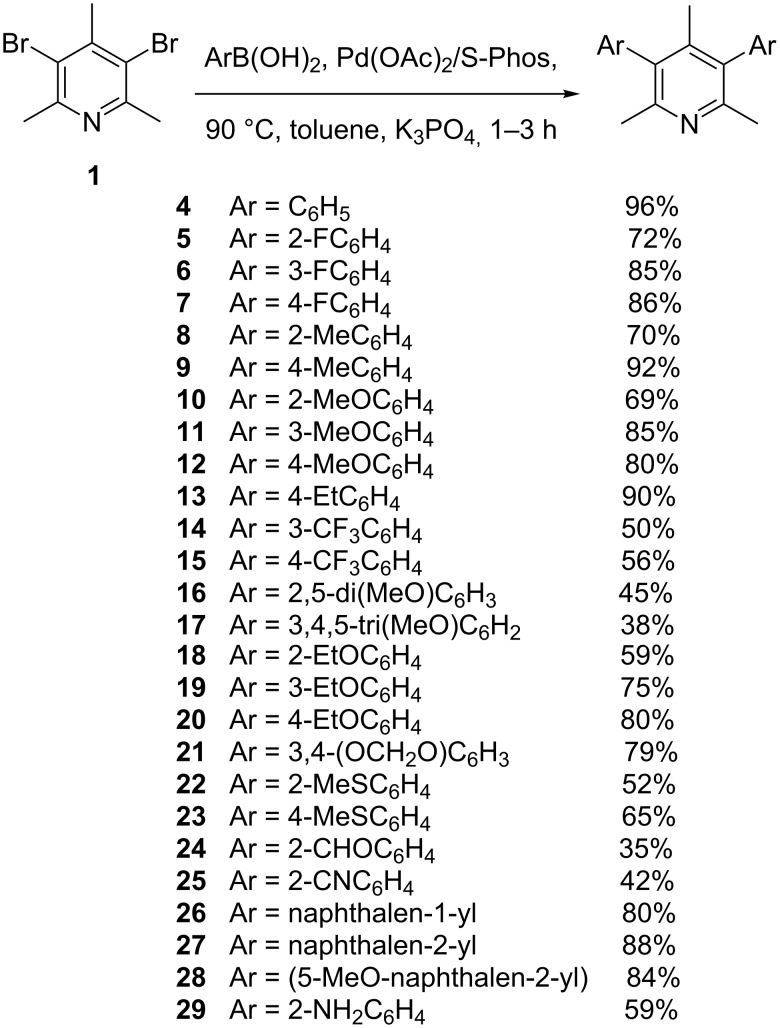
Synthesis of diarylpyridines **4–29**.

With the optimized conditions in hands, a library of 3,5-diaryl-2,4,6-trimethylpyridines **4–29** was prepared using variously substituted arylboronic acids **32–41** as cross-coupling partners (Scheme 1).

The best yields: 96%, 92% and 90% were obtained for phenylboronic acid and its 4-methyl and 4-ethyl-substituted derivatives, respectively. Arylboronic acids with electron-withdrawing and electron-donating groups attached at *meta* or *para* position of the phenyl ring afforded diarylpyridines in moderate to good yields (approx. 60–90%).

The arylboronic acids functionalized with strongly electron-withdrawing (3-CF_3_, 4-CF_3_, products **14** and **15**) and with more than one electron-donating groups (2,5-di-MeO, 3,4,5-tri-MeO, gave products **16** and **17**) with low yield and required further addition of reagents also with increased reaction time to complete the diarylation. The attachment of small substituents at *ortho* positions in arylboronic acids did not retard the reaction (products **5**, **8**, **10**, **18**, **22**, **24**, **25**, **29**). However, the GC–MS analysis revealed an increased formation of the corresponding biaryls, monoaryl- and bromoarypyridines, which difficult chromatographic separation also accounted for the low yield of the isolated final products. Attempted cross coupling of pyridine **1** with 4 mol excess of 2-chlorophenylboronic acids afforded a complex mixture of products with 3,5-bis(2-chlorophenyl)-2,4,6-trimethylpyridine in low yield (approx. 5%, not isolated), mainly because of the additional side-reactions that took place between 3-(2-chlorophenyl)-5-bromo-2,4,6-trimethylpyridine/diarylpyridine and 2-chlorophenylboronic acids at the C_phenyl_-Cl position, as was confirmed by GC–MS. The arylboronic acid with a valuable but unstable 2-formyl group gave again a low yield of product **24** (35%). Initially, our attempts to obtain pyridine **29** completely failed due to a very low solubility of 2-aminophenylboronic acid hydrochloride in toluene. The problem was overcome by addition of a small amount of water (approx 10 mol %) as a co-solvent with toluene. On the other hand, the attempted coupling of compound **41** with 2-nitrophenylboronic acid and (2-chloro-6-methoxyphenyl)boronic acid failed. Despite more drastic conditions (xylene, DMF, 145 °C, 48 h, 8 mol % Pd(OAc)_2_/16 mol % S-Phos) any amount of 3,5-bis(2-nitrophenyl)-2,4,6-trimethylpyridine and 3,5-bis(2-chloro-6-methoxyphenyl)-2,4,6-trimethylpyridine could be detected.

### Unsymmetrically substituted 3,5-diaryl-2,4,6-trimethylpyridines

In continuation of the study on the transformation of dibromopyridine **1** into diaryl derivatives, we focused our attention on de-symmetrization of the double cross coupling with the idea to obtain trimetylpyridine **P7** (**46–66**) decorated with different aryl rings. We envisaged that our goal could be achieved through sequential two-step reaction of **1** with the corresponding arylboronic acids. Such approach was widely applied by many research teams in the construction of polyarylated benzenes [56], pyridines [57–60], thiophenes [61–62], quinoxalines [63], pyrazoles [64] pyrroles [65], pyrimidines [66–67], benzofuranes [68], imidazo[1,2-*a*]pyridines [69], diaryl/heteroaryl methanes [70], and indoles [71], bearing differently substituted arene rings. An elegant approach to variously arylated pentaarylpyridines was recently proposed by Reimann et al. [60]. The final outcome of such a procedure is governed by many factors, including differences in site reactivity of polyhaloarenes (concerning both regio- and chemoselectivity), reaction conditions (the nature of the palladium/ligand, temperature, base, solvents) and the steric interactions between both cross-coupling partners. Minard et al. [59] pointed out that if an arylboronic acid involved in sequential couplings bears substituents with electron-withdrawing and electron-donating groups, the choice of the order of its introduction may be crucial for the final outcome. Our starting material constitutes a special case where both coupling sites are isoelectronic and equivalent in terms of steric hindrance. Such dihaloarenes, owing to the lack of electronic and steric differences between reaction sites, treated with 1 mol equivalent of boronic derivatives usually create a statistical distribution of products (1:2:1 for substrate:monaryl:diaryl). As can be found in many reports [72–73], the yield of the monoarylation reaction in similar cases does not exceed 50–60%. An exception is the reaction in which the first introduced aryl ring with halogen substituent increases the reactivity of the pyridine toward further diarylation activating the second reaction site, thus supporting the diarylation reaction.

**Scheme 2:**
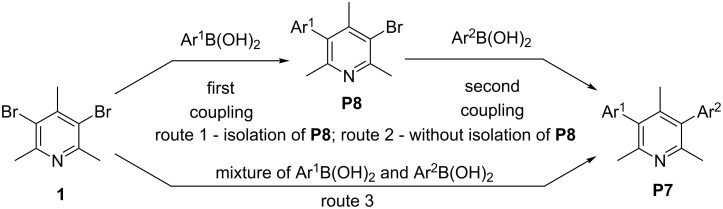
Synthetic routes leading to unsymmetrically substituted arylpyridines.

Three different approaches leading to unsymmetrically 3,5-diarylated trimethylpyridines **46–66** have been considered (Scheme 2, routes 1, 2, and 3, respectively). In the first one, we considered synthesizing the monoarylated intermediate **42–45** first, which after isolation and analytical characterization, would act as the substrate for the subsequent arylation (Scheme 2, route 1). This simple approach is tedious and time consuming; however the proper choice of conditions allows obtaining satisfactory yields of the unsymmetrically substituted diaryls in most cases.

The first step of such multistep processes requires fine tuning of the reaction conditions to maximize the yields of the monoarylated intermediate. Two alternative approaches were based on a one-pot reaction concept, which is advantageous from the point of view of costs (solvents, adsorbents etc.) and work-up procedure. Route 2 is based on the stepwise diarylation of **1** without isolation of the monoarylated product. We also expected that simultaneous addition of two different arylboronic acids to the reaction vessel would produce, apart from diarylpyridines **P6** (**4–29**), also the desired unsymmetrical product **P7** (**46–66**) (route 3). A similar idea of an one-pot double cross-coupling strategy was applied by Beaumard et al. [72] in construction of unsymmetrically arylated pyrroles, thiophenes and 2,6- and 3,5-diarylpyridines.

In order to test the utility of the above mentioned approach, a series of trial cross couplings were performed on a microscale according to route 3 with a variety of electron-poor and electron-rich boronic acids (Table 2).

**Table 2:** Results of the preliminary study on the double one-pot arylation of **1** with a mixture of arylboronic acids Ar^1^B(OH)_2_ and Ar^2^B(OH)_2_ **32–41**.

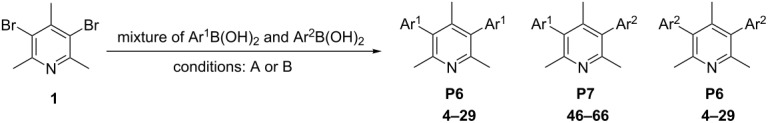						

Entry	Catalytic system^a^	Ar^1^	Ar^2^	Yield^b^ [%]		
				Ar^1^/Ar^1^ pyridine (**P6**)	Ar^1^/Ar^2^ pyridine^c^ (**P7**)	Ar^2^/Ar^2^ pyridine^c^ (**P6**)

1	A	C_6_H_5_ (**32**)	4-EtC_6_H_4_ (**38**)	23 (**4**)	52 (**49**)	25 (**13**)
2	B	C_6_H_5_ (**32**)	4-EtC_6_H_4_ (**38**)	31 (**4**)	52 (**49**)	17 (**13**)
3	A	C_6_H_5_ (**32**)	4-FC_6_H_4_ (**34**)	20 (**4**)	52 (**57**)	28 (**7**)
4	B	C_6_H_5_ (**32**)	4-FC_6_H_4_ (**34**)	17 (**4**)	53 (**57**)	30 (**7**)
5	A	C_6_H_5_ (**32**)	4-MeOC_6_H_4_ (**39**)	34 (**4**)	47 (**58**)	22 (**12**)
6	B	C_6_H_5_ (**32**)	4-MeOC_6_H_4_ (**39**)	30 (**4**)	49 (**58**)	21 (**12**)
7	A	C_6_H_5_ (**32**)	4-CF_3_C_6_H_4_ (**35**)	22 (**4**)	51 (**59**)	27 (**15**)
8	B	C_6_H_5_ (**32**)	4-CF_3_C_6_H_4_ (**35**)	4 (**4**)	34 (**59**)	62 (**15**)
9	A	C_6_H_5_ (**32**)	3,4-OCH_2_OC_6_H_3_ (**40**)	32 (**4**)	49 (**60**)	19 (**21**)
10	B	C_6_H_5_ (**32**)	3,4-OCH_2_OC_6_H_3_ (**40**)	26 (**4**)	50 (**60**)	23 (**21**)
11	A	C_6_H_5_ (**32**)	2-MeC_6_H_4_ (**37**)	36 (**4**)	51 (**46**)	13 (**8**)
12	B	C_6_H_5_ (**32**)	2-MeC_6_H_4_ (**37**)	59 (**4**)	37 (**46**)	4 (**8**)
13	A	C_6_H_5_ (**32**)	2-MeOC_6_H_4_ (**33**)	28 (**4**)	51 (**61**)	21 (**10**)
14	B	C_6_H_5_ (**32**)	2-MeOC_6_H_4_ (**33**)	48 (**4**)	49 (**61**)	8 (**10**)
15	A	C_6_H_5_ (**32**)	2-CF_3_C_6_H_4_ (**41**)	47 (**4**)	43 (**48**)	10 (**31**)
16	B	C_6_H_5_ (**32**)	2-CF_3_C_6_H_4_ (**41**)	–^d^ (**4**)	–^d^ (**48**)	–^d^ (**31**)
17	A	2-MeOC_6_H_4_ (**33**)	4-MeOC_6_H_4_ (**39**)	20 (**10**)	53 (**62**)	27 (**12**)
18	B	2-MeOC_6_H_4_ (**33**)	4-MeOC_6_H_4_ (**39**)	9 (**10**)	48 (**62**)	43 (**12**)
19	A	4-FC_6_H_4_ (**34**)	4-MeOC_6_H_4_ (**39**)	49 (**7**)	43 (**63**)	8 (**12**)
20	B	4-FC_6_H_4_ (**34**)	4-MeOC_6_H_4_ (**39**)	61 (**7**)	36 (**63**)	3 (**12**)
21	A	4-CF_3_C_6_H_4_ (**35**)	3,4-OCH_2_OC_6_H_3_ (**40**)	31 (**15**)	47 (**64**)	22 (**21**)
22	B	4-CF_3_C_6_H_4_ (**35**)	3,4-OCH_2_OC_6_H_3_ (**40**)	64 (**15**)	33 (**64**)	3 (**21**)
23	A	2-ClC_6_H_4_ (**36**)	2-CF_3_C_6_H_4_ (**41**)	30^e,f^ (**30**)	25^e,f^ (**65**)	5^e,g^ (**31**)
24	B	2-ClC_6_H_4_ (**36**)	2-CF_3_C_6_H_4_ (**41**)	27^h,f^ (**30**)	17^h,f^ (**65**)	7^f,g^ (**31**)
25	A	2-MeC_6_H_4_ (**37**)	2-CF_3_C_6_H_4_ (**41**)	37^i,f^ (**8**)	19^i,f^ (**66**)	9^h,i^ (**31**)
26	B	2-MeC_6_H_4_ (**37**)	2-CF_3_C_6_H_4_ (**41**)	–^g^ (**8**)	–^g^ (**66**)	–^i^ (**31**)

^a^Conditions: A – Pd(OAc)_2_, S-Phos, K_3_PO_4_, toluene, 90 °C; B – PdCl_2_(dppf)xCH_2_Cl_2_, dioxane, K_3_PO_4_, 90 °C. ^b^Yields estimated on the base of GC–MS analysis [35]. ^c^Some of these products were tentatively identified by GC–MS – see mass spectra in Supporting Information File 1. ^d^Poor conversion of substrate ~20% – mixture of monoarylated and monobromoarylated products, only traces of **1a** and 3,5-bis(2-trifluorophenyl)-2,4,6-trimethylpyridine were identified. ^e^Reaction mixture contained approx. 40% of monoarylated and monobromoarylated products – as indicated by GC–MS analysis. ^f^The yield of products was calculated by comparison of the peak area of the products with the sum of the peak areas recorded for the precursor (if it was still present). The structures of some monoarylpyridines, bromoarylpyridines and those unsymmetrical diarylpyridines, which had not been synthesized on the preparative scale, were elucidated primarily by the analysis of their mass spectra. Each pyridine derivative exhibited a characteristic mass spectrum with the base peak corresponding to the molecular ion. This feature together with characteristic MS isotopic patterns of the bromine atom led us to recognize the corresponding pyridine derivative. For MS spectra of all tentatively identified pyridines see Supporting Information File 1. ^g^Only a mixture of 3-(2-methylphenyl)-2,4,6-trimethylpyridine and **8** in a ratio of 1:3 was observed. ^h^Reaction mixture contained approx. 50% of monoarylated and monobromoarylated products – as indicated by GC–MS analysis. ^i^Reaction mixture contained approx. 35% of monoarylated and monobromoarylated products – as indicated by GC–MS analysis.

The steric hindrance introduced by *ortho*-substituted boronic acids was also investigated. In order to check how the electronic and steric factors affect the distribution of homo- and heteroarylated products, various combinations of arylboronic acids (**32–41**) were applied as partners for the cross coupling with **1**. Initially, the reactivity of phenylboronic acid (**32**, with a unsubstituted phenyl ring) was chosen as a reference standard in the comparative study. All reactions were performed under two sets of conditions (A = 1.0 equiv of **1**, 1.2 equiv Ar^1^B(OH)_2_, 1.2 equiv Ar^2^B(OH)_2_, 5 mol % Pd(OAc)_2_, 10 mol % S-Phos, 4.0 equiv K_3_PO_4_, toluene; B = 1.0 equiv of **1**, 1.2 equiv Ar^1^B(OH)_2_, 1.2 equiv Ar^2^B(OH)_2_, 5 mol % PdCl_2_(dppf) × CH_2_Cl_2_, 4.0 equiv K_3_PO_4_, dioxane). The reaction mixtures were heated until all the starting material was consumed (monitoring by GC–MS). GC–MS examination of the final crude products revealed in most cases the presence of three major products: symmetrical triaryls **P6** (**4–29**) along with the desired pyridines **P7** (**46–66**) with two different aryl rings. The results were collected in Table 2. Trial reactions with mixtures of phenylboronic acids **32** with *para*-substituted arylboronic acids (**34**, **35**, **38**, **39**, Table 2, entries 1–8) in the presence of Pd(OAc)_2_/S-Phos in toluene led to mixtures of diarylpyridines **4**, **7**, **12**, **13**, **15** and unsymmetrically arylated pyridines **49** and **57–59** generally in a statistical ratio of 1:2:1 for the Ar^1^/Ar^1^:Ar^2^/Ar^1^:Ar^2^/Ar^2^ products, respectively (Table 2, entries 1, 3, 5 and 7). The yield of unsymmetrical diarylpyridines ranged from 47 to 52%. Similar results of differentially substituted heteroaryls were reported by Beaumard et al. [72]. The combination of electron-poor 4-fluorophenylboronic acid (**34**) with phenylboronic acid (**32**) in the presence of PdCl_2_(dppf) × CH_2_Cl_2_ gave a reaction mixture containing an increased concentration of pyridine **7** (Table 2, entry 4), in comparison to **4**. The formation of a similar pyridine derivative was observed as the main product (49% and 61%) when the mixture of 4-fluorophenyl (**34**) and 4-methoxyphenylboronic acid (**39**) were reacted with **1** in the presence of both catalytic systems A or B. It should be noted that in both cases the yield of the desired 3-(4-fluorophenyl)-5-(4-methoxyphenyl)-2,4,6-trimethylpyridine (**63**, Table 2, entry 19 and 20) remained on a reasonable level (43 and 36%). Again, when the reaction was carried out with strong electron-poor 4-(trifluoromethyl)phenylboronic acid (**35**) combined with PhB(OH)_2_ (**32**), the differences in the yield of symmetrical diarylated products were even more pronounced, in favour of the 4-fluoromethyl-substituted derivative **15** (4% for **4** vs 62 % for **15**; Table 2, entry 8). Higher yields of products containing electron-withdrawing groups probably resulted from an increased reactivity of the monobromo intermediate toward the second cross-coupling reaction. This observation was mentioned by Minard et al. [59] in the course of a one-pot two-step Suzuki cross coupling of 3,5-, 2,6-dibromopyridines and dibromobenzenes with various arylboronic partners of different electronic properties.

We then investigated a one-pot, double cross-coupling strategy with mixtures of more sterically demanding boronic acids containing *ortho-*substituents. The cross coupling of **1** with the mixture of 2-methoxyphenyl (**33**) and 4-methoxyphenylboronic acid (**39**) gave a higher yield of the sterically uncrowded product **12** (43%, Table 2, entry 18) as expected. The ratio of products obtained from the mixtures of phenylboronic acid (**32**) with its 2-methyl (**37**), 2-methoxy (**33**) and 2-trifluoromethyl (**41**) derivatives clearly indicate that steric factors play an important role, in each case favouring the synthesis of diphenylpyridine **4** (Table 2, entries 11–15). The formation of the expected unsymmetrical pyridines was still observed in moderate yield (37–51%). Two reactions failed when mixtures of phenyl/2-(trifluoromethyl)phenyl- and 2-methylphenyl/2-(trifluoromethyl)phenylboronic acids were applied in the presence of PdCl_2_(dppf) × CH_2_Cl_2_ (Table 2, entries 16 and 26), respectively. In the case of both sterically demanding reagents, the catalytic system based on S-Phos worked more efficiently giving diarylated products partially contaminated with monoaryl- and monobromoarylpyridines (as was confirmed by GC–MS). These confusing results of the final product distribution cannot be simply rationalized since it is the result of a complicated balance of electronic and steric requirements between both intermediates and arylboronic reagents.

With optimized reaction conditions in hands, we repeated the one-pot double cross-coupling reaction on the 20-fold scale. Synthetic procedures with mixtures of phenyl/4-ethylphenylboronic acid (**32**/**38**) and phenyl/4-methoxyphenylboronic acid (**32**/**39**) gave also mixtures of diarylpyridines with unsymmetrically arylated pyridines **49** and 3-(4-methoxyphenyl)-5-phenyl-2,4,6-trimethylpyridine (**58**) as main products, respectively. Unfortunately, we were not able to obtain the desired compounds in analytically pure form and in reasonable amount (isolated yields less than 5%) due to practical problems associated with chromatographic purification. Their structures were therefore proposed on the basis of GC–MS analysis. Since a stepwise transformation of **1** according to route 2 led to a similar inseparable mixture of symmetrical and unsymmetrical diarylpyridines, we applied route 1 as the way to obtain the desired diarylpyridines **P7** (**46–66**).

Several examples of monoarylation of symmetrical dihaloarenes have been published so far. A low molar ratio of dihaloarene/arylboronic acid often promoted a single coupling [56,74–78]. When the substrate/arylboronic acid ratio was gradually increased, also the increased presence of the dicoupled product was observed. However, the final separated yield decreased and most of the starting material could be recovered. Another important problem in sequential cross-coupling reactions is the proper choice of arylboronic acid for the first reaction. It was confirmed that electron-deficient arylboronic acids activate the monoarylated intermediate for the oxidative addition to the palladium species thus increasing the yield of diarylated byproducts [59].

**Scheme 3:**
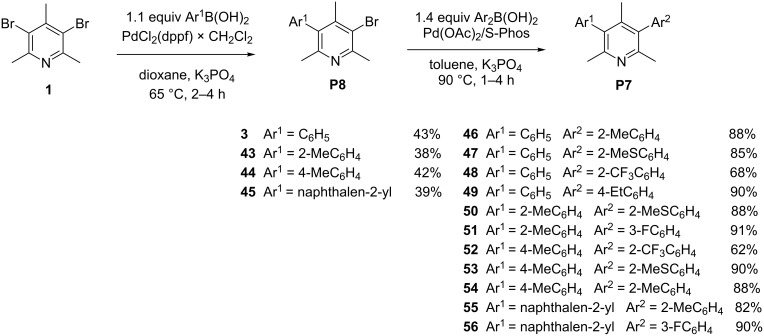
Preparation of unsymmetrical 3,5-diaryl-2,4,6-trimethylpyridines **46–56**.

We then attempted the preparation of unsymmetrical diarylpyridines **46–56**, directly from **1** by two sequential cross couplings with the isolation and full characterization of the intermediates **3** and **43–45**. Thus, the treatment of **1** with 1.1 equiv of phenyl-, 2-methylphenyl-, 4-methylphenyl and 2-naphthylboronic acid in dioxane in the presence of PdCl_2_(dppf) × CH_2_Cl_2_ (5 mol %), and K_3_PO_4_ as a base provided, after 2–4 hours at 65 °C the monoarylated products **3**, **43–45** in moderate yields (38–43%, Scheme 3).

The GC–MS analysis of the crude reaction mixture indicated the presence of the starting material, monoarylbromopyridines and diarylpyridines (**4**, **8**, **9** and **27**) in approx. 1:2:1 ratio. Bromoarylpyridines **3** and **43–45** were then applied for the second cross-coupling process under more drastic conditions. Increasing the temperature to 90 °C and introducing the catalytic system based on Pd(OAc)_2_/S-Phos in toluene, it was possible to obtain the desired unsymmetrical derivatives **46–56**, in a yield range of 82–91%, in less than 1 h, with exception of **48** (68%) and **52** (62%). In these cases the use of sterically hindered and less reactive 2-trifluorophenylboronic acid needed a longer reaction time of 3 hours and an increased arylboronic acid/substrate ratio.

In order to obtain the reference standard for our optimization study, 3-bromo-5-phenyl-2,4,6-trimethylpyridine (**3**) was dehalogenated into **2** in a usual manner by means of ethanol under hydrogen atmosphere in the presence of Pd/C.

### Unsymmetrically substituted 3,5-diaryl-4-chloro-2,6-dimethylpyridines

**Scheme 4:**
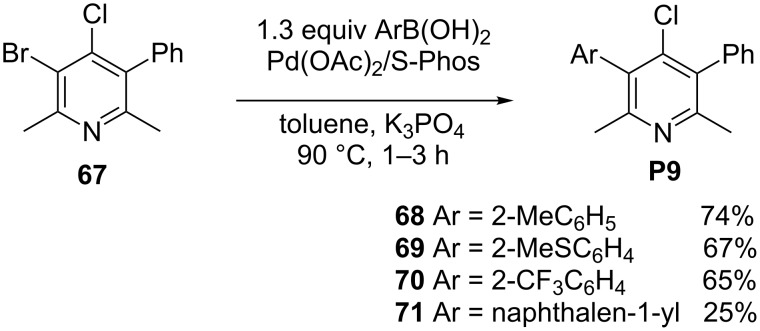
Preparation of unsymmetrical 3,5-diaryl-4-chloro-2,6-dimethylpyridines **68–71**.

Finally, we needed to access a small library of unsymmetrical 3,5-diarylpyridines bearing a chlorine atom at C-4 position at the pyridine ring **P9** (**68–71**, Scheme 4). In a further study we tested, after transhalogenation into 4-bromo derivatives, if pyridines **68–71** were suitable substrates for synthesis of variously substituted 3,4,5-triarylpyridines. Additionally, the dehalogenation of **68–71** may provide 3,5-diarylo-2,6-dimethylpyridines. These compounds can be regarded as "route specific markers" for amphetamine analogues synthesized by the Leuckart method. We choose 3-bromo-4-chloro-2,6-dimethyl-5-phenylpyridine (**67**) as a substrate, whose preparation we described previously [25]. We expected that under mild conditions the position C-3 occupied by the bromine atom would be preferentially arylated despite the fact that generally in the cross-coupling reaction of halopyridines, the reactivity of coupling sites changes in the following order: C-2 > C-4 > C-3. We expected that the presence of the less reactive chlorine atom at C-4 together with the proximity of a bulky phenyl ring should redirect the cross coupling onto position C-3 of the pyridine ring.

Surprisingly, attempted reactions of **67** with both 2-(trifluoromethyl)phenyl (**41**) and (2-methylphenyl)boronic acid (**37**) in dioxane in the presence of PdCl_2_(dppf) × CH_2_Cl_2_, K_3_PO_4_, at 65 °C, provided only a mixture of debrominated and dechloro/debrominated phenylpyridines with only little quantities of the desired products **70**, and **68** (<5%), respectively (estimated by GC–MS). Fortunately, when **67** was applied in the cross-coupling reaction with 1.3 equiv of arylboronic acids in the presence of Pd(OAc)_2_/S-Phos, the desired pyridines **68–70** were isolated in good yields (65–74%). Dehalogenated and diarylated products were also present in the reaction mixture but only in trace amounts. Interestingly, the result of the reaction with naphthalen-1-ylboronic acid was different. The main product was 3,4-bis(naphthalen-1-yl)-2,6-dimethyl-5-phenylpyridine, which was also isolated along with the desired 4-chloro-3-(naphthalen-1-yl)-2,6-dimethyl-5-phenylpyridine (**71**, yield 25%) in 49% yield.

### NMR and GC–MS analysis

All isolated products were characterized by ^1^H NMR, ^13^C NMR, IR spectroscopy and MS and HR-MS spectrometry. The examination of the ^1^H NMR spectra of pyridine **22** revealed the presence the separated signals of methyl groups at position C-2, C-6 and 2-methylthio groups attached to the phenyl rings. A similar separation was observed for all methyl groups which are present in *ortho*-substituted pyridines **8**, **18**, and **26**. This phenomenon may be simply attributed to the restricted rotation around single C_3,5pyridine_–C_phenyl_ bonds caused by the steric interaction of the *ortho* substituents at the phenyl rings (or naphthalene ring for **26**) with three methyl groups of the pyridine ring. Consequently, the inhibited rotation of the phenyl rings led to the formation of stable diastereomeric atropisomers which could be detected in NMR spectroscopy. The interconversion barrier is probably remarkably high in 3,5-bis(2-trifluromethylphenyl)-2,4,6-trimethylpyridine [79], since both atropisomers of this compound could be observed during GC–MS analysis as a pair of well-separated Gaussian peaks at the temperature of elution approaching 290 °C. A similar phenomenon was observed for pyridine **8** and recently in the case of tri-3,4,5-(2-methoxyphenyl)-2,6-dimethylpyridine, and also for some *ortho*-substituted derivatives of 3,5-diaryl-4-methoxy-2,6-dimethylpyridine [32]. In the case of compounds **46–49, 51–54**, and **68–71**, the steric hindrance caused by only one *ortho*-substituted aryl ring should generate the corresponding enentiomeric conformations, stable at least in the NMR time scale. Indeed, the presence of enantiomeric forms of **47** and **69** was confirmed by ^1^H NMR recorded in the presence of (+)-butylphenylphosphinothioic acid [80] as a chiral solvating agent. Recently, we have employed a similar approach in the determination of the enantiomeric purity of (+)-crispine [81] and (*R*)-(+)-harmicine after their stereoselective synthesis [82].

The results of a more detailed study on the stereochemistry of atropisomeric enantiomers will be published elsewhere.

## Conclusion

Herein, we described an easy and convenient synthesis of symmetrically and unsymmetrically substituted 3,5-diaryl-2,4,6-trimethylpyridines via Suzuki–Miyaura cross-coupling reaction. Several diarylpyridines with mixed aryl rings were produced by sequential two-step Suzuki cross-coupling reaction with separation of intermediary 3-aryl-5-bromo-2,4,6-trimethylpyridines. We also proved that a similar set of unsymmetrical products could be accessible via a one-pot double cross coupling by treatment of dihalopyridine with a mixture of arylboronic acids. However, chromatographic separation of the products is tedious and requires more efficient techniques (preparative HPLC). The library of synthesized compounds will be used as a reference standard during our study on impurity markers in illegally produced amphetamine analogues. In several cases of *ortho*-aryl-substituted pyridines, stable atropisomers were observed and this phenomenon will be elaborated in a separate study.

## Supporting Information

File 1 Experimental procedures, spectroscopic and analytical data, and copies of NMR spectra for representative compounds.
