# Supplementary material for: A convenient route to symmetrically and unsymmetrically substituted 3,5-diaryl-2,4,6-trimethylpyridines via Suzuki–Miyaura cross-coupling reaction
Source: Beilstein J Org Chem. 2016 Apr 28;12:835–45. doi: 10.3762/bjoc.12.82 (PMC4901938; doi:10.3762/bjoc.12.82)
Supplement: File 1 — Experimental procedures, spectroscopic and analytical data, and copies of NMR spectra for representative compounds. [file Beilstein_J_Org_Chem-12-835-s001.pdf]

Supporting Information

for

A convenient route to symmetrically and unsymmetrically substituted 3,5-diaryl-2,4,6-trimethylpyridines via Suzuki–Miyaura cross-coupling reaction

Dariusz Błachut[*,1], Joanna Szawkało[2], Zbigniew Czarnocki[2]

Address: [1]Forensic Laboratory, Internal Security Agency, 1 Sierpnia 30A, 02-134 Warsaw, Poland, Tel.: +48 693830760 and [2]Faculty of Chemistry, University of Warsaw, Pasteura 1, 02-093 Warsaw, Poland

Email: Dariusz Błachut - d.blachut@abw.gov.pl

*Corresponding author

Experimental procedures, spectroscopic and analytical data, and copies of NMR spectra for representative compounds

3,5-Dibromo-2,4-6-trimethylpyridine (**1**) [1] and 3-bromo-4-chloro-5-phenyl-2,6-dimethylpyridine (**67**) were prepared according to a previously published procedure [2]. The reagents and solvents were commercially available and were used without further purification. The Suzuki cross coupling reaction was conducted under argon or nitrogen atmosphere in 22 ml vials (Supelco) closed with solid cups sealed with PTFE/silicone septa. TLC analyses were performed on Merck Kieslgel 60 F-254 plates. Visualization of the plates was done under UV light or under iodine vapour. Evaporation of solvents was performed at reduced pressure, using a Büchi rotary evaporator. Melting points were determined on an Electrothermal, Model IA 9200 apparatus and are uncorrected. IR spectra were measured on the Bruker Vortex 70 FTIR spectrometer coupled with Hyperion 2000 microscope. NMR spectra were recorded on a Varian Unity Plus spectrometer operating at 200 MHz for ^{1}H NMR and 50 MHz for ^{13}C NMR or on a Bruker AVANCE 300 MHz spectrometer operating at 300 MHz for ^{1}H NMR and 75 MHz for ^{13}C NMR. The spectra were measured in $CDCl_3$ and are given as δ values (in ppm) relative to TMS. The following abbreviations are used: m - multiplet, s - singlet, d - dublet, t - triplet, q - quartet. Low resolution mass spectra were collected on a Agilent Technologies 7000 Triple Quad mass detector coupled with a Agilent Technologies 7890A gas chromatograph. The column HP-5MS 30 m × 0.25 mm ID, with 0.25 μm film thickness was operated at flow rate of 1 ml/min (helium) and the oven temperature was ramped between 110– 320 °C. The temperature of the injector port was 250 °C. The mass spectra were recorded in the mass range between 40 and 60 amu. HRMS spectra were collected on Quattro LC Micromass and LCT Micromass TOF HiRes apparatuses.

Cross coupling reaction under Suzuki conditions. General procedure:

Pilot study: A vigorously stirred mixture of **1** (50 mg, 0.18 mmol), phenylboronic acid (61 mg, 0.5 mmol, 2.8 equiv), catalytic system (palladium donor and ligand, for quantity see

Table 1), base (4 equiv., see Table 1) in 2–3 ml of solvent system (see Table 1) was heated (oil bath) under argon atmosphere for appropriate time period. For solvent systems based on toluene/H_2O, toluene, 1,4-dioxane or DMF the temperature was adjusted to 90 °C; toluene/H_2O/EtOH was heated at 85°C. The progress of the reaction and the ratio of products/intermediates were monitored by taking a sample 30–60 μl of organic layer, diluting it with toluene, washing with water (2 ml) and, after drying over anhydrous sodium sulfate, analyzing by GC–MS.

Preparative scale: A vigorously stirred mixture of **1** (150 mg, 0.53 mmol), arylboronic acid (2.8 equiv), palladium acetate (4.0 mol %), S-Phos (8.0 mol %) and K_3PO_4 (4.0 equiv) in 10 ml of toluene was heated at 90 °C (oil bath) under inert gas atmosphere for 1–2 hours. The progress of the reaction was monitored by GC–MS. After completion of the process, the mixture was cooled and quenched with cold water (25 ml). The mixture was then extracted with ethyl acetate (3 × 10 ml). Combined organic extracts were dried over anhydrous sodium sulfate and concentrated under reduced pressure. The crude mixture was purified by column chromatography on silica gel (230–400 mesh) with the appropriate solvent system. Pure 3,5-diaryl-4-alkoxy-2,4,6-trimethylpyridines **P6** (**4–29**) were obtained in moderate to good yield.

3,5-Diphenyl-2,4,6-trimethylpyridine (4)

The general procedure described above was used for the reaction of **1** (150 mg, 0.53 mmol) with phenylboronic acid (180 mg, 1.48 mmol) to afford **4** (141 mg, 96%) as a white solid, mp 188-190 ^{o}C.

^{1}H NMR (200 MHz, $CDCl_3$): δ = 7.49 – 7.30 (m, 6H), 7.21 – 7.16 (m, 4H), 2.28 (s, 6H), 1.71 (s, 3H)

^{13}C NMR (50 MHz, $CDCl_3$): δ = 154.2, 143.7, 139.8, 134.7, 129.4, 128.9, 127.3, 23.9, 18.6

MS (EI=70eV) *m/z* (%): 273 (100, $M^{\cdot+}$), 272 (96), 258 (12), 115 (10), 215 (10), 257 (8), 202 (5), 152 (4);

HRMS (ESI+): m/z calcd for $C_{20}H_{19}NH$ $[M+H]^+$ 274.1596; found 274.1590.

3,5-Bis(2-fluorophenyl)-2,4,6-trimethylpyridine (5)

The general procedure described above was applied. Thus, compound **1** (150 mg, 0.53 mmol) was reacted with 2-fluorophenylboronic acid (207 mg, 1.48 mmol) to afford **5** (119 mg, 72%) as a white solid; mp. 162-164 °C.

^{1}H NMR (200 MHz, $CDCl_3$): δ = 7.44 – 7.33 (m, 2H), 7.28 – 7.13 (m, 6H), 2.32 (s, 6H), 1.76 (s, 3H)

^{13}C NMR (50 MHz, $CDCl_3$): δ = 162.3, 157.4, 155.5, 145.2, 131.8, 131.7, 129. 9, 129.7, 128.3, 126.8, 126.4, 124.7, 124.6, 116.3, 115.8, 23.6, 18.1

MS (EI=70eV) *m/z* (%): (309, $M^{\cdot+}$), 294 (66), 133 (17), 289 (12), 251 (7), 213 (5), 170 (4), 107 (4);

HRMS (ESI+): m/z calcd for $C_{20}H_{17}F_2NH$ $[M+H]^+$ 310.1407; found 310.1401.

3,5-Bis(3-fluorophenyl)-2,4,6-trimethylpyridine (6)

The general procedure described above was applied. Thus, compound **1** (150 mg, 0.53 mmol) was reacted with 3-fluorophenylboronic acid (207 mg, 1.48 mmol) to afford **6** (141mg, 85%) as a pale yellow solid; mp 148-149 °C.

^{1}H NMR (200 MHz, $CDCl_3$): δ = 7.48 – 7.37 (m, 2H), 7.13 – 7.03 (m, 2H), 6.98 – 6.88 (m, 4H), 2.29 (s, 6H), 1.72 (s, 3H)

^{13}C NMR (50 MHz, $CDCl_3$): δ = 165.7, 160.8, 154.4, 141.8, 141.7, 133.6, 130.7, 130.5, 125.2, 116.7, 116.3, 114.7, 114.3, 23.9, 18.5

MS (EI=70eV) *m/z* (%): 308 (100), 309 (98, $M^{\cdot+}$), 133 (11), 294 (10), 293 (9) 251 (6), 170 (3), 213 (3);

HRMS (ESI+): m/z calcd for $C_{20}H_{17}F_2NH$ $[M+H]^+$ 310.1407; found 310.1410.

3,5-Bis(4-fluorophenyl)-2,4,6-trimethylpyridine (7)

The general procedure described above was applied. Thus, compound **1** (150 mg, 0.53 mmol) was reacted with 4-fluorophenylboronic acid (207 mg, 1.48 mmol) to afford **7** (143 mg, 86%) as a yellowish solid; mp 198-200 °C;

^1H NMR (200 MHz, $CDCl_3$): δ = 7.16 (bs, 4H), 7.13 (bs, 4H), 2.27 (s, 6H), 1.69 (s, 3H)

^{13}C NMR (50 MHz, $CDCl_3$): δ = 164.7, 159.8, 154.6, 135.5, 135.4, 133.7, 131.1, 130.9, 116.2, 115.8, 23.9, 18.6

MS (EI=70eV) *m/z* (%) 308 (100), 309 (99, $M^{\cdot+}$), 133 (12), 291 (10), 251 (9), 155 (5), 213 (4), 279 (4);

HRMS (ESI+): m/z calcd for $C_{20}H_{17}F_2NH$ $[M+H]^+$ 310.1407; found 310.1413.

3,5-Bis(2-methylphenyl)-2,4,6-trimethylpyridine (8)

The general procedure described above was applied. Thus, compound **1** (150 mg, 0.53 mmol) was reacted with 2-methylphenylboronic acid (201 mg, 1.48 mmol) to afford **8** (113 mg, 70%) as a white solid; mp 145-146 °C;

^1H NMR (300 MHz, $CDCl_3$, two stable atropisomers): δ = 7.33 – 7.23 (m, 6H), 7.10 – 7.02 (m, 2H), 2.22 (2s, 6H), 2.04 (s, 3H), 2.01 (s, 3H), 1.57 (2s, 3H)

^{13}C NMR (75 MHz, $CDCl_3$, two stable atropisomers): δ = 154.1, 154.1, 143.8, 143.7, 139.0, 136.1, 135.9, 133.9, 133.8, 130.4, 129.4, 129.2, 127.7, 126.5, 126.5, 23.4, 23.4, 19.8, 19.7, 17.5

MS (EI=70eV) *m/z* (%): 301 (100, $M^{\cdot+}$), 286 (72), 300 (16), 287 (14), 270 (11), 245 (10), 128 (7), 115 (7);

HRMS (ESI+): m/z calcd for $C_{22}H_{23}NH$ $[M+H]^+$ 302.1909; found 302.1915.

3,5-Bis(4-methylphenyl)-2,4,6-trimethylpyridine (9)

The general procedure described above was applied. Thus, compound **1** (150 mg, 0.53 mmol) was reacted with 4-methylphenylboronic acid (201 mg, 1.48 mmol) to afford **9** (149 mg, 92%) as a white solid; mp 174-176 °C;

^{1}H NMR (300 MHz, $CDCl_3$): δ = 7.26 – 7.05 (m, 8H), 2.40 (s, 6H), 2.28 (s, 6H), 1.71 (s, 3H)

^{13}C NMR (75 MHz, $CDCl_3$): δ = 154.1, 144.0, 136.8, 136.8, 134. 6, 129.6, 129.4, 23.9, 21.4, 18.7

MS (EI=70eV) *m/z* (%): 301 (100, $M^{\cdot+}$), 300 (69), 286 (13), 229 (6), 115 (5), 270 (4), 202 (3), 151 (3);

HRMS (ESI+): m/z calcd for $C_{22}H_{23}NH$ $[M+H]^+$ 302.1909; found 302.1911.

3,5-Bis(2-methoxyphenyl)-2,4,6-trimethylpyridine (10)

The general procedure described above was applied. Thus, compound **1** (150 mg, 0.53 mmol) was reacted with 2-methoxyphenylboronic acid (225 mg, 1.48 mmol) to afford **10** (123 mg, 69%) as a yellowish solid; mp 167-168 °C;

^{1}H NMR (200 MHz, $CDCl_3$): δ = 7.40 – 7.32 (m, 2H), 7.15 – 6.97 (m, 6H), 3.76 (s, 6H), 2.26 (s, 6H), 1.67 (s, 3H)

^{13}C NMR (50 MHz, $CDCl_3$): δ = 156.9, 154.5, 145.1, 131.2, 130.8, 129.0, 128.3, 120.9, 111.1, 55.6, 23.5, 17.9

MS (EI=70eV) *m/z* (%): 333 (100, $M^{\cdot+}$), 302 (28), 318 (14), 272 (11) 288 (8), 167 (5), 151 (4), 131 (4);

HRMS (ESI+): m/z calcd for $C_{22}H_{23}O_2NH$ $[M+H]^+$ 334.1807; found 334.1812.

3,5-Bis(3-methoxyphenyl)-2,4,6-trimethylpyridine (11)

The general procedure described above was applied. Thus, compound **1** (150 mg, 0.53 mmol) was reacted with 3-methoxyphenylboronic acid (225 mg, 1.48 mmol) to afford **11** (152 mg, 85%) as a white solid; mp 141-143 °C;

^{1}H NMR (200 MHz, $CDCl_3$): δ = 7.40 – 7.32 (m, 2H), 6.94 – 6.88 (m, 2H), 6.79 – 6.73 (m, 4H), 3.83 (s, 6H), 2.30 (s, 6H), 1.74 (s, 3H)

^{13}C NMR (50 MHz, $CDCl_3$): δ = 160.0, 154.1, 143.6, 141.1, 134.5, 130.0, 121.8, 115.0, 112.7, 55.4, 23.8, 18.5

MS (EI=70eV) *m/z* (%): 333 (100, $M^{\cdot+}$), 332 (27), 302 (22), 318 (10), 274 (5), 167 (4), 202 (4), 232 (3);

HRMS (ESI+): m/z calcd for $C_{22}H_{23}O_2NH$ $[M+H]^+$ 334.1807; found 334.1816.

3,5-Bis(4-methoxyphenyl)-2,4,6-trimethylpyridine (12)

The general procedure described above was applied. Thus, compound **1** (150 mg, 0.53 mmol) was reacted with 4-methoxyphenylboronic acid (225 mg, 1.48 mmol) to afford **12** (143 mg, 80%) as a white solid; mp 182-184 °C;

^{1}H NMR (200 MHz, $CDCl_3$): δ = 7.97 – 7.09 (m, 8H), 3.86 (s, 6H), 2.28 (s, 6H), 1.72 (s, 3H)

^{13}C NMR (50 MHz, $CDCl_3$): δ = 158.8, 154.4, 144.6, 134.3, 132.0, 130.5, 114.3, 55.4, 23.9, 18.7

MS (EI=70eV) *m/z* (%): 333 (100, $M^{\cdot+}$), 332 (28), 318 (9), 288 (5), 167 (4), 146 (4), 302 (3), 131 (3);

HRMS (ESI+): m/z calcd for $C_{22}H_{23}O_2NH$ $[M+H]^+$ 334.1807; found 334.1803.

3,5-Bis(4-ethylphenyl)-2,4,6-trimethylpyridine (13)

The general procedure described above was applied. Thus, compound **1** (150 mg, 0.53 mmol) was reacted with 4-ethylphenylboronic acid (222 mg, 1.48 mmol) to afford **13** (159 mg, 90%) as a white solid; mp 120-122 °C;

^{1}H NMR (300 MHz, $CDCl_3$): δ = 7.28 – 7.25 (m, 4H), 7.10 – 7. 06 (m, 4H), 2.71 (q, *J* = 7.8 Hz, 4H), 2.28 (s, 6H), 1.72 (s, 3H), 1.29 (t, *J* = 7.8 Hz, 6H)

^{13}C NMR (75 MHz, $CDCl_3$): δ = 154.2, 144.0, 143.1, 137.0, 134.7, 129.3, 128.3, 28.8, 23.9, 18.7, 15.6

MS (EI=70eV) *m/z* (%) 329 (100, $M^{\cdot+}$), 328 (31), 314 (15), 300 (12), 150 (10), 131 (6), 228 (5) 115 (4);

HRMS (ESI+): m/z calcd for $C_{24}H_{27}NH$ $[M+H]^+$ 330.2222; found 330.2228.

3,5-Bis(3-(trifluoromethyl)phenyl)-2,4,6-trimethylpyridine (14)

The general procedure described above was applied. Thus, compound **1** (150 mg, 0.53 mmol) was reacted with 3-(trifluorophenyl)boronic acid (403 mg, 2.12 mmol) to afford **14** (110 mg, 50%) as a pale yellow solid; mp 131-133 °C

^{1}H NMR (300 MHz, $CDCl_3$): δ = 7.69 – 7.59 (m, 4H), 7.51 – 7.49 (m, 2H), 7.43 – 7.40 (m, 2H), 2.30 (s, 6H), 1.70 (s, 3H)

^{13}C NMR (75 MHz, $CDCl_3$): δ = 154.7, 143.5, 140.3, 133.4, 132.92, 132.84, 131.76, 131.71, 131.34, 131.28, 129.61, 129.57, 126.27, 126.21, 126.16, 126.0, 124.5 (q, *J* = 4 Hz, CF_3), 122.4, 23.9, 18.7

MS (EI=70eV) *m/z* (%):409 (100, $M^{\cdot+}$), 408 (90,), 394 (7), 338 (6), 393 (6), 283 (4), 215 (4), 115 (3);

HRMS (ESI+): m/z calcd for $C_{22}H_{17}F_6NH$ $[M+H]^+$ 410.1343; found 410.1348.

3,5-Bis(4-(trifluoromethyl)phenyl)-2,4,6-trimethylpyridine (15)

The general procedure described above was applied. Thus, compound **1** (150 mg, 0.53 mmol) was reacted with 4-(trifluorophenyl)boronic acid (403 mg, 2.12 mmol) to afford **15** (123 mg, 56%) as yellow solid; mp 150-152 °C;

^{1}H NMR (300 MHz, $CDCl_3$): δ = 7.75 – 7.72 (m, 4H), 7.35 – 7.32 (m, 4H), 2.28 (s, 6H), 1.68 (s, 3H)

^{13}C NMR (75 MHz, $CDCl_3$): δ = 154.5, 143.3, 143.2, 133.5, 129.9, 129.7, 126.1 (q, *J* = 4 Hz, CF_3), 122.5, 23.9, 18.6

MS (EI=70eV) *m/z* (%): 408 (100), 409 (95, $M^{\cdot+}$), 394 (8), 393 (7), 338 (7), 283 (5), 115 (4), 215 (3);

HRMS (ESI+): m/z calcd for $C_{22}H_{17}F_6NH$ $[M+H]^+$ 410.1343; found 410.1350.

3,5-Bis(2,5-dimethoxyphenyl)-2,4,6-trimethylpyridine (16)

The general procedure described above was applied. Thus, compound **1** (150 mg, 0.53 mmol) was reacted with 2,5-dimethoxyphenylboronic acid (386 mg, 2.12 mmol) to afford **16** (95 mg, 45%) as a white solid; mp 173-175 °C;

^{1}H NMR (200 MHz, $CDCl_3$): δ = 6.89 – 6.90 (m, 4H), 6.70 – 6.72 (m, 2H), 3.78 (s, 6H), 3.71 (s, 6H), 2.29 (s, 6H), 1.71 (s. 3H)

^{13}C NMR (50 MHz, $CDCl_3$): δ = 154.5, 153.8, 151.2, 145.0, 130.7, 129.2, 117.0, 113.3, 112.1, 56.2, 55.9, 23.4, 17.8

MS (EI=70eV) *m/z* (%): 393 (100, $M^{\cdot+}$), 378 (8), 197 (8), 364 (6), 322 (5), 161, (4) 290 (4); 115 (3);

HRMS (ESI+): m/z calcd for $C_{24}H_{27}O_4NH$ $[M+H]^+$ 394.2018; found 394.2024.

3,5-Bis(3,4,5-trimethoxyphenyl)-2,4,6-trimethylpyridine (17)

The general procedure described above was applied. Thus, compound **1** (150 mg, 0.53 mmol) was reacted with 3,4,5-trimethoxyphenylboronic acid (449 mg, 2.12 mmol) to afford **17** (92 mg, 38%) as a pale brown solid; mp 179-181 °C;

^{1}H NMR (200 MHz, $CDCl_3$): δ = 6.39 (s, 4H), 3.92 (s, 6H), 3.85 (s, 12H), 2.33 (s, 6H), 1.82 (s, 3H)

^{13}C NMR (50 MHz, $CDCl_3$): δ = 154.2, 153.5, 143.8, 137.0, 135.0, 134.5, 106.1, 61.0, 56.2, 23.6, 18.3

MS (EI=70eV) *m/z* (%): 438 (100, $M^{\cdot+}$), 227 (21), 364 (9), 380 (8), 423 (7), 320 (6)278 (6), 182 (5);

HRMS (ESI+): m/z calcd for $C_{26}H_{31}O_6NH$ $[M+H]^+$ 454.2230; found 454.2222.

3,5-Bis(2-ethoxyphenyl)-2,4,6-trimethylpyridine (18)

The general procedure described above was applied. Thus, compound **1** (150 mg, 0.53 mmol) was reacted with 2-ethoxyphenylboronic acid (245 mg, 1.48 mmol) to afford **18** (114 mg, 59%) as a white solid; mp 108-110 °C.

^{1}H NMR (300 MHz, $CDCl_3$, two stable conformers): δ = 7.35 – 7.29 (m, 2H), 7.10 – 7.07 (m, 2H), 7.03 – 6.95 (m, 4H), 4.08 – 3.89 (m, 4H), 2.28 and 2.27 (2s, 6H), 1.68 and 1.67 (2s, 3H), 1.25 and 1.22 (2t, *J* = 9 Hz, 6H)

^{13}C NMR (75 MHz, $CDCl_3$, two stable conformers): δ = 156.3, 156.2, 154.2, 154.2, 145.1, 131.2, 131.1, 131.0, 131.0, 129.0, 128.8, 128.8, 128.8, 121.0, 120.8, 112.8, 112.2, 64.0, 63.7, 23.5, 23.4, 18.0, 17.8, 15.0, 14.9

MS (EI=70eV) *m/z* (%): 361 (100, $M^{\cdot+}$), 288 (16), 345 (15), 332 (13), 328 (13), 304 (112), 372 (11), 202 (5);

HRMS (ESI+): m/z calcd for $C_{24}H_{27}O_2NH$ $[M+H]^+$ 362.2120; found 362.2127.

3,5-Bis(3-ethoxyphenyl)-2,4,6-trimethylpyridine (19)

The general procedure described above was applied. Thus, compound **1** (150 mg, 0.53 mmol) was reacted with 3-ethoxyphenylboronic acid (245 mg, 1.48 mmol) to afford **19** (145 mg, 75%) as a white solid; mp 120-123 °C;

^{1}H NMR (300 MHz, $CDCl_3$, two stable atropisomers): δ = 7.37 – 7.31 (m, 2H), 6.91 – 6.87 (m, 2H), 6.76 – 6.71 (m, 4H), 4.05 and 4.04 (2q, *J* = 6 Hz, 4H), 2.30 (s, 6H), 1.74 (s, 3H), 1.42 and 1.43 (2t, *J* = 6 Hz, 6H)

^{13}C NMR (75 MHz, $CDCl_3$, two stable atropisomers): δ = 159.4, 156.1, 154.0, 143.6, 141.1, 134.6, 129.9, 129.9, 122.2, 121.6, 115.5, 113.3, 63.6, 24.3, 23.8, 23.6, 20.3, 18.4, 15.0

MS (EI=70eV) *m/z* (%): 361 (100, $M^{\cdot+}$), 332 (10), 304, (8), 316 (6), 260 (5), 153, (5), 232 (4), 202 (3);

HRMS (ESI+): m/z calcd for $C_{24}H_{27}O_2NH$ $[M+H]^+$ 362.2120; found 362.2115.

3,5-Bis(4-ethoxyphenyl)-2,4,6-trimethylpyridine (20)

The general procedure described above was applied. Thus, compound **1** (150 mg, 0.53 mmol) was reacted with 4-ethoxyphenylboronic acid (245 mg, 1.48 mmol) to afford **20** (155 mg, 80%) as a pale yellow solid; mp 137-138 °C;

^{1}H NMR (300 MHz, $CDCl_3$): δ = 7.10 – 7.05 (m, 4H), 6.98 – 6.94 (m, 4H), 4.07 (q, *J* = 7 Hz, 4H), 2.28 (s, 6H), 1.72 (s, 3H), 1.44 (t, *J* = 7 Hz, 6H)

^{13}C NMR (75 MHz, $CDCl_3$): δ = 158.2, 154.4, 144.5, 134.3, 131.6, 130.4, 63.6, 23.9, 18.7, 15.1

MS (EI=70eV) *m/z* (%): 361 (100, $M^{\cdot+}$), 304 (13), 332 (10), 131 (7), 274 (5), 202 (3), 248 (3);

HRMS (ESI+): m/z calcd for $C_{24}H_{27}O_2NH$ $[M+H]^+$ 362.2120; found 362.2114.

3,5-Bis(3,4-methylenedioxyphenyl)-2,4,6-trimethylpyridine (21)

The general procedure described above was applied. Thus, compound **1** (150 mg, 0.53 mmol) was reacted with 3,4-methylenedioxyphenylboronic acid (246 mg, 1.48 mmol) to afford **21** (153 mg, 79%) as a yellow solid; mp 160-162 °C;

^{1}H NMR (200 MHz, $CDCl_3$): δ = 6.91 – 6.87 (m, 2H), 6.65 – 6.58 (m, 4H), 6.02 (s, 4H), 2.30 (s, 6H), 1.76 (s, 3H);

^{13}C NMR (50 MHz, $CDCl_3$): δ = 154.5, 148.1, 146.8, 144.5, 134.3, 133.3, 122.6, 109.9, 108.8, 101.3, 23.9, 18.5

MS (EI=70eV) *m/z* (%): 361 (100, $M^{\cdot+}$), 302 (9), 181 (8), 330 (7), 151 (7), 101 (5), 228 (4), 204 (3);

HRMS (ESI+): m/z calcd for $C_{22}H_{19}O_4NH$ $[M+H]^+$ 362.1392; found 362.1399.

3,5-Bis(2-(methylthio)phenyl)-2,4,6-trimethylpyridine (22)

The general procedure described above was applied. Thus, compound **1** (150 mg, 0.53 mmol) was reacted with 2-(methylthio)phenylboronic acid (249 mg, 1.48 mmol) to afford **22** (102 mg, 52%) as pale yellow solid; mp 137-138 °C;

^{1}H NMR (300 MHz, $CDCl_3$, two stable atropisomers): δ = 7.39 – 7.33 (m, 2H), 7.27 – 7.18 (m, 4H), 7.11 – 7.04 (m, 2H), 2.38 and 2.37 (2s, 6H), 2.26 (2s, 6H), 1.66 (s, 3H)

^{13}C NMR (75 MHz, $CDCl_3$, two stable atropisomers): δ = 155.2, 155.1, 145.3, 145.2, 138.8, 138.4, 137.4, 137.4, 132.4, 132.3, 129.8, 129.5, 128.3, 125.0, 124.9, 124.5, 124.2, 23.3, 23.3, 17.3, 17.3, 15.2, 15.0

MS (EI=70eV) *m/z* (%) 365 (100, $M^{\cdot+}$), 350 (98), 288 (14), 320 (11), 305 (8), 143 (7), 159 (5), 335 (4);

HRMS (ESI+): m/z calcd for $C_{22}H_{23}S_2NH$ $[M+H]^+$ 366.1350; found 366.1358.

3,5-Bis(4-(methylthio)phenyl)-2,4,6-trimethylpyridine (23)

The general procedure described above was applied. Thus, compound **1** (150 mg, 0.53 mmol) was reacted with 4-(methylthio)phenylboronic acid (249 mg, 1.48 mmol) to afford **23** (127 mg, 65%) as a yellow solid; mp 125-128 °C;

^{1}H NMR (200 MHz, $CDCl_3$): δ = 7.36 – 7.31 (m, 4H), 7.13 – 7.07 (m, 4H), 2.54 (s, 6H), 2.28 (s, 6H), 1.72 (s, 3H)

^{13}C NMR (50 MHz, $CDCl_3$): δ = 154.3, 144.1, 137.5, 136.3, 134.1, 129.9, 126.8, 23.9, 18.7, 15.8

MS (EI=70eV) *m/z* (%): 365 (100, $M^{\cdot+}$), 183 (8), 332 (7), 317(6), 270 (4), 215 (4), 115 (4), 147 (3);

HRMS (ESI+): m/z calcd for $C_{22}H_{23}S_2NH$ $[M+H]^+$ 366.1350; found 366.1357.

3,5-Bis(2-formylphenyl)-2,4,6-trimethylpyridine (24)

The general procedure described above was applied. Thus, compound **1** (150 mg, 0.53 mmol) was reacted with 2-formylphenylboronic acid (222 mg, 1.48 mmol) to afford **24** (62 mg, 35%) as a pale brown solid; mp 155-157 °C;

^{1}H NMR (200 MHz, $CDCl_3$): δ = 9.83 (s, 2H), 8.09 – 8.05 (m, 2H), 7.77 – 7.69 (m, 2H), 7.62 – 7.54 (m, 2H), 7.29 – 7.25 (m, 2H), 2.26 (s, 6H), 1.61 (s, 3H)

^{13}C NMR (50 MHz, $CDCl_3$): δ = 191.5, 155.2, 144.4, 142.4, 135.0, 133.9, 131.1, 130.7, 128.9, 128.8, 24.0, 18.9

MS (EI=70eV) *m/z* (%): 329 (100, $M^{\cdot+}$), 300 (89), 296 (84), 282 (79), 272 (73), 314 (30), 115 (26), 215 (22);

HRMS (ESI+): m/z calcd for $C_{22}H_{19}O_2NH$ $[M+H]^+$ 330.1494; found 330.1501.

3,5-Bis(2-cyanophenyl)-2,4,6-trimethylpyridine (25)

The general procedure described above was applied. Thus, compound **1** (150 mg, 0.53 mmol) was reacted with 2-cyanophenylboronic acid (217 mg, 1.48 mmol) to afford **25** (73 mg, 42%) as a white solid; mp 201-204 °C;

^{1}H NMR (300 MHz, $CDCl_3$): δ = 7.83 – 7.79 (m, 2H), 7.75 – 7.69 (m, 2H), 7.55 – 7.49 (m, 2H), 7.43 – 7.39 (m, 2H), 2.30 (s, 6H), 1.72 (s, 3H)

^{13}C NMR (75 MHz, $CDCl_3$): δ = 155.5, 143.9, 143.2, 133.6, 133.4, 131.1, 131.0, 128.5, 117.7, 113.3, 23.6, 18.0

MS (EI=70eV) *m/z* (%): 323 (100, $M^{\cdot+}$), 322 (86), 297 (71), 140 (10), 308 (8), 123 (6), 154 (6), 254 (4);

HRMS (ESI+): m/z calcd for $C_{22}H_{17}$N3H $[M+H]^+$ 324.1501; found 324.1506.

3,5-Bis(naphthalen-1-yl)-2,4,6-trimethylpyridine (26)

The general procedure described above was applied. Thus, compound **1** (150 mg, 0.53 mmol) was reacted with 1-napthylphenylboronic acid (254 mg, 1.48 mmol) to afford **26** (160 mg, 80%) as a white solid; mp 226-228 °C;

^{1}H NMR (300 MHz, $CDCl_3$, two stable atropisomers): δ = 7.93 – 7.87 (m, 4H), 7.58 – 7.40 (m, 8H), 7.39 – 7.34 (m, 2H), 2.24 (s, 6H), 1.49 (2s, 3H)

^{13}C NMR (75 MHz, $CDCl_{3,}$ two stable atropisomers): δ = 155.5, 155.5, 146.0, 145.9, 137.1, 137.0, 134.0, 134.0, 132.6, 132.5, 131.9, 128.7, 128.7, 128.0, 127.2, 127.1, 126.7, 126.6, 126.2, 125.9, 125.3, 125.1, 23.6, 18.0, 17.9

MS (EI=70eV) *m/z* (%): 373 (100, $M^{\cdot+}$), 372 (29), 358 (20), 165 (10), 342 (6), 315 (6), 151 (4), 178 (4);

HRMS (ESI+): m/z calcd for $C_{28}H_{23}$NH $[M+H]^+$ 374.1909; found 3743.1916.

3,5-Bis(naphthalen-2-yl)-2,4,6-trimethylpyridine (27)

The general procedure described above was applied. Thus, compound **1** (150 mg, 0.53 mmol) was reacted with 2-naphthylphenylboronic acid (254 mg, 1.48 mmol) to afford **27** (176 mg, 88%) as a white solid; mp 105-107 °C;

^{1}H NMR (300 MHz, $CDCl_3$): δ = 7.95 – 7.84 (m, 6H), 7.69 – 7.68 (m, 2H), 7.53 – 7.49 (m, 4H), 7.35 – 7.32 (m, 2H), 2.35 (s, 6H), 1.77 (s, 3H)

^{13}C NMR (75 MHz, $CDCl_3$): δ = 154.4, 144.1, 137.2, 134.6, 133.8, 132.6, 128.6, 128.2, 128.1, 128.0, 127.7, 126.5, 126.3, 24.0, 18.9

MS (EI=70eV) *m/z* (%): 373 (100, $M^{\cdot+}$), 372 (39), 358 (23), 186 (12), 178 (9), 315 (6), 342 (5), 245 (4);

HRMS (ESI+): m/z calcd for $C_{28}H_{23}NH$ $[M+H]^+$ 374.1909; found 374.1913.

3,5-Bis(5'-methoxynaphthalen-2-yl)-2,4,6-trimethylpyridine (28)

The general procedure described above was applied. Thus, compound **1** (150 mg, 0.53 mmol) was reacted with 5'-methoxy-2-naphthylphenylboronic acid (299 mg, 1.48 mmol) to afford **28** (195 mg, 84%) as a white solid; mp 196-198 °C;

^{1}H NMR (300 MHz, $CDCl_3$): δ = 7.84 – 7.73 (m, 4H), 7.61 – 7.60 (m, 2H), 7.31 – 7.27 (m, 2H), 7.20 – 7.18 (m, 4H), 3.94 (s, 6H), 2.34 (s, 6H), 1.76 (s, 3H)

^{13}C NMR (75 MHz, $CDCl_3$): δ = 158.0, 154.4, 144.3, 135.0, 134.6, 133.7, 129.6, 129.2, 128.2, 128.0, 127.4, 119.3, 105. 9, 55.6, 24.0, 18.9

MS (EI=70eV) *m/z* (%): 433 (100, $M^{\cdot+}$), 217, (18), 418 (8), 389 (6), 152 (6), 253 (5), 346 (4), 152 (3);

HRMS (ESI+): m/z calcd for $C_{30}H_{27}O_2NH$ $[M+H]^+$ 434.2120; found 434.2117.

3,5-Bis(2-aminophenyl)-2,4,6-trimethylpyridine (29)

The general procedure described above was applied. Thus, compound **1** (150 mg, 0.53 mmol) was reacted with 2-aminophenylboronic acid hydrochloride (252 mg, 1.48 mmol) to afford **29** (85 mg, 59%) as a yellowish resin;

^{1}H NMR (300 MHz, $CDCl_3$): δ = 7.22 – 7.16 (m, 2H), 6.94 – 6.91 (m, 2H), 6.85 – 6.78 (m, 4H), 2.53 (s, 6H), 1.79 (s, 3H)

^{13}C NMR (75 MHz, $CDCl_3$): δ = 156.0, 146.8, 143.7, 131.2, 130.1, 128.9, 124.2, 118.9, 115.5, 23.3, 17.2

MS (EI=70eV) *m/z* (%): 303 (100, $M^{\cdot+}$), 288 (93), 271 (16), 130 (12), 151 (8), 247 (6), 103 (5), 172 (4);

HRMS (ESI+): m/z calcd for $C_{20}H_{21}N_3H$ $[M+H]^+$ 304.1814; found 304.1810.

*Monoarylation of **1***: A vigorously stirred mixture of **1** (300 mg, 1.07 mmol), arylboronic acid (1.1 equiv), $PdCl_2$(dppf)xCH_2Cl_2 (5.0 mol %), and K_3PO_4 (2.0 equiv) in 15 ml of dioxane was heated at 65 °C (oil bath) under argon atmosphere for 2–4 hours. The reaction was monitored by GC–MS. After cooling to room temperature, the solvent was evaporated under reduced pressure and water (30 ml) was added to the residue. The aqueous solution was extracted with ethyl acetate (2 × 10 ml). The combined organic layers were dried over anhydrous sodium sulfate and concentrated under reduced pressure. The products were separated and purified by column chromatography.

3-Bromo-5-phenyl-2,4,6-trimethylpyridine (3)

The general procedure described above was applied. Thus, compound **1** (300 mg, 1.07 mmol) was reacted with phenylboronic acid (144 mg, 1.18 mmol) to afford **3** (128 mg, 43%) as a colourless oil;

^{1}H NMR (300 MHz, $CDCl_3$): δ = 7.47 – 7.34 (m, 3H), 7.13 – 7.09 (m, 2H), 2.70 (s, 3H), 2.19 (s, 3H), 2.12 (s, 3H)

^{13}C NMR (75 MHz, $CDCl_3$): δ = 155.3, 153.8, 145.4, 139.1, 135.9, 129.2, 128.9, 127.7, 122.2, 26.0, 23.5, 21.6

MS (EI=70eV) *m/z* (%): 275 (100, $M^{\cdot+}$, ^{79}Br), 277 (99, $M^{\cdot+}$, ^{81}Br), 276 (72, ^{81}Br), 274 (65, ^{79}Br), 195 (13), 153 (15), 181 (11), 115 (10);

HRMS (ESI+): m/z calcd for $C_{14}H_{15}NBrH$ $[M+H]^+$ 276.0388; found 276.0393.

3-Bromo-5-(2-methylphenyl)-2,4,6-trimethylpyridine (43)

The general procedure described above was applied. Thus, compound **1** (300 mg, 1.07 mmol) was reacted with the 2-methylphenylboronic acid (160 mg, 1.18 mmol) to afford **43** (118 mg, 38%) as a colourless oil;

^{1}H NMR (300 MHz, $CDCl_3$): δ = 7.31 – 7.27 (m, 2H), 7.27 – 7.22 (m, 1H), 6.99 – 6.96 (m, 1H), 2.71 (s, 3H), 2.12 (s, 3H), 2.06 (s, 3H), 1.98 (s, 3H)

^{13}C NMR (75 MHz, $CDCl_3$): δ = 155.4, 153.8, 145.5, 138.4, 135.9, 135.1, 130.5, 129.1, 128.1, 126.6, 122.2, 26.0, 23.0, 21.1, 19.7

MS (EI=70eV) *m/z* (%): 289 (100, $M^{\cdot+}$, ^{79}Br), 291 (100, $M^{\cdot+}$, ^{81}Br), 195 (79), 274 (45, ^{79}Br), 276 (44, ^{81}Br), 152 (19), 115 (12), 165 (10);

HRMS (ESI+): m/z calcd for $C_{15}H_{17}NBrH$ $[M+H]^+$ 290.0544; found 290.0538.

3-Bromo-5-(4-methylphenyl)-2,4,6-trimethylpyridine (44)

The general procedure described above was applied. Thus, compound **1** (300 mg, 1.07 mmol) was reacted with 4-methylphenylboronic acid (160 mg, 1.18 mmol) to afford **44** (130 mg, 42%) as a white solid; mp 82-83 °C;

^{1}H NMR (300 MHz, $CDCl_3$): δ = 7.27 – 7.22 (m, 2H), 7.01 – 6.97 (m, 2H), 2.69 (s, 3H), 2.41 (s, 3H), 2.19 (s, 3H), 2.12 (s, 3H)

^{13}C NMR (75 MHz, $CDCl_3$): δ = 155.2, 154.0, 145.6, 137.3, 136.1, 136.0, 129.6, 129.0, 122.2, 26.0, 23.6, 21.7, 21.4

MS (EI=70eV) *m/z* (%): 289 (100, $M^{\cdot+}$, ^{79}Br), 291 (100, $M^{\cdot+}$, ^{81}Br), 195 (48), 153 (21), 274 (17, ^{79}Br), 276 (16, ^{81}Br), 115 (11), 128 (8);

HRMS (ESI+): m/z calcd for $C_{15}H_{17}NBrH$ $[M+H]^+$ 290.0544; found 290.0535.

3-Bromo-5-(naphthalene-2-yl)-2,4,6-trimethylpyridine (45)

The general procedure described above was applied. Thus, compound **1** (300 mg, 1.07 mmol) was reacted with phenylboronic acid (203 mg, 1.18 mmol) to afford **45** (137 mg, 39%) as a pale yellow oil;

^{1}H NMR (300 MHz, $CDCl_3$): δ = 7.94 – 7.82 (m, 3H), 7.60 – 7.50 (m, 3H), 7.25 – 7.22 (m, 1H), 2.73 (s, 3H), 2.22 (s, 3H), 2.15 (s, 3H)

^{13}C NMR (75 MHz, $CDCl_3$): δ = 155.5, 154.0, 145.7, 136.6, 135.9, 133.6, 132.8, 128.8, 128.2, 128.0, 127.3, 126.7, 126.5, 122.3, 26.1, 23.7, 21.8

MS (EI=70eV) *m/z* (%): 325 (100, $M^{\cdot+}$, ^{79}Br), 327 (99, $M^{\cdot+}$, ^{81}Br), 326 (51, ^{81}Br), 324 (39, ^{79}Br), 245 (37), 239 (33), 202 (29), 189 (24); 165 (12);

HRMS (ESI+): m/z calcd for $C_{18}H_{17}NBrH$ $[M+H]^+$ 326.0544; found 326.0550.

Second arylation of 3, 43–45: A vigorously stirred mixture of **3** or **43–45**, (arylboronic acid, 1.4 equiv), $Pd(OAc)_2$ (4 mol %), S-Phos (8 mol %) and K_3PO_4 (3.0 equiv) in toluene was heated at 90 °C (oil bath) under argon atmosphere for 1–4 hours. The reaction was monitored by GC–MS. The mixture was then cooled and quenched with cold water (25–30 ml). The toluene layer was separated and the water layer was additionally extracted with ethyl acetate

(2 × 10 ml). The combined organic extracts were dried over anhydrous sodium sulfate and concentrated under reduced pressure. The products were separated and purified by column chromatography.

3-(2-Methylphenyl)-5-phenyl-2,4,6-trimethylpyridine (46)

The general procedure described above was applied. Thus, compound **3** (70 mg, 0.25 mmol) was reacted with 2-methylphenylboronic acid (43 mg, 0.35 mmol) to afford **46** (64 mg, 88%) as a white solid; mp 117-120 °C;

^{1}H NMR (300 MHz, $CDCl_3$): δ = 7.47 – 7.42 (m, 2H), 7.39 – 7.33 (m, 1H), 7.31 – 7.23 (m, 3H), 7.21 – 7.17 (m, 2H), 7.07 – 7.04 (m, 1H), 2.30 (s, 3H), 2.21 (s, 3H), 2.04 (s, 3H), 1.64 (s, 3H)

^{13}C NMR (75 MHz, $CDCl_3$): δ = 154.1, 154.1, 143.7, 139.7, 139.0, 136.0, 134.7, 133.8, 130.4, 129.5, 129.4, 129.3, 128.9, 128.8, 127.7, 127.3, 126.5, 23.9, 23.4, 19.8, 18.1

MS (EI=70eV) *m/z* (%): 287 (100, $M^{\cdot+}$), 286 (48), 272 (46), 215 (9), 257 (9), 115 (8), 152 (4), 194 (4);

HRMS (ESI+): m/z calcd for $C_{21}H_{22}NH$ $[M+H]^+$ 288.1752; found 288.1745.

3-(2-(Methylthio)phenyl)-5-phenyl-2,4,6-trimethylpyridine (47)

The general procedure described above was applied. Thus, compound **3** (70 mg, 0.25 mmol) was reacted with 2-(methylthio)phenylboronic acid (58 mg, 0.35 mmol) to afford **47** (62 mg, 85%) as a pale yellow solid; mp 114-116 °C;

^{1}H NMR (300 MHz, $CDCl_3$): δ = 7.46 – 7.32 (m, 4H), 7.27 – 7.16 (m, 4H), 7.07 – 7.04 (m, 1H), 2.39 (s, 3H), 2.30 (s, 3H), 2.25 (s, 3H), 1.68 (s, 3H)

^{13}C NMR (75 MHz, $CDCl_3$): δ = 154.7, 154.5, 144.6, 139.6, 138.4, 137.3, 134.7, 132.4, 129.6, 129.6, 129.4, 128.8, 128.8, 128.4, 127.3, 125.0, 124.3, 23.9, 23.1, 17.9, 15.0

MS (EI=70eV) *m/z* (%): 304 (100, $M^{\cdot+}$), 319 (100), 289 (23), 272 (18), 257 (9), 215 (5), 115 (5), 247 (4);

HRMS (ESI+): m/z calcd for $C_{21}H_{22}SNH$ $[M+H]^+$ 320.1473; found 320.1466.

3-(2-(Trifluoromethyl)phenyl)-5-phenyl-2,4,6-trimethylpyridine (48)

The general procedure described above was applied. Thus, compound **3** (70 mg, 0.25 mmol) was reacted with 2-(trifluoromethyl)phenylboronic acid (66 mg, 0.35 mmol) to afford **48** (58 mg, 68%) as a pale yellow solid; mp 113-115 °C;

^{1}H NMR (300 MHz, $CDCl_3$): δ = 7.82 – 7.79 (m, 1H), 7.66 – 7.60 (m, 1H), 7.54 – 7.33 (m, 4H), 7.25 – 7.15 (m, 3H), 2.30 (s, 3H), 2.19 (s, 3H), 1.62 (s, 3H)

^{13}C NMR (75 MHz, $CDCl_3$): δ = 154.9, 154.1, 144.2, 139.4, 138.5, 134.4, 132.5, 131.6, 131.5, 129.6, 129.2, 129.0, 128.8, 128.0, 127.4, 126.7 (q, *J* = 5 Hz, CF_3), 125.9, 122.3, 23.9, 23.6, 18.6

MS (EI=70eV) *m/z* (%): 341 (100, $M^{\cdot+}$), 340 (78), 272 (33), 326 (7), 115 (6), 215 (5), 181 (3), 241 (3);

HRMS (ESI+): m/z calcd for $C_{21}H_{19}F_3NH$ $[M+H]^+$ 342.1470; found 342.1476.

3-(4-Ethylphenyl)-5-phenyl-2,4,6-trimethylpyridine (49)

The general procedure described above was applied. Thus, compound **3** (70 mg, 0.25 mmol) was reacted with 4-ethylphenylboronic acid (52 mg, 0.35 mmol) to afford **49** (69 mg, 90%) as a white solid; mp 145-148 °C;

^{1}H NMR (300 MHz, $CDCl_3$): δ = 7.467 – 7.40 (m, 2H), 7.37 – 7.31 (m, 1H), 7.28 – 7.24 (m, 2H), 7.19 – 7.16 (m, 2H), 7.11 – 7.07 (m, 2H), 2.70 (q, *J* = 6Hz, 2H), 2.30 (s, 3H), 2.28 (s, 3H), 1.72 (s, 3H), 1.28 (t, *J* = 6Hz, 3H)

^{13}C NMR (75 MHz, $CDCl_3$): δ = 154.3, 153.9, 143.8, 143.1, 139.8, 136.8, 134.7, 134.6, 129.4, 129.2, 128.8, 128.3, 127.2, 28.7, 23.9, 23.8, 18.6, 15.6

MS (EI=70eV) *m/z* (%): 287 (100, $M^{\cdot+}$), 286 (48), 272 (46), 215 (9), 257 (9), 115 (8), 152 (4), 194 (4);

HRMS (ESI+): m/z calcd for $C_{22}H_{24}NH$ $[M+H]^+$ 302.1909; found 302.1917.

3-(2-(Methylthio)phenyl)-5-(2-methylphenyl)-2,4,6-trimethylpyridine (50)

The general procedure described above was followed when **43** (75 mg, 0.26 mmol) reacted with the 2-(methylthio)phenylboronic acid (60 mg, 0.36 mmol) to afford **50** (71 mg, 88%) as a pale yellow solid; mp 113-114 ^{o}C;

^1H NMR (300 MHz, $CDCl_3$, two stable atropisomers): δ = 7.40 – 7.34 (m, 1H), 7.30 – 7.19 (m, 5H), 7.10 – 7.04 (m, 2H), 2.39 and 2.37 (2s, 3H), 2.26 and 2.25 (2s, 3H), 2.23 and 2.22 (2s, 3H), 2.04 and 2.03 (2s, 3H), 1.62 and 1.61 (2s, 3H)

^{13}C NMR (75 MHz, $CDCl_3$, two stable atropisomers): δ = 154.6, 154.6, 154.6, 154.5, 144.7, 144.5, 139.0, 138.5, 138.5, 137.4, 136.4, 136.0, 133.8, 132.4, 132.3, 130.4, 130.3, 129.6, 129.6, 129.4, 129.1, 128.3, 127.7, 126.4, 126.4, 125.0, 124.3, 124.3, 23.5, 23.4, 23.2, 23.2, 19.8, 19.7, 17.4, 15.0

MS (EI=70eV) *m/z* (%): 318 (100, $M^{\cdot+}$), 333 (91), 303 (16), 286 (14), 115 (7), 254 (5), 228 (4), 151 (4);

HRMS (ESI+): m/z calcd for $C_{22}H_{24}SNH$ $[M+H]^+$ 334.1629; found 334.1621.

3-(3-Fluorophenyl)-5-(2-methylphenyl)-2,4,6-trimethylpyridine (51)

The general procedure described above was followed and **43** (75 mg, 0.26 mmol) was reacted with 3-fluorophenylboronic acid (50 mg, 0.36 mmol) to afford **51** (70 mg, 91%) as a white solid; mp 113-115 °C;

^1H NMR (300 MHz, $CDCl_3$): δ = 7.45 – 7.38 (m, 1H), 7.33 – 7.24 (m, 3H), 7.10 – 7.03 (m, 2H), 7.00 – 6.90 (m, 2H), 2.30 (s, 3H), 2.21 (s, 3H), 2.03 (2s, 3H), 1.65 (s, 3H)

^{13}C NMR (75 MHz, $CDCl_3$): δ = 164.80, 161.52, 154.58, 153.87, 143.55, 141.97, 141.87, 138.77, 136.01, 135.95, 133.86, 133.54, 133.51, 130.54, 130.51, 130.45, 130.43, 129.26, 129.23, 127.80, 126.55, 126.52, 125.31, 125.27, 125.25, 125.22, 116.66, 116.62, 116.38, 116.34, 114.47, 114.19, 23.79, 23.40, 19.75, 17.97

MS (EI=70eV) *m/z* (%): 305 (100, $M^{\cdot+}$), 304 (53), 290 (52), 275 (8), 233 (8), 115 (6), 152 (5), 194 (3);

HRMS (ESI+): m/z calcd for $C_{21}H_{21}FNH$ $[M+H]^+$ 306.1658; found 306.1650.

3-(2-(trifluoromethyl)phenyl)-5-(4-methylphenyl)-2,4,6-trimethylpyridine 52

The general procedure described above was followed and **44** (80 mg, 0.27 mmol) was reacted with 2-(trifluoromethyl)phenylboronic acid (72 mg, 0.38 mmol) to afford **52** (61 mg, 62%) as a pale yellow oil;

^1H NMR (300 MHz, $CDCl_3$): δ = 7.81 – 7.78 (m, 1H), 7.65 – 7.60 (m, 1H), 7.53 – 7.47 (m, 1H), 7.26 – 7.22 (m, 3H), 7.08 – 7.04 (m, 2H), 2.40 (s, 3H), 2.30 (s, 3H), 2.19 (s, 3H), 1.62 (s, 3H)

^{13}C NMR (75 MHz, $CDCl_3$): δ = 155.1, 153.9, 144.3, 138.6, 137.0, 136.3, 134.4, 132.4, 131.6, 131.5, 129.6, 129.5, 129.4, 129.1, 127.9, 126.7 (q, *J* = 5 Hz, CF_3), 125.9, 122.3, 23.9, 23.6, 21.4, 18.6

MS (EI=70eV) *m/z* (%): 355 (100, $M^{\cdot+}$), 354 (37), 286 (23), 115 (5), 228 (4), 128 (4), 254 (3), 178 (3);

HRMS (ESI+): m/z calcd for $C_{22}H_{21}F_3NH$ $[M+H]^+$ 356.1626; found 356.1634.

3-(2-(Methylthio)phenyl)-5-(4-methylphenyl)-2,4,6-trimethylpyridine (53)

The general procedure described above was followed when **44** (80 mg, 0.27 mmol) reacted with the 2-(methylthio)phenylboronic acid (64 mg, 0.38 mmol) to afford **53** (83 mg, 90%) as a as a pale yellow oil;

^{1}H NMR (300 MHz, $CDCl_3$): δ = 7.39 – 7.34 (m, 1H), 7.26 – 7.18 (m, 4H), 7.12 – 7.04 (m, 3H), 2.40 (s, 3H), 2.38 (s, 3H), 2.30 (s, 3H), 2.24 (s, 3H), 1.68 (s, 3H)

^{13}C NMR (75 MHz, $CDCl_3$): δ = 154.9, 154.3, 144.6, 138.4, 137.5, 136.8, 136.6, 134.6, 132.3, 129.6, 129.5, 129.5, 129.2, 128.3, 125.0, 124.3, 24.0, 23.2, 21.4, 17.9, 15.0

MS (EI=70eV) *m/z* (%): 318 (100, $M^{\cdot +}$), 333 (97), 303 (30), 286 (22), 128 (6), 228 (5), 151 (4), 202 (3);

HRMS (ESI+): m/z calcd for $C_{22}H_{24}SNH$ $[M+H]^+$ 334.1629; found 334.1637.

3-(2-Methylphenyl)-5-(4-methylphenyl)-2,4,6-trimethylpyridine (54)

The general procedure described above was followed when **44** (80 mg, 0.27 mmol) reacted with the 2-methylphenylboronic acid (52 mg, 0.38 mmol) to afford **54** (73 mg, 88%) as a white solid: mp 94-96 °C;

^{1}H NMR (300 MHz, $CDCl_3$): δ = 7.32 – 7.22 (m, 5H), 7.09 – 7.03 (m, 3H), 2.41 (s, 3H), 2.30 (s, 3H), 2.20 (s, 3H), 2.03 (s, 3H), 1.64 (s, 3H)

^{13}C NMR (75 MHz, $CDCl_3$): δ = 154.3, 153.9, 143.9, 139.1, 136.9, 136.7, 136.1, 134.7, 133.8, 130.4, 129.6, 129.5, 129.3, 129.3, 127.7, 126.5, 23.9, 23.4, 21.4, 19.8, 18.1

MS (EI=70eV) *m/z* (%): 301 (100, $M^{\cdot +}$), 286 (36), 300 (31), 270 (5), 229 (4), 115 (4), 202 (3), 152 (3);

HRMS (ESI+): m/z calcd for $C_{22}H_{24}NH$ $[M+H]^+$ 302.1909; found 302.1916.

3-(2-Methylphenyl)-5-(naphthyl-2-yl)-2,4,6-trimethylpyridine (55)

The general procedure described above was followed when **45** (75 mg, 0.23 mmol) reacted with the 2-methylphenylboronic acid (43 mg, 0.32 mmol) to afford **55** (63 mg, 82%) as a as a pale yellow oil;

^{1}H NMR (300 MHz, $CDCl_3$): δ = 7.94 – 7.84 (m, 3H), 7.67 (s, 1H), 7.54 – 7.48 (m, 2H), 7.33 – 7.24 (m, 4H), 7.10 – 7.06 (m, 1H), 2.33 (s, 3H), 2.24 (s, 3H), 2.07 and 2.06 (2s, 3H), 1.67 (s, 3H)

^{13}C NMR (75 MHz, $CDCl_3$): δ = 154.2, 154.2, 143.9, 139.0, 137.2, 137.2, 136,0, 136.0, 134.6, 133.9, 133.7, 132.6, 130.4, 129.3, 128.6, 128.6, 128.2, 128.1, 128.1, 128.0, 127.7, 127.7, 126.5, 126.5, 126.2, 24.0, 23.4, 19.8, 19.8, 18.2, 18.2

MS (EI=70eV) *m/z* (%): 337 (100, $M^{\cdot+}$), 336 (35), 322 (29), 165 (7), 265 (5), 115 (4), 139 (3), 202 (3);

HRMS (ESI+): m/z calcd for $C_{25}H_{24}NH$ $[M+H]^+$ 338.1909; found 338.1900.

3-(3-Fluorophenyl)-5-(naphthyl-2-yl)-2,4,6-trimethylpyridine (56)

The general procedure described above was followed when **45** (75 mg, 0.23 mmol) reacted with the 3-fluorophenylboronic acid (45 mg, 0.32 mmol) to afford **56** (71 mg, 90%) as a yellow oil;

^{1}H NMR (300 MHz, $CDCl_3$): δ = 7.94 – 7.83 (m, 3H), 7.66(br s, 1H), 7.54 – 7.48 (m, 2H), 7.45 – 7.37 (m, 1H), 7.32 – 7.28 (m, 1H), 7.10 – 7.03 (m, 1H), 7.01 – 6.92 (m, 2H), 2.32 (2s, 6H), 1.74 (s, 3H)

^{13}C NMR (75 MHz, $CDCl_3$): δ = 164.8, 161.5, 154.8, 154.0, 143.7, 142.0, 141.9, 137.0, 134.6, 133.7, 133.5, 133.5, 132.6, 130.6, 130.5, 128.7, 128.6, 128.2, 128.1, 128.1, 128.1, 128.0, 128.0, 127.6, 127.5, 126.5, 126.3, 125.2, 125.2, 116.6, 116.3, 114.5, 114.2, 24.0, 23.8, 18.6

MS (EI=70eV) *m/z* (%): 341 (100, $M^{\cdot+}$), 340 (56), 326 (22), 283 (6), 165 (5), 133 (4), 246 (3), 311 (3);

HRMS (ESI+): m/z calcd for $C_{24}H_{21}FNH$ $[M+H]^+$ 342.1658; found 342.1665.

Arylation of 67: A vigorously stirred mixture of **67** (1 equiv), arylboronic acid (1.3 equiv), $Pd(OAc)_2$ (5 mol %), S-Phos (10 mol %) and K_3PO_4 (3.0 equiv) in toluene (8 ml) was heated at 90 °C (oil bath) under argon atmosphere for 1–3 hours. The reaction was monitored by GC–MS. The mixture was than cooled and quenched with cold water (20 ml). The toluene layer was separated and water layer was additionally extracted with ethyl acetate (2 × 5 ml). The combined organic solutions were dried over anhydrous sodium sulphate and concentrated under reduced pressure. The products were separated and purified by column chromatography.

4-Chloro-3-(2-methylphenyl)-5-phenyl-2,6-dimethylpyridine (68)

The general procedure described above was followed when **67** (85 mg, 0.29 mmol) reacted with the 2-methylphenylboronic acid (55 mg, 0.38 mmol) to afford **68** (65 mg, 74%) as a white solid; mp 141-142 °C;

^{1}H NMR (300 MHz, $CDCl_3$): δ = 7.49 – 7.37 (m, 3H), 7.33 – 7.23 (m, 5H), 7.11 – 7.08 (m, 1H), 2.36 (s, 3H), 2.26 (s, 3H), 2.09 (s, 3H)

^{13}C NMR (75 MHz, $CDCl_3$): δ = 156.2, 143.2, 137.6, 137.2, 136.3, 133.5, 132.9, 130.4, 129.6, 129.6, 129.3, 128.8, 128.7, 128.4, 128.0, 126.4, 24.2, 23.7, 19.7

MS (EI=70eV) *m/z* (%): 307 (100, $M^{\cdot+}$, ^{35}Cl)), 309 (32, $M^{\cdot+}$, ^{37}Cl), 292 (30), 215 (17), 272 (12), 115 (8), 152 (5), 181 (5);

HRMS (ESI+): m/z calcd for $C_{20}H_{19}NClH$ $[M+H]^+$ 308.1206; found 3081.1212.

4-Chloro-3-(2-(methylthio)phenyl)-5-phenyl-2,6-dimethylpyridine (69)

The general procedure described above was followed when **67** (85 mg, 0.29 mmol) reacted with the 2-(methylthio)phenylboronic acid (64 mg, 0.38 mmol) to afford **69** (63 mg, 67%) as a as a pale pink oil;

^{1}H NMR (300 MHz, $CDCl_3$): δ = 7.48 – 7.36 (m, 4H), 7.32 – 7.22 (m, 4H), 7.12 – 7.09 (m, 1H), 2.41 (s, 3H), 2.36 (s, 3H), 2.30 (s, 3H)

^{13}C NMR (75 MHz, $CDCl_3$): δ = 156.7, 143.6, 138.4, 137.5, 136.0, 133.5, 131.6, 129.8, 129.7, 129.5, 129.0, 128.7, 128.7, 128.0, 125.2, 24.2, 23.5, 15.5

MS (EI=70eV) *m/z* (%): 304 (100, $M^{\cdot+}$), 309 (32,), 273 (8), 247 (7), 215 (7), 339 (5, $M^{\cdot+}$, ^{35}Cl), 137 (4), 162 (3);

HRMS (ESI+): m/z calcd for $C_{20}H_{19}SNClH$ $[M+H]^+$ 340.0927; found 340.0934.

4-Chloro-3-(2-(trifluoromethyl)phenyl)-5-phenyl-2,6-dimethylpyridine (7)0

The general procedure described above was followed when **67** (85 mg, 0.29 mmol) reacted with the 2-(trifluoromethyl)phenylboronic acid (72 mg, 0.38 mmol) to afford **70** (67 mg, 65%) as a white solid; mp 117-119 °C;

^{1}H NMR (300 MHz, $CDCl_3$): δ = 7.83 – 7.80 (m, 1H), 7.68 – 7.62 (m, 1H), 7.58 – 7.52 (m, 1H), 7.50 – 7.37 (m, 3H), 7.28 – 7.21 (m, 3H), 2.36 (s, 3H), 2.25 (s, 3H)

^{13}C NMR (75 MHz, $CDCl_3$): δ = 157.0, 156.1, 143.5, 137.3, 136.3, 133.3, 132.5, 131.5, 130.8, 129.8, 129.4, 128.9, 128.7, 128.6, 128.1, 126.8 (q, J = 5 Hz, CF_3), 24.2, 23.8

MS (EI=70eV) *m/z* (%): 361 (100, $M^{\cdot+}$), 360 (67,), 273 (8), 292 (24), 215 (10), 326 (8), 115 (5), 142 (4);

HRMS (ESI+): m/z calcd for $C_{20}H_{16}F_3NClH$ $[M+H]^+$ 362.0923; found 362.0933.

4-Chloro-3-(naphthalen-1-yl)-5-phenyl-2,6-dimethylpyridine (71)

The general procedure described above was followed when **67** (85 mg, 0.29 mmol) reacted with the 1-naphtylboronic acid (65 mg, 0.38 mmol) to afford **71** (25 mg, 25%) as a yellow solid; mp 158-160°.

^{1}H NMR (300 MHz, $CDCl_3$): δ = 7.94 – 7.90 (m, 2H), 7.59 – 7.25 (m, 10H), 2.42 (s, 3H), 2.22 (s, 3H)

^{13}C NMR (75 MHz, $CDCl_3$): δ = 157.1, 156.6, 144.0, 137.5, 135.2, 133.9, 133.6, 131.7, 131.6, 129.7, 129.6, 128.8, 128.7, 128.6, 128.0, 127.4, 126.8, 126.3, 125.8, 125.0, 24.3, 23.8

MS (EI=70eV) *m/z* (%): 361 (100, $M^{\cdot+}$), 360 (67,), 273 (8), 292 (24), 215 (10), 326 (8), 115 (5), 142 (4);

HRMS (ESI+): m/z calcd for $C_{23}H_{19}NClH$ $[M+H]^+$ 344.1206; found 344.1214.

Also, the following compound was isolated from the reaction mixture as main compound:

3,4-Bis(naphthalen-1-yl)-5-phenyl-2,6-dimethylpyridine (72) as a pale yellow solid (61 mg, 49 %).

^{1}H NMR (300 MHz, $CDCl_3$): δ = 7.70 – 7.58 (m, 3H), 7.49 – 7.43 (m, 4H), 7.41 – 7.20 (m, 4H), 7.0 – 6.93 (m, 3H), 6.91 – 6.86 (m, 3H), 6.76 – 6.68 (m, 2H), 2.50 (s, 3H), 2.26 (s, 3H)

^{13}C NMR (75 MHz, $CDCl_3$): δ = 156.0, 155.3, 148.3, 138.5, 136.2, 135.9, 134.8, 133.3, 132.6, 132.5, 132.2, 131.6, 130.1, 129.2, 128.4, 127.9, 127.5, 127.4, 127.2, 126.7, 126.6, 126.5, 126.4, 126.2, 125.8, 125.7, 125.4, 125.3, 125.0, 124.1, 24.2, 23.6

MS (EI=70eV) *m/z* (%): 435 (100, $M^{\cdot+}$), 420 (12,), 276 (9), 188 (6), 376 (6), 342 (4), 313 (3), 115 (3);

HRMS (ESI+): m/z calcd for $C_{33}H_{26}NH$ $[M+H]^+$ 436.2065; found 436.2065.

Some products were tentatively identified on the basis of their mass spectra. Most of compounds exhibited characteristic mass spectra with major or at least highly abundant peak corresponding to the molecular ion.

Compound listed in Table 2 (article text):

3-(4-Fluorophenyl)-5-phenyl-2,4,6-trimethylpyridine (57) (entry 3 and 4):

MS (EI=70eV) *m/z* (%): 291 (100, $M^{+\cdot}$), 290 (95), 275 (10), 276 (9), 233 (7), 133 (5), 170 (4), 213 (2).

3-(4-Methoxyphenyl)-5-phenyl-2,4,6-trimethylpyridine (58) (entry 5 and 6):

MS (EI=70eV) *m/z* (%): 303 (100, $M^{+\cdot}$), 302 (45), 288 (12), 258 (8), 272 (6), 244 (5), 146 (4), 131 (4).

3-(4-(Trifluoromethyl)phenyl)-5-phenyl-2,4,6-trimethylpyridine (59) (entry 7 and 8):

MS (EI=70eV) *m/z* (%): 341 (100, $M^{+\cdot}$), 340 (97), 115 (15), 326 (13), 215 (10), 325 (9), 337 (4), 283 (3).

3-(3,4-Methylenedioxyphenyl)-5-phenyl-2,4,6-trimethylpyridine (60) (entry 9 and 10):

MS (EI=70eV) *m/z* (%): 317 (100, $M^{+\cdot}$), 316 (38), 258 (11), 286 (7), 202 (5), 286 (5), 101 (4), 228 (4).

3-(2-Methoxyphenyl)-5-phenyl-2,4,6-trimethylpyridine (61) (entry 13 and 14):

MS (EI=70eV) *m/z* (%): 303 (100, $M^{+\cdot}$), 302 (32), 272 (30), 283 (15), 115 (8), 215 (7), 151 (4), 131 (3).

3,5-Bis(2-(trifluoromethyl)phenyl)-2,4,6-trimethylpyridine (31) (entry 15, Ar_2/Ar_2 pyridine):

MS (EI=70eV) *m/z* (%): 409 (78, $M^{\cdot +}$), 340 (100), 164 (5), 133 (5), 195 (4), 183 (3), 394 (3), 226 (3).

3-(2-Methoxyphenyl)-5-(4-methoxyphenyl)-2,4,6-trimethylpyridine (62) (17 and 18):

MS (EI=70eV) *m/z* (%): 333 (100, $M^{+\cdot}$), 332 (15), 302 (11), 318 (9), 131 (5), 288 (5), 272 (3), 146 (3).

3-(4-Fluorophenyl)-5-(4-methoxyphenyl)-2,4,6-trimethylpyridine (63) (entry 19 and 20):

MS (EI=70eV) *m/z* (%): 321 (100, $M^{+\cdot}$), 320 (44), 306 (11), 276 (8), 262 (7), 290 (7), 170 (5), 146 (3).

3-(3,4-Methylenedioxyphenyl)-5-(4-(trifluoromethyl)phenyl)-2,4,6-trimethylpyridine (64) (entry 21 and 22):

MS (EI=70eV) *m/z* (%): 385 (100, $M^{+\cdot}$), 384 (28), 326 (11), 354 (6), 366 (6), 312 (5), 228 (3), 102 (3).

3,5-Bis(2-chlorophenyl)-2,4,6-trimethylpyridine (30) (entry 23 and 24, Ar_1/Ar_1 pyridine):

MS (EI=70eV) *m/z* (%): 307 (100, ^{35}Cl), 342 (36, $M^{+\cdot}$, ^{35}Cl,^{35}Cl), 309 (35, ^{37}Cl), 344 (26, ^{37}Cl, ^{37}Cl), 256 (7), 128 (6), 271 (5), 136 (4).

3-(2-Methylphenyl)-5-(2-(trifluoromethyl)phenyl)-2,4,6-trimethylpyridine (66) (entry 25 and 26, Ar_1/Ar_2 pyridine):

MS (EI=70eV) *m/z* (%): 355 (100, $M^{+\cdot}$), 340 (61), 286 (32), 325 (8), 115 (6), 128 (5), 270 (4), 299 (4).

3-(2-(Trifluoromethyl)phenyl)-2,4,6-trimethylpyridine (entry 25 and 26, monoaryl pyridine)**:**

MS (EI=70eV) *m/z* (%): 265 (100, $M^{+\cdot}$), 196 (84), 195 (15), 181 (13), 164 (8) 133 (7), 226 (4), 183 (3).

3-(2-Methylphenyl)-2,4,6-trimethylpyridine (entry 25 and 26, monoaryl pyridine):

MS (EI=70eV) *m/z* (%): 211 (100, $M^{+\cdot}$), 196 (88), 210 (14), 195 (13), 181 (13), 128 (11), 127 (7), 115 (4).

3-Bromo-5-(2-(trifluoromethyl)phenyl)-2,4,6-trimethylpyridine (entry 23 and 24, monoarylbromopyridine):

MS (EI=70eV) *m/z* (%): 345 (100, $M^{+\cdot}$, ^{81}Br), 343 (99, $M^{+\cdot}$, ^{79}Br), 274 (46), 276 (42), 195 (12), 183 (9).

References

[1] Drzeniek, W., Tomasik, P., *Roczniki Chemii* **1970**, *44*, 779 - 783.

[2] D. Błachut, Z. Czarnocki, K. Wojtasiewicz, *Synthesis* **2006**, *17*, 2855-2864

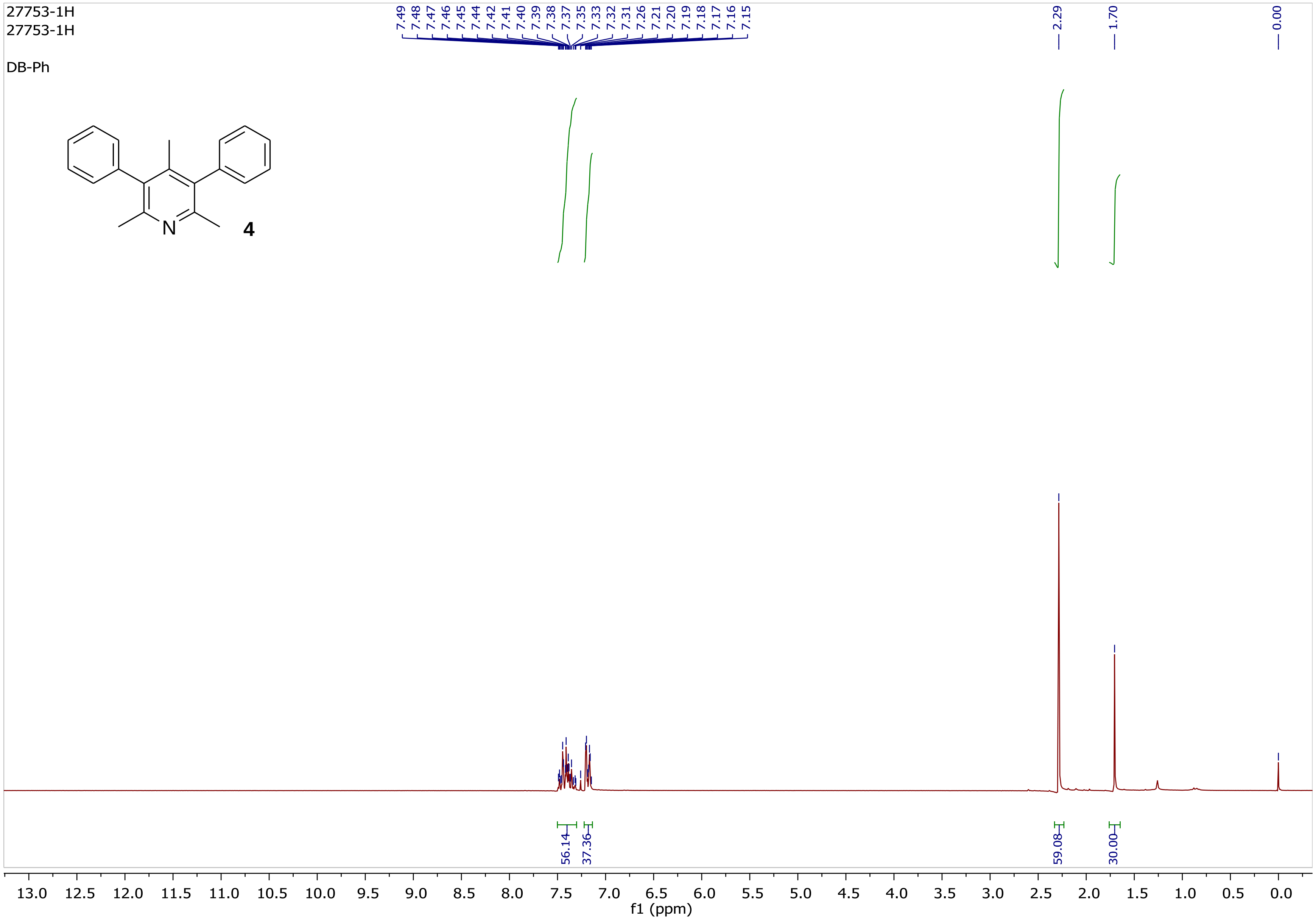
27753-1H
27753-1H
DB-Ph
4
N
7.49 7.48 7.47 7.46 7.45 7.44 7.42 7.41 7.40 7.39 7.38 7.37 7.35 7.33 7.32 7.31 7.26 7.21 7.20 7.19 7.18 7.17 7.16 7.15
2.29
1.70
0.00
56.14
37.36
59.08
30.00
13.0 12.5 12.0 11.5 11.0 10.5 10.0 9.5 9.0 8.5 8.0 7.5 7.0 6.5 6.0 5.5 5.0 4.5 4.0 3.5 3.0 2.5 2.0 1.5 1.0 0.5 0.0
f1 (ppm)

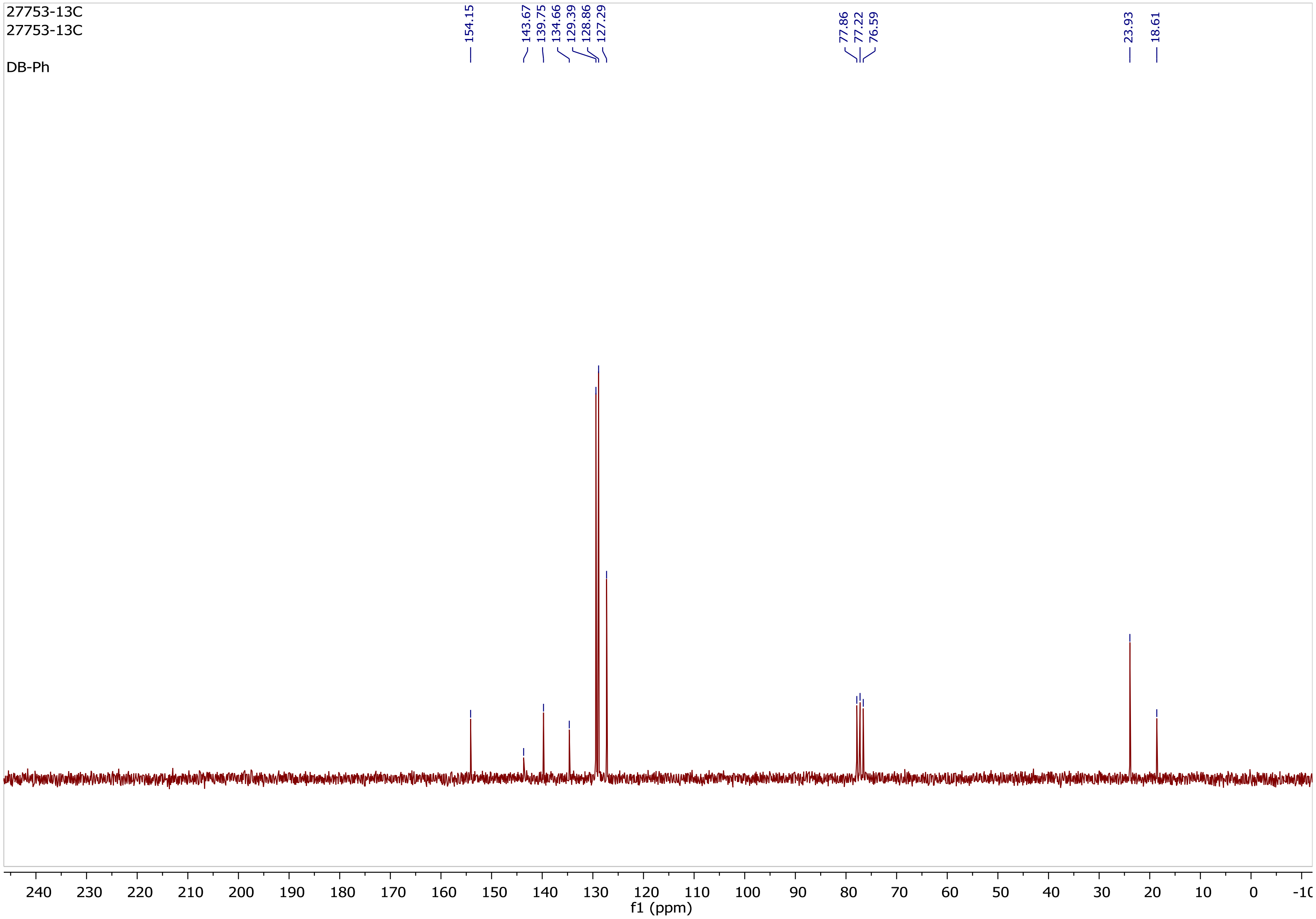
27753-13C
27753-13C
DB-Ph
154.15
143.67
139.75
134.66
129.39
128.86
127.29
77.86
77.22
76.59
23.93
18.61
240 230 220 210 200 190 180 170 160 150 140 130 120 110 100 90 80 70 60 50 40 30 20 10 0 -10
f1 (ppm)

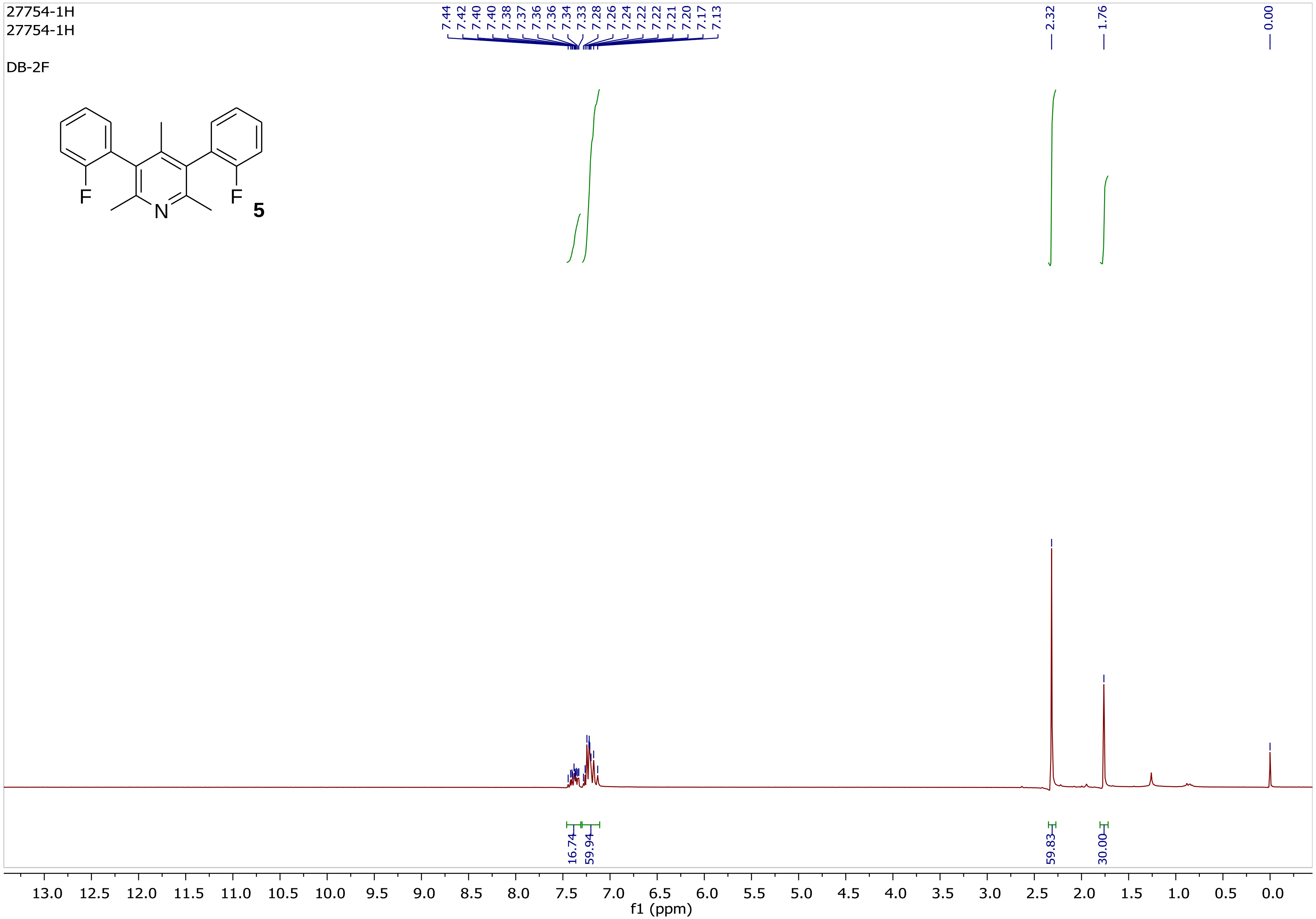
27754-1H
27754-1H
DB-2F
5
7.44
7.42
7.40
7.40
7.38
7.37
7.36
7.36
7.34
7.33
7.28
7.26
7.24
7.22
7.22
7.21
7.20
7.17
7.13
2.32
1.76
0.00
16.74
59.94
59.83
30.00
13.0 12.5 12.0 11.5 11.0 10.5 10.0 9.5 9.0 8.5 8.0 7.5 7.0 6.5 6.0 5.5 5.0 4.5 4.0 3.5 3.0 2.5 2.0 1.5 1.0 0.5 0.0
f1 (ppm)

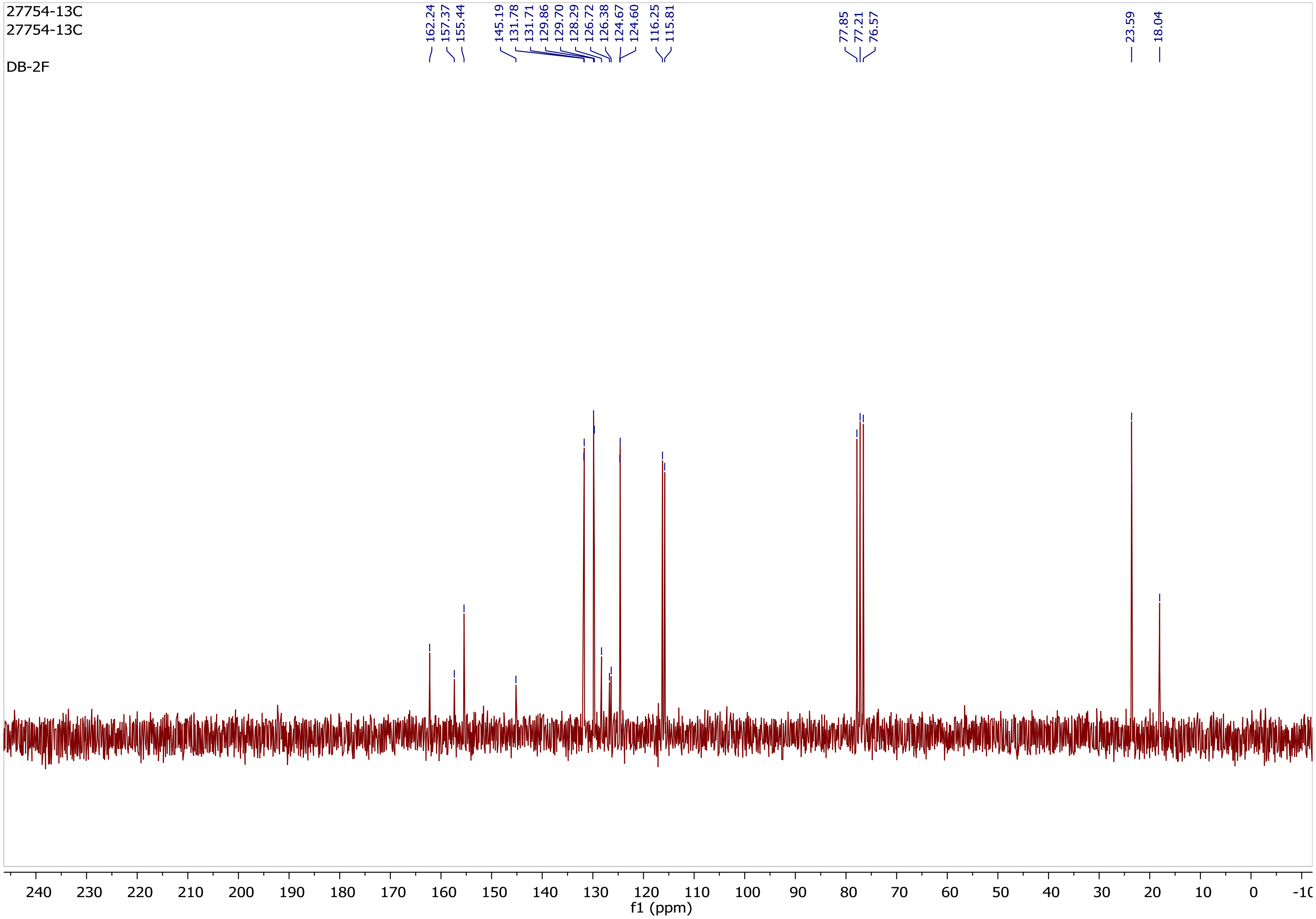
27754-13C
27754-13C
DB-2F
162.24
157.37
155.44
145.19
131.78
131.71
129.86
129.70
128.29
126.72
126.38
124.67
124.60
116.25
115.81
77.85
77.21
76.57
23.59
18.04
240
230
220
210
200
190
180
170
160
150
140
130
120
110
100
90
80
70
60
50
40
30
20
10
0
-10
f1 (ppm)

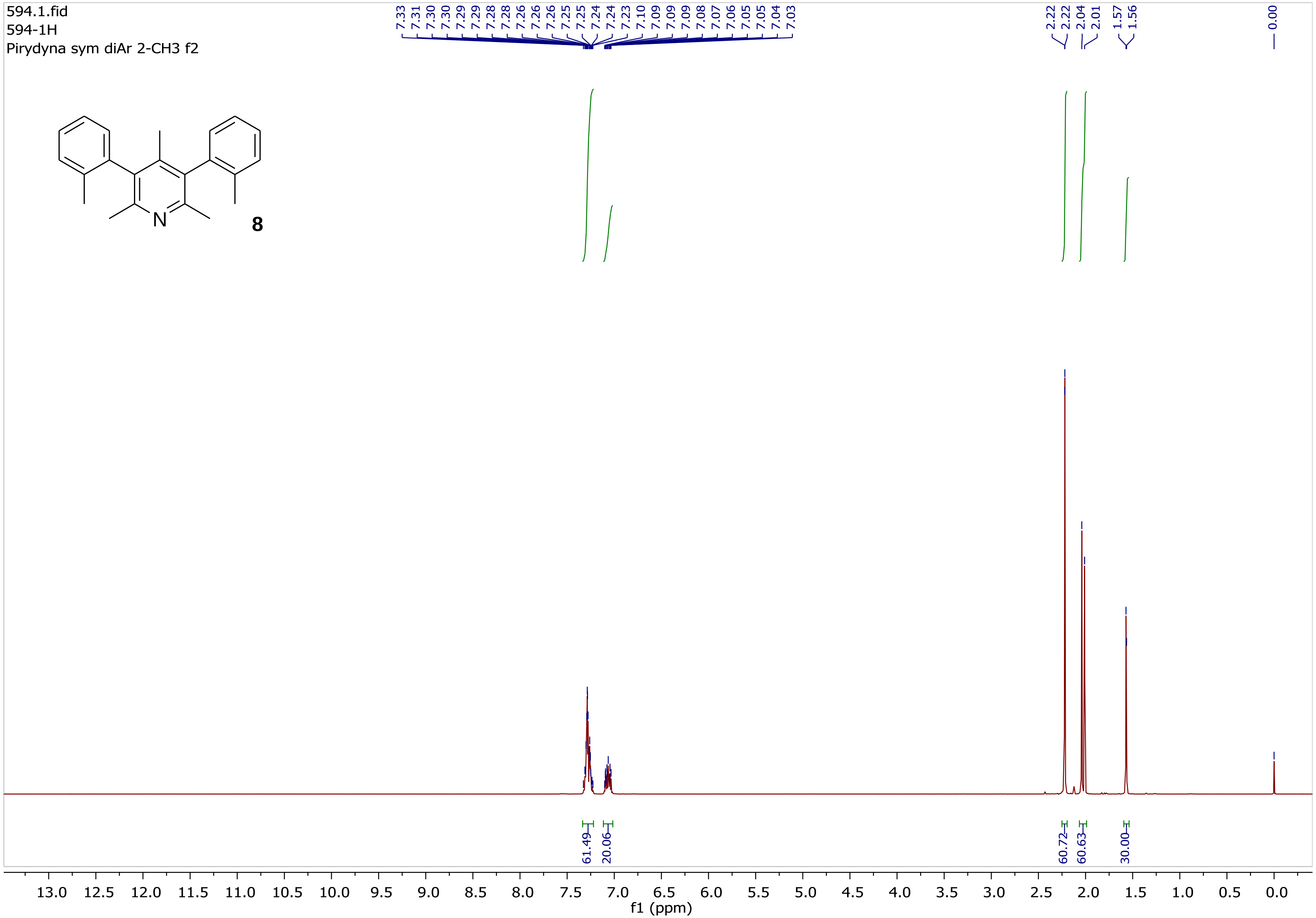
594.1.fid
594-1H
Pirydyna sym diAr 2-CH3 f2
8
N
7.33 7.31 7.30 7.30 7.29 7.29 7.28 7.28 7.26 7.26 7.26 7.25 7.25 7.24 7.24 7.23 7.10 7.09 7.09 7.09 7.08 7.07 7.06 7.05 7.05 7.04 7.03
2.22 2.22 2.04 2.01 1.57 1.56
0.00
61.49 20.06 60.72 60.63 30.00
13.0 12.5 12.0 11.5 11.0 10.5 10.0 9.5 9.0 8.5 8.0 7.5 7.0 6.5 6.0 5.5 5.0 4.5 4.0 3.5 3.0 2.5 2.0 1.5 1.0 0.5 0.0
f1 (ppm)

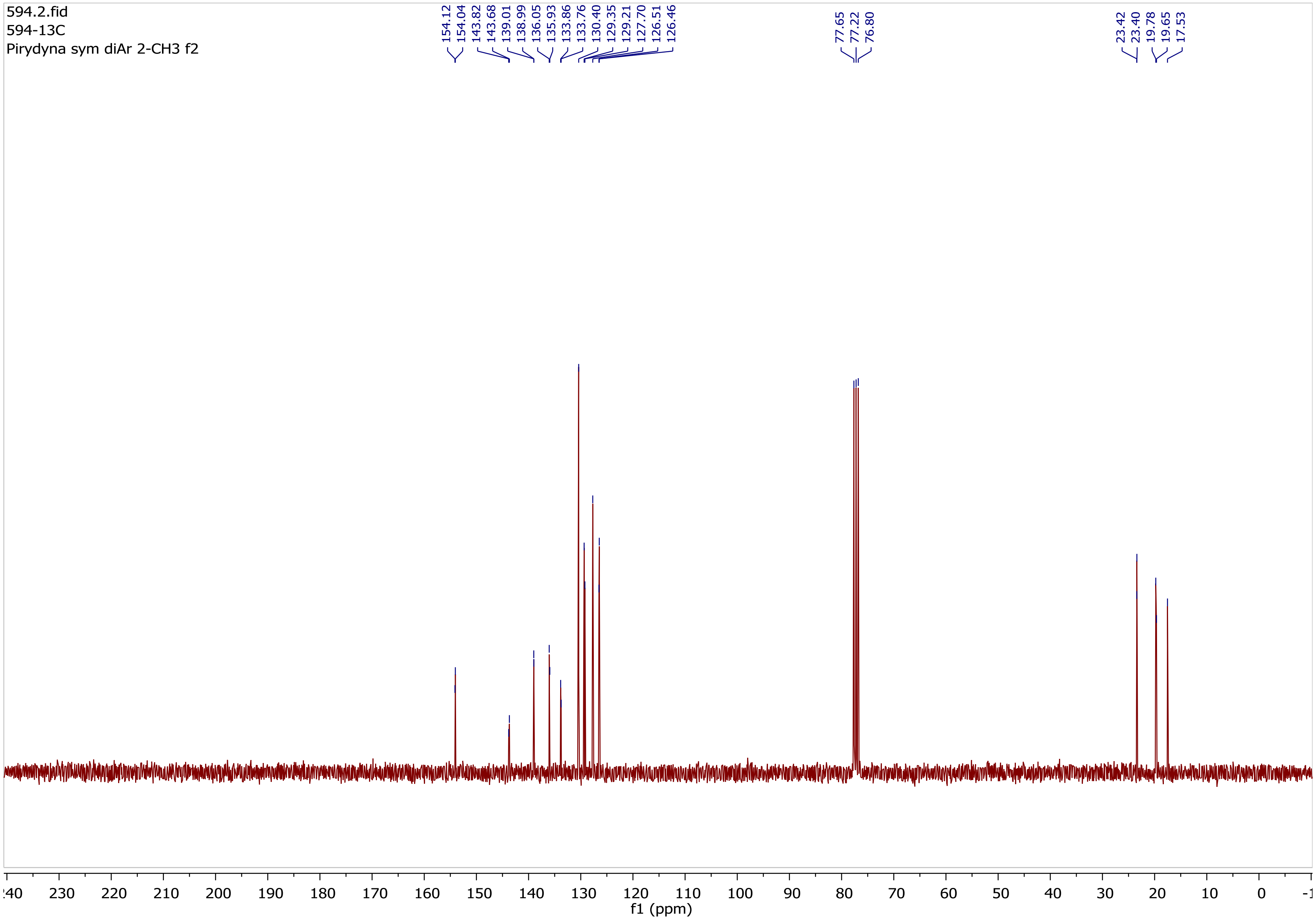
594.2.fid
594-13C
Pirydyna sym diAr 2-CH3 f2
154.12
154.04
143.82
143.68
139.01
138.99
136.05
135.93
133.86
133.76
130.40
129.35
129.21
127.70
126.51
126.46
77.65
77.22
76.80
23.42
23.40
19.78
19.65
17.53
f1 (ppm)

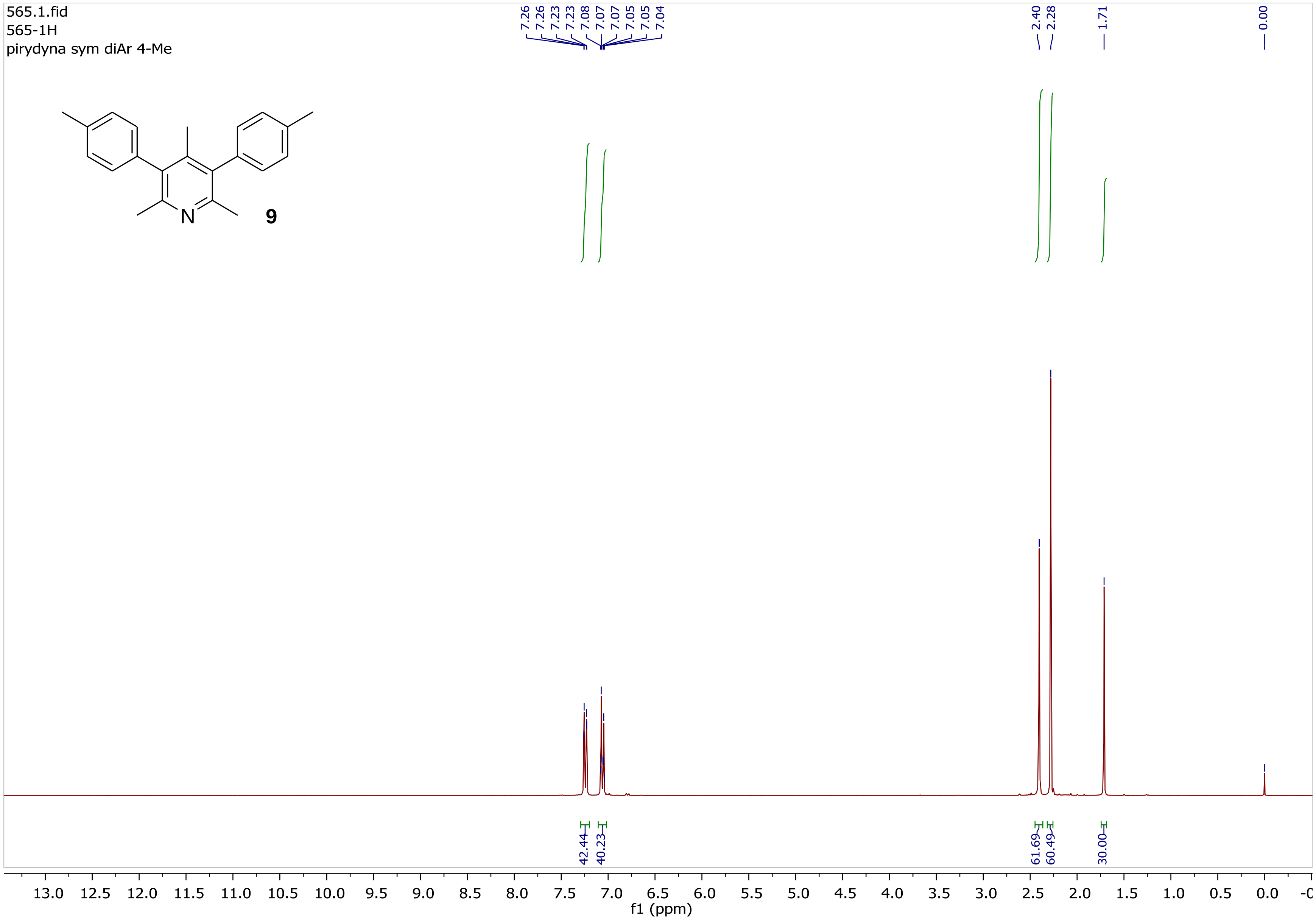
565.1.fid
565-1H
pirydyna sym diAr 4-Me
9
7.26
7.26
7.23
7.23
7.08
7.07
7.07
7.05
7.05
7.04
2.40
2.28
1.71
0.00
42.44
40.23
61.69
60.49
30.00
13.0 12.5 12.0 11.5 11.0 10.5 10.0 9.5 9.0 8.5 8.0 7.5 7.0 6.5 6.0 5.5 5.0 4.5 4.0 3.5 3.0 2.5 2.0 1.5 1.0 0.5 0.0
f1 (ppm)

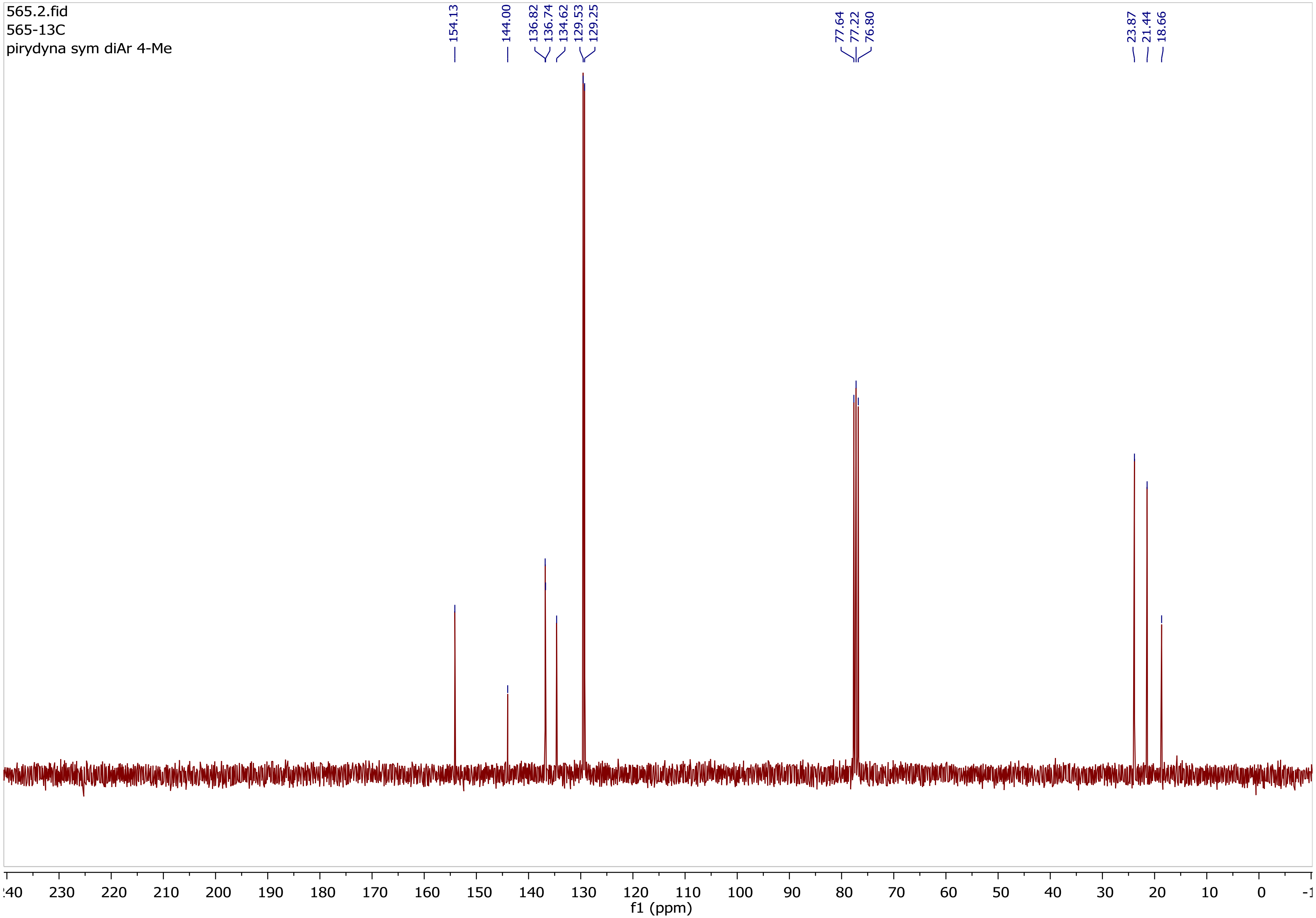
565.2.fid
565-13C
pirydyna sym diAr 4-Me
154.13
144.00
136.82
136.74
134.62
129.53
129.25
77.64
77.22
76.80
23.87
21.44
18.66
240 230 220 210 200 190 180 170 160 150 140 130 120 110 100 90 80 70 60 50 40 30 20 10 0 -1
f1 (ppm)

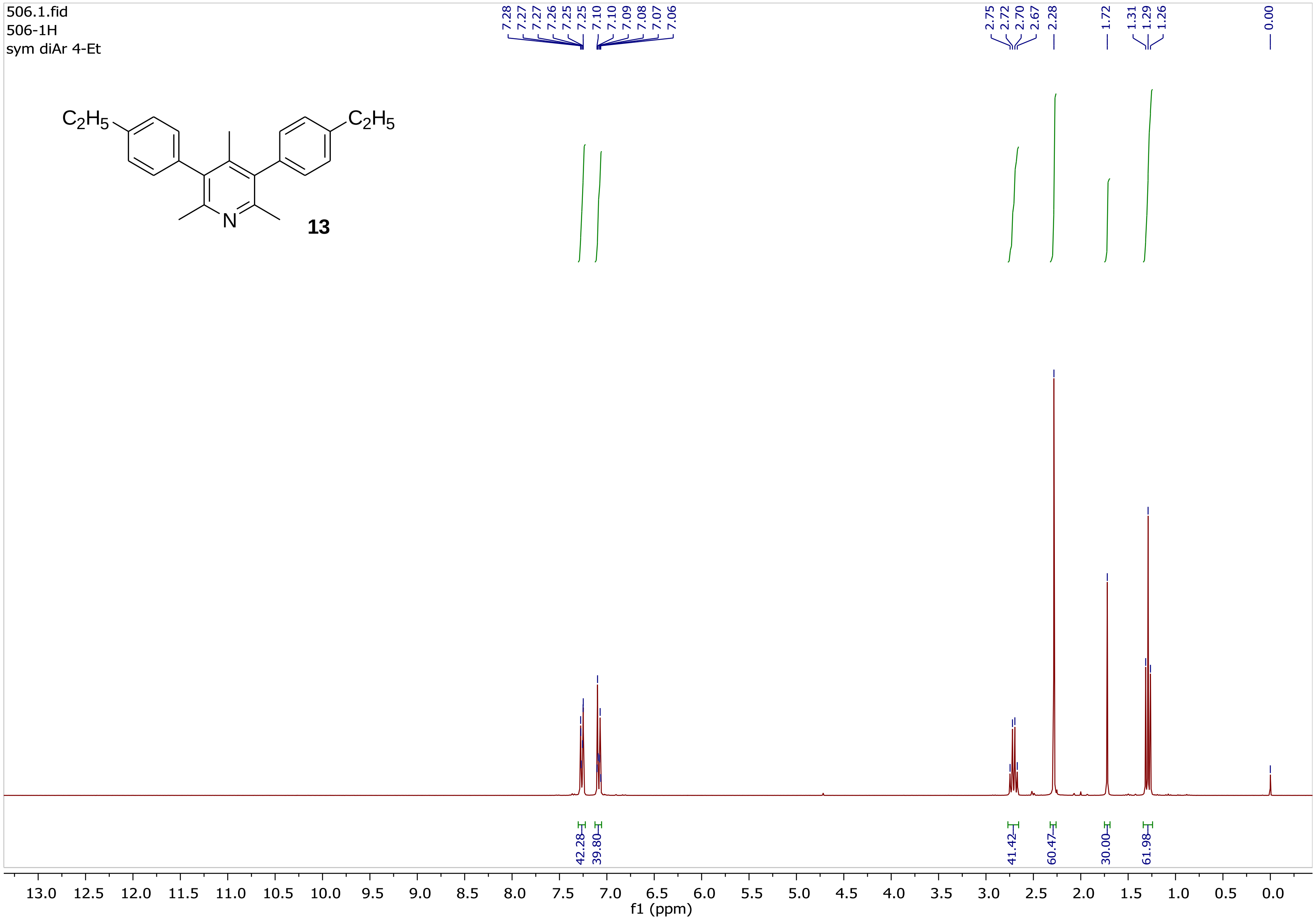

506.1.fid
506-1H
sym diAr 4-Et
7.28
7.27
7.27
7.26
7.25
7.25
7.10
7.10
7.09
7.08
7.07
7.06
2.75
2.72
2.70
2.67
2.28
1.72
1.31
1.29
1.26
0.00
C2H5
C2H5
N
13
42.28
39.80
41.42
60.47
30.00
61.98
13.0 12.5 12.0 11.5 11.0 10.5 10.0 9.5 9.0 8.5 8.0 7.5 7.0 6.5 6.0 5.5 5.0 4.5 4.0 3.5 3.0 2.5 2.0 1.5 1.0 0.5 0.0
f1 (ppm)

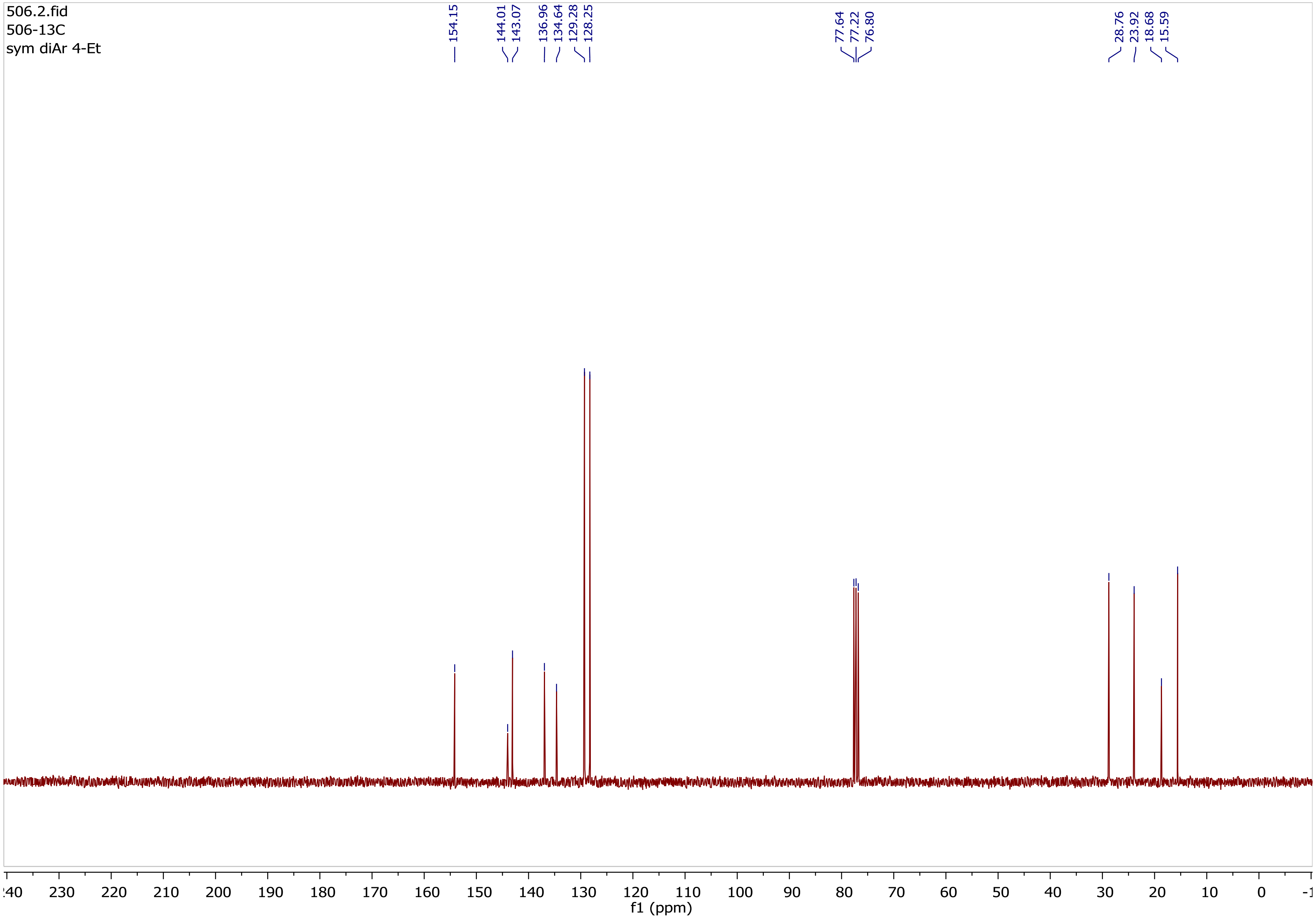
506.2.fid
506-13C
sym diAr 4-Et
154.15
144.01
143.07
136.96
134.64
129.28
128.25
77.64
77.22
76.80
28.76
23.92
18.68
15.59
f1 (ppm)

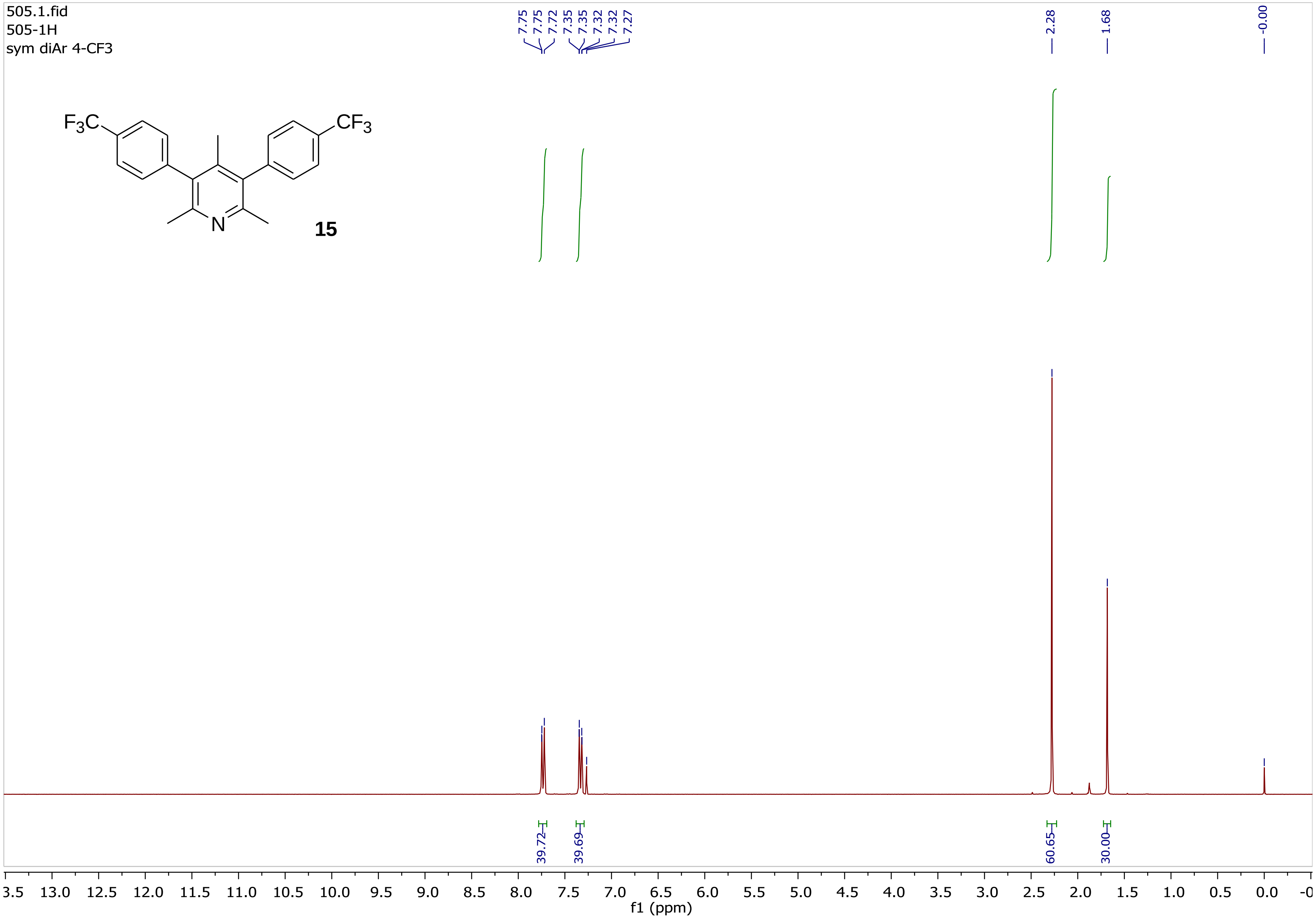
505.1.fid
505-1H
sym diAr 4-CF3
F_3C
CF_3
N
15
7.75
7.75
7.72
7.35
7.35
7.32
7.32
7.27
2.28
1.68
-0.00
39.72
39.69
60.65
30.00
f1 (ppm)

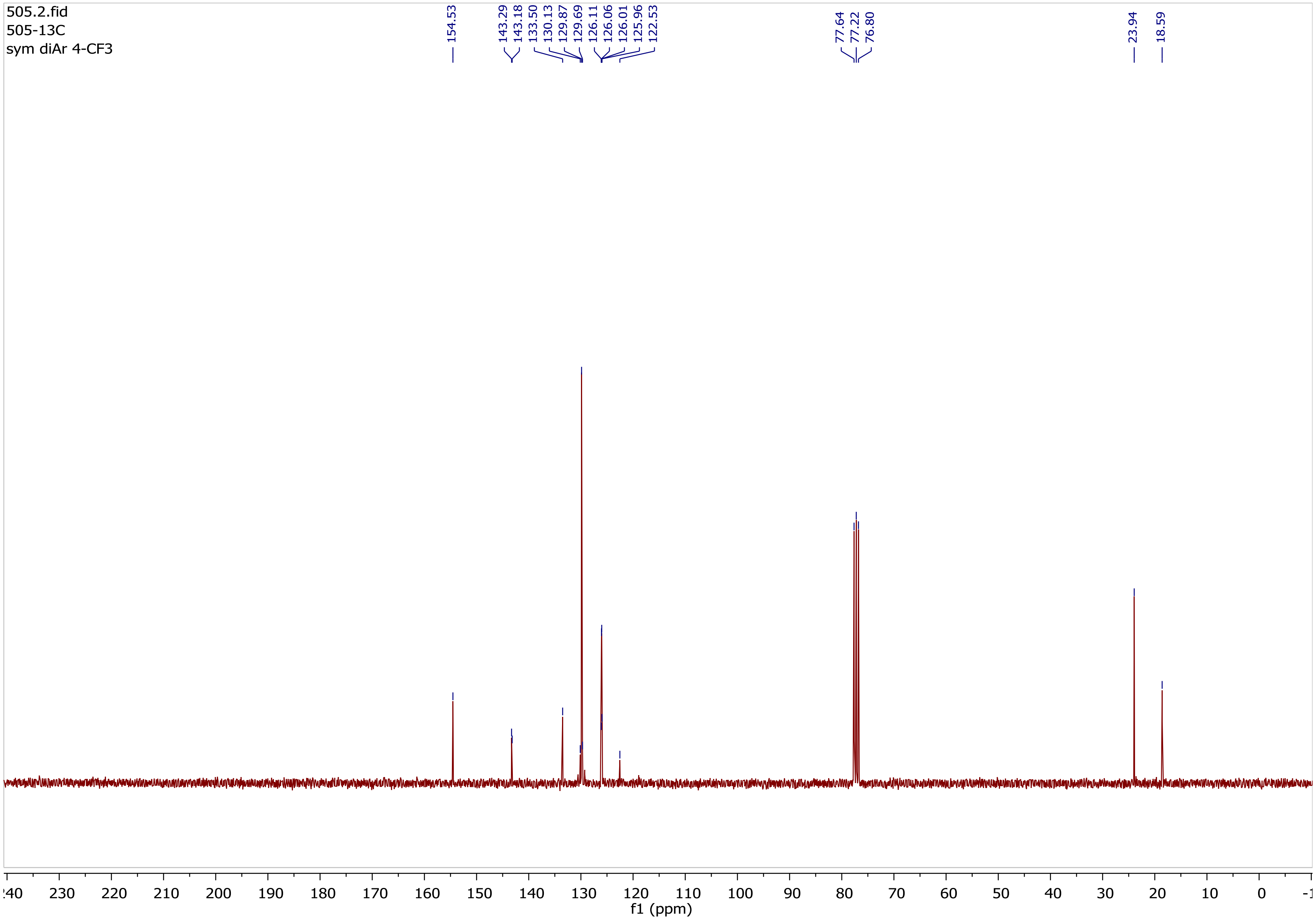
505.2.fid
505-13C
sym diAr 4-CF3
154.53
143.29
143.18
133.50
130.13
129.87
129.69
126.11
126.06
126.01
125.96
122.53
77.64
77.22
76.80
23.94
18.59
f1 (ppm)

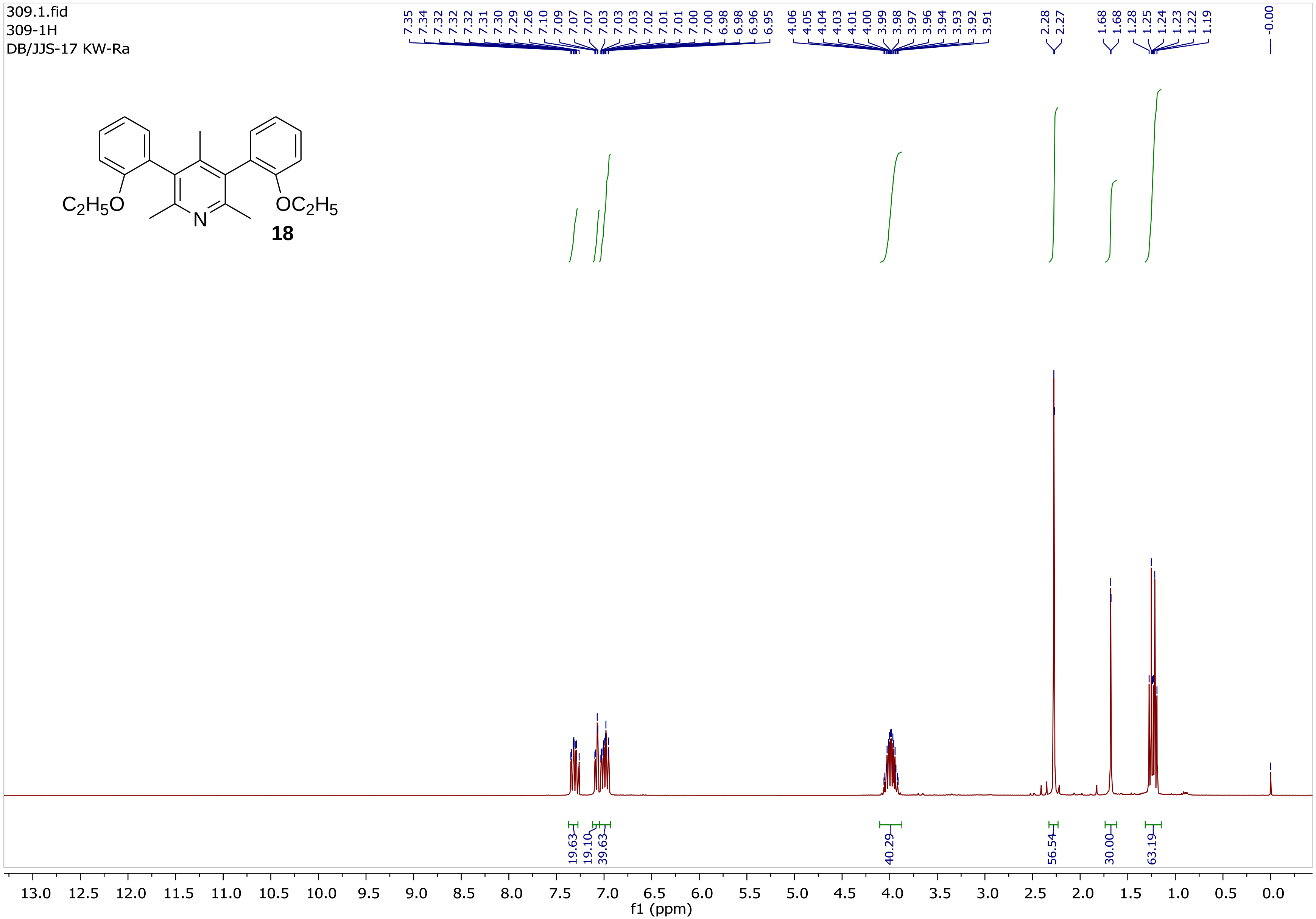
309.1.fid
309-1H
DB/JJS-17 KW-Ra
7.35 7.34 7.32 7.32 7.32 7.31 7.30 7.29 7.26 7.10 7.09 7.07 7.07 7.03 7.03 7.03 7.02 7.01 7.01 7.00 7.00 6.98 6.96 6.95
4.06 4.05 4.04 4.03 4.01 4.00 3.99 3.98 3.97 3.96 3.94 3.93 3.92 3.91
2.28 2.27
1.68 1.68
1.28 1.25 1.24 1.23 1.22 1.19
-0.00
C2H5O
N
OC2H5
18
19.63
19.10
39.63
40.29
56.54
30.00
63.19
13.0 12.5 12.0 11.5 11.0 10.5 10.0 9.5 9.0 8.5 8.0 7.5 7.0 6.5 6.0 5.5 5.0 4.5 4.0 3.5 3.0 2.5 2.0 1.5 1.0 0.5 0.0
f1 (ppm)

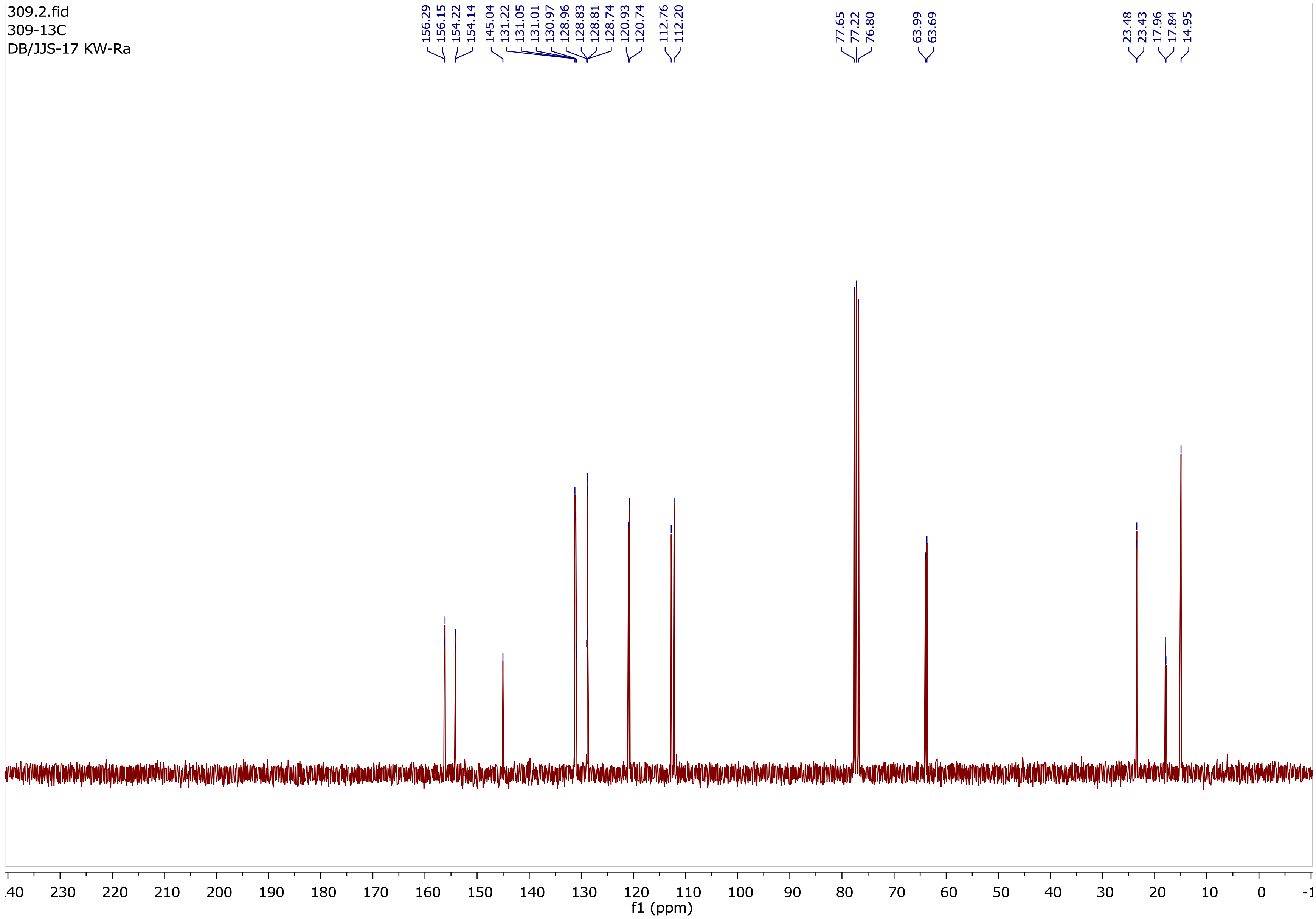
309.2.fid
309-13C
DB/JJS-17 KW-Ra
156.29
156.15
154.22
154.14
145.04
131.22
131.05
131.01
130.97
128.96
128.83
128.81
128.74
120.93
120.74
112.76
112.20
77.65
77.22
76.80
63.99
63.69
23.48
23.43
17.96
17.84
14.95
f1 (ppm)

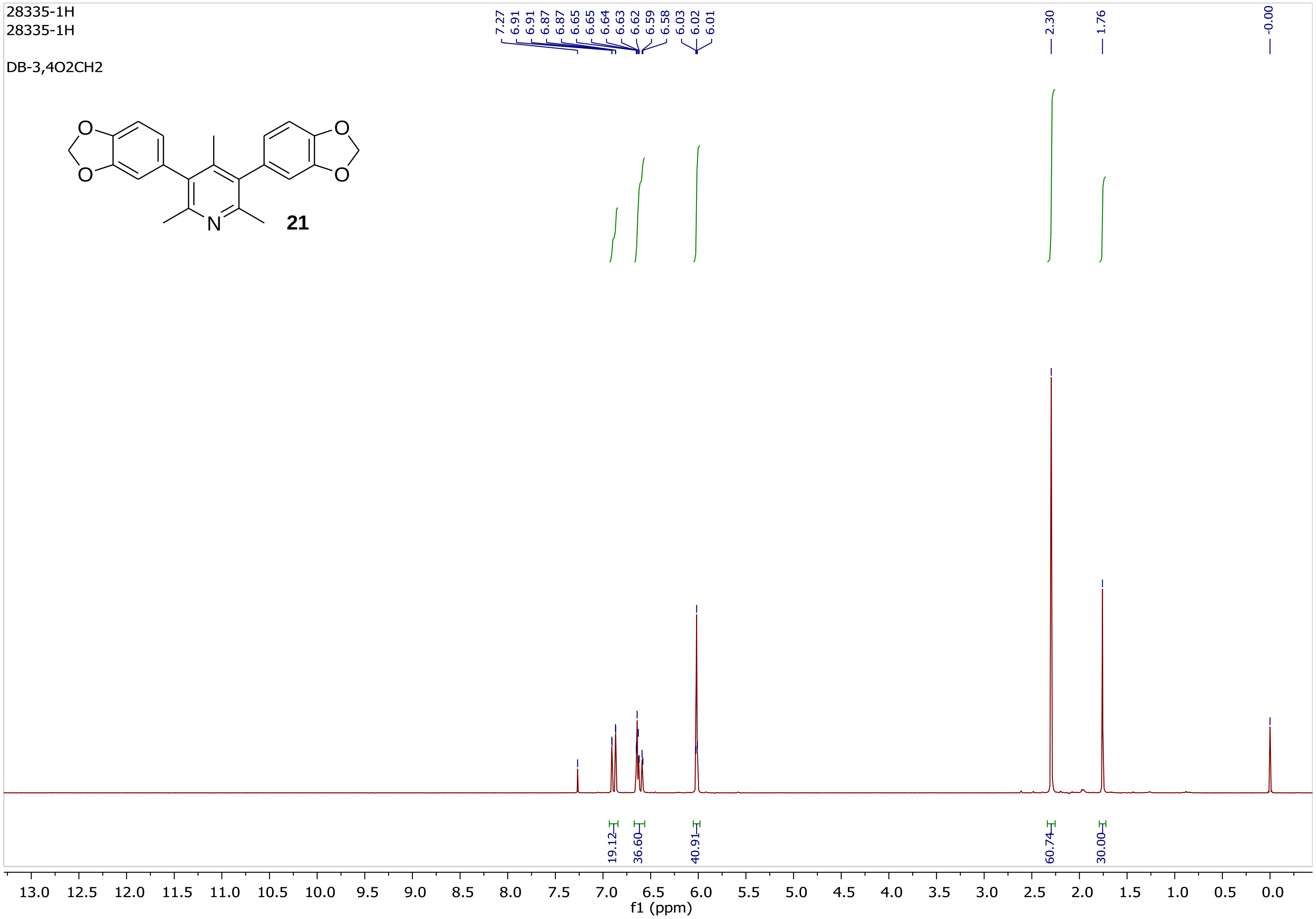
28335-1H
28335-1H
DB-3,4O2CH2
21
7.27
6.91
6.91
6.87
6.87
6.65
6.65
6.64
6.63
6.62
6.59
6.58
6.03
6.02
6.01
2.30
1.76
-0.00
19.12
36.60
40.91
60.74
30.00
13.0 12.5 12.0 11.5 11.0 10.5 10.0 9.5 9.0 8.5 8.0 7.5 7.0 6.5 6.0 5.5 5.0 4.5 4.0 3.5 3.0 2.5 2.0 1.5 1.0 0.5 0.0
f1 (ppm)

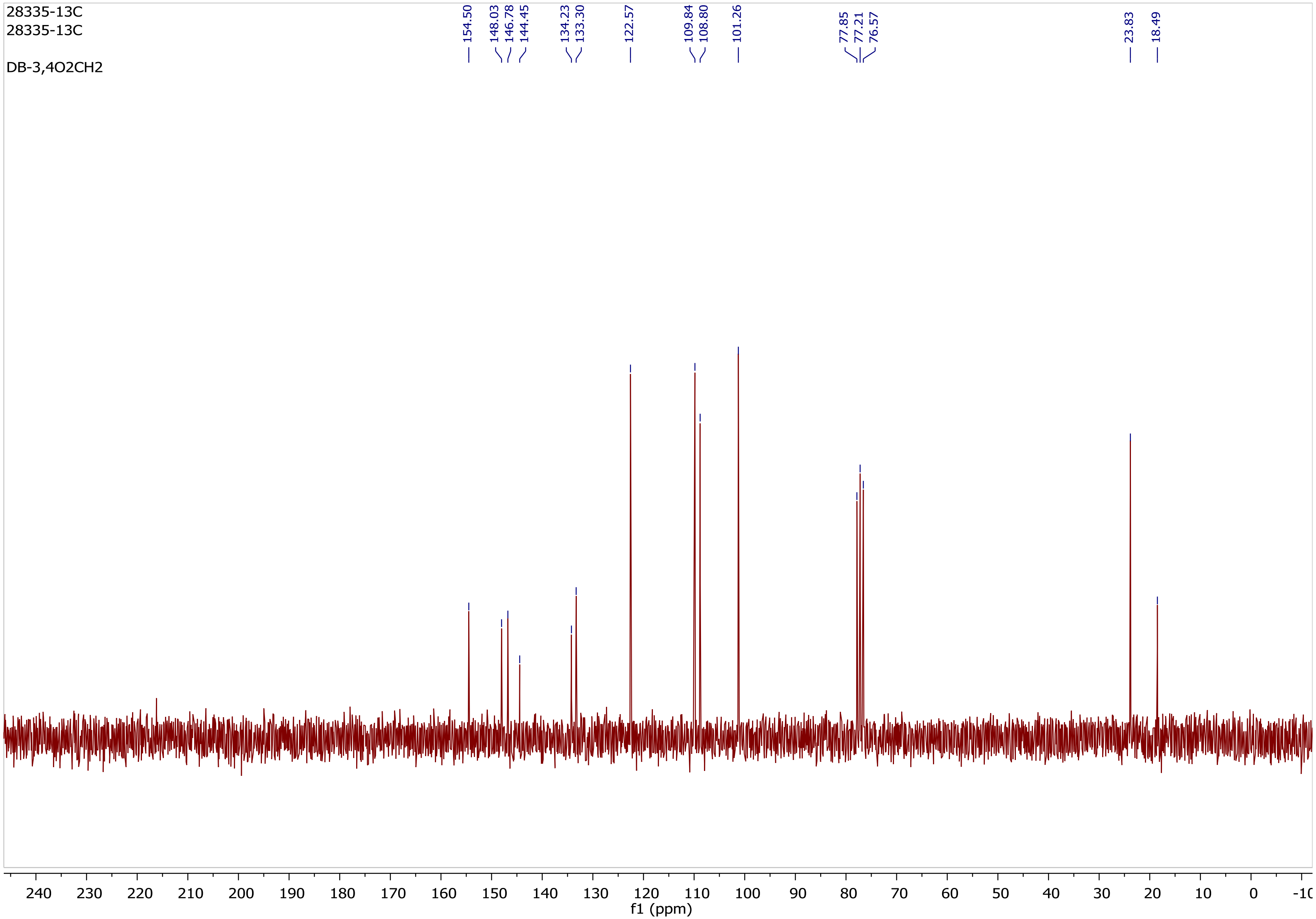
28335-13C
28335-13C
DB-3,4O2CH2
154.50
148.03
146.78
144.45
134.23
133.30
122.57
109.84
108.80
101.26
77.85
77.21
76.57
23.83
18.49
f1 (ppm)

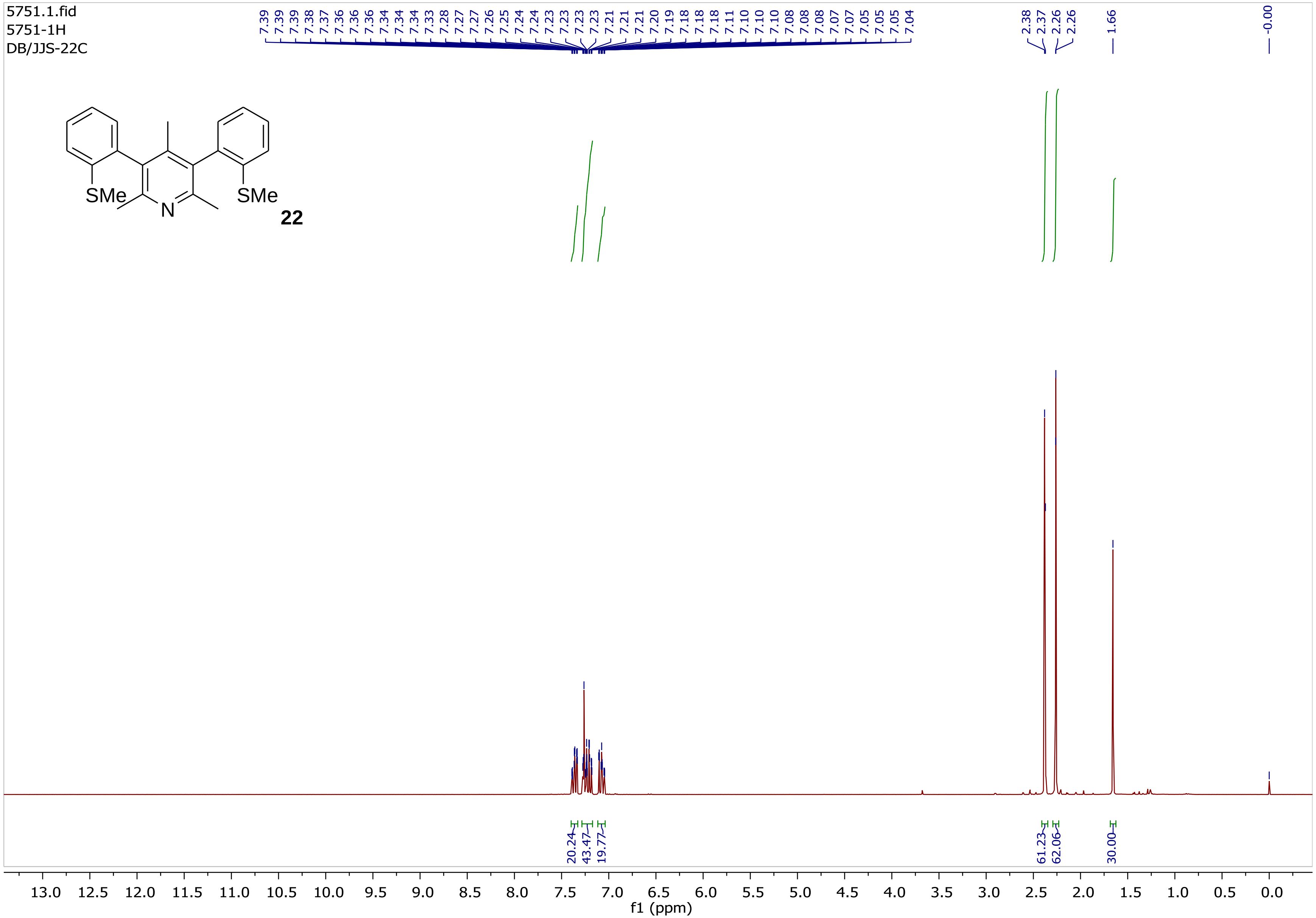
5751.1.fid
5751-1H
DB/JJS-22C
SMe
SMe
N
22
7.39 7.39 7.39 7.38 7.37 7.36 7.36 7.36 7.34 7.34 7.34 7.33 7.28 7.27 7.27 7.26 7.25 7.24 7.24 7.23 7.23 7.23 7.23 7.21 7.21 7.21 7.20 7.19 7.18 7.18 7.18 7.11 7.10 7.10 7.10 7.08 7.08 7.08 7.07 7.07 7.05 7.05 7.05 7.04
2.38 2.37 2.26 2.26
1.66
-0.00
20.24
43.47
19.77
61.23
62.06
30.00
13.0 12.5 12.0 11.5 11.0 10.5 10.0 9.5 9.0 8.5 8.0 7.5 7.0 6.5 6.0 5.5 5.0 4.5 4.0 3.5 3.0 2.5 2.0 1.5 1.0 0.5 0.0
f1 (ppm)

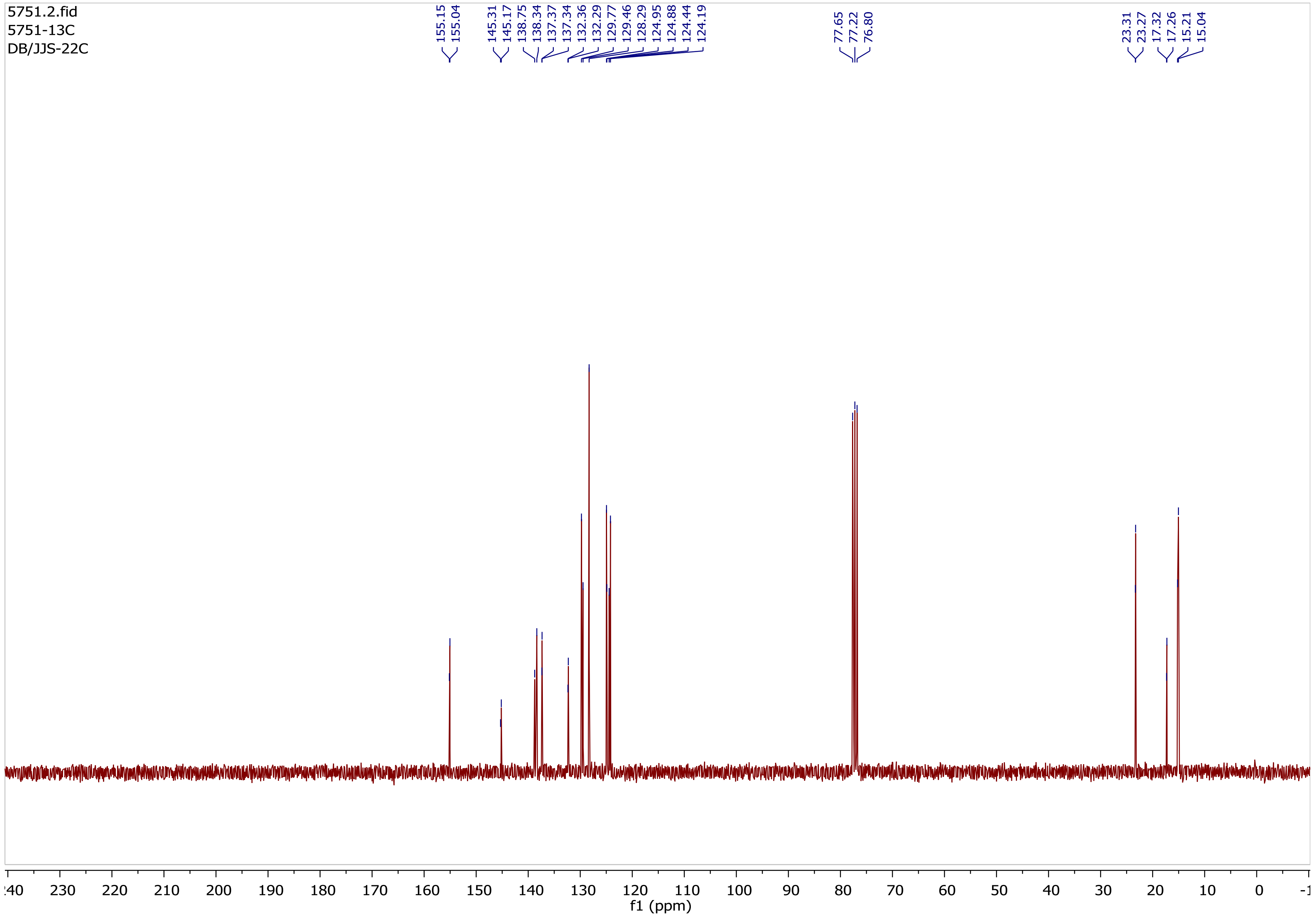
5751.2.fid
5751-13C
DB/JJS-22C
155.15
155.04
145.31
145.17
138.75
138.34
137.37
137.34
132.36
132.29
129.77
129.46
128.29
124.95
124.88
124.44
124.19
77.65
77.22
76.80
23.31
23.27
17.32
17.26
15.21
15.04
f1 (ppm)

50192-1H
50192-1H

DB/JJS-14

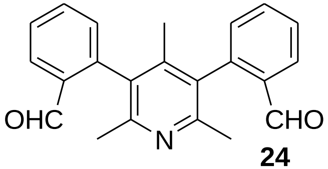

24

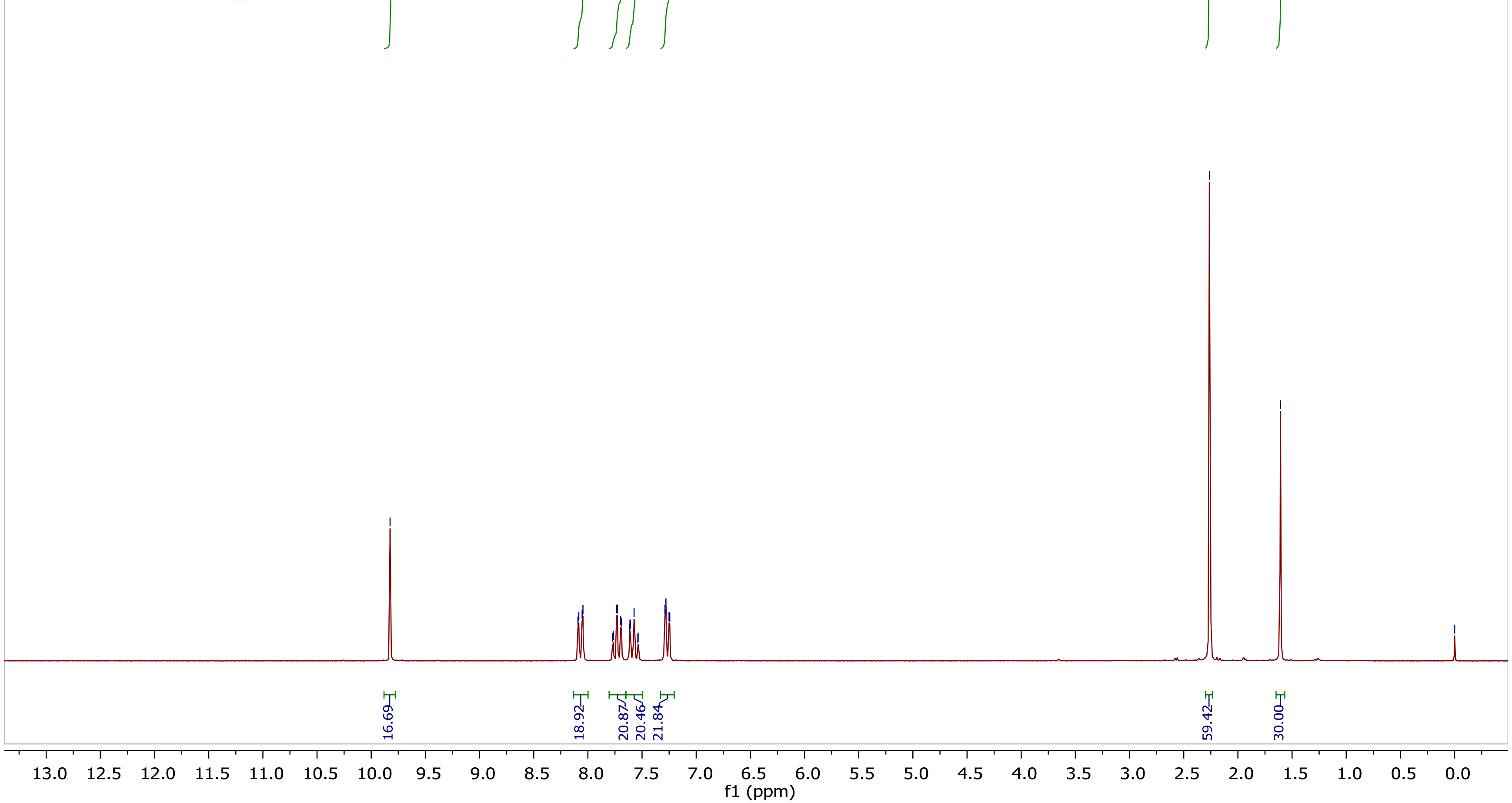

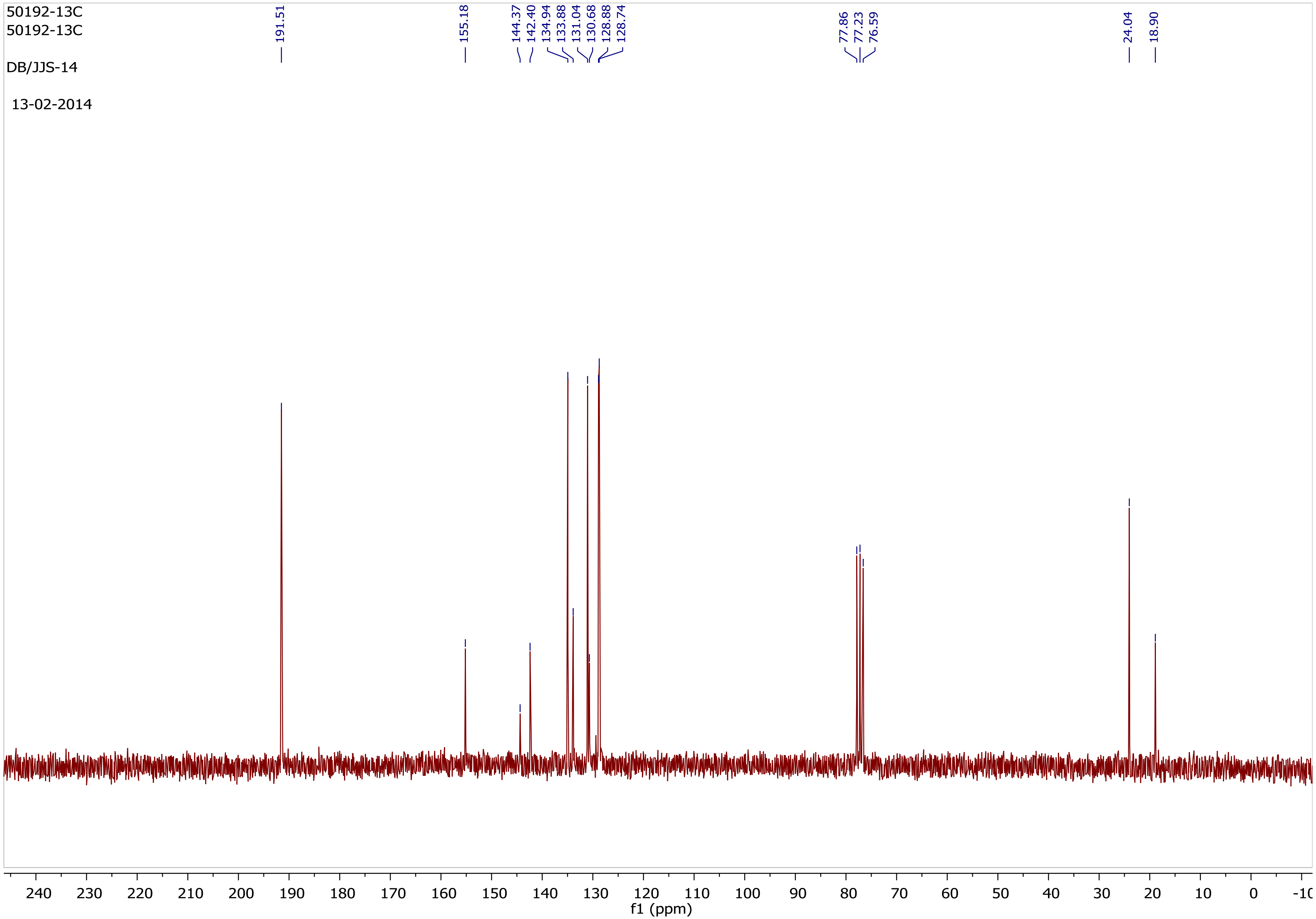
50192-13C
50192-13C
DB/JJS-14
13-02-2014
191.51
155.18
144.37
142.40
134.94
133.88
131.04
130.68
128.88
128.74
77.86
77.23
76.59
24.04
18.90
f1 (ppm)

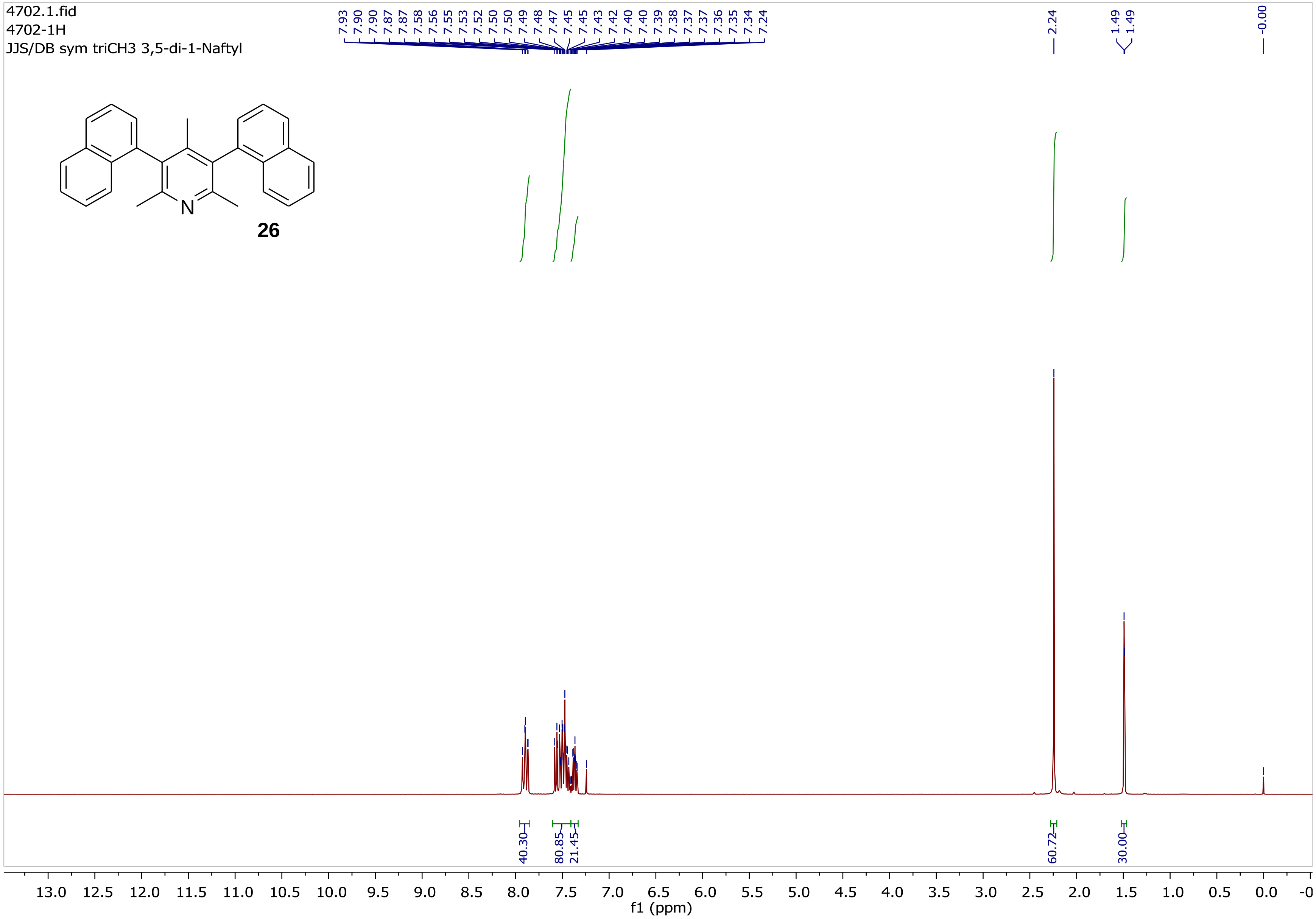
4702.1.fid
4702-1H
JJS/DB sym triCH3 3,5-di-1-Naftyl
N
26
7.93 7.90 7.90 7.87 7.58 7.56 7.55 7.53 7.52 7.50 7.50 7.49 7.48 7.47 7.45 7.45 7.43 7.42 7.40 7.40 7.39 7.38 7.37 7.37 7.36 7.35 7.34 7.24
2.24
1.49 1.49
-0.00
40.30
80.85
21.45
60.72
30.00
13.0 12.5 12.0 11.5 11.0 10.5 10.0 9.5 9.0 8.5 8.0 7.5 7.0 6.5 6.0 5.5 5.0 4.5 4.0 3.5 3.0 2.5 2.0 1.5 1.0 0.5 0.0 -0
f1 (ppm)

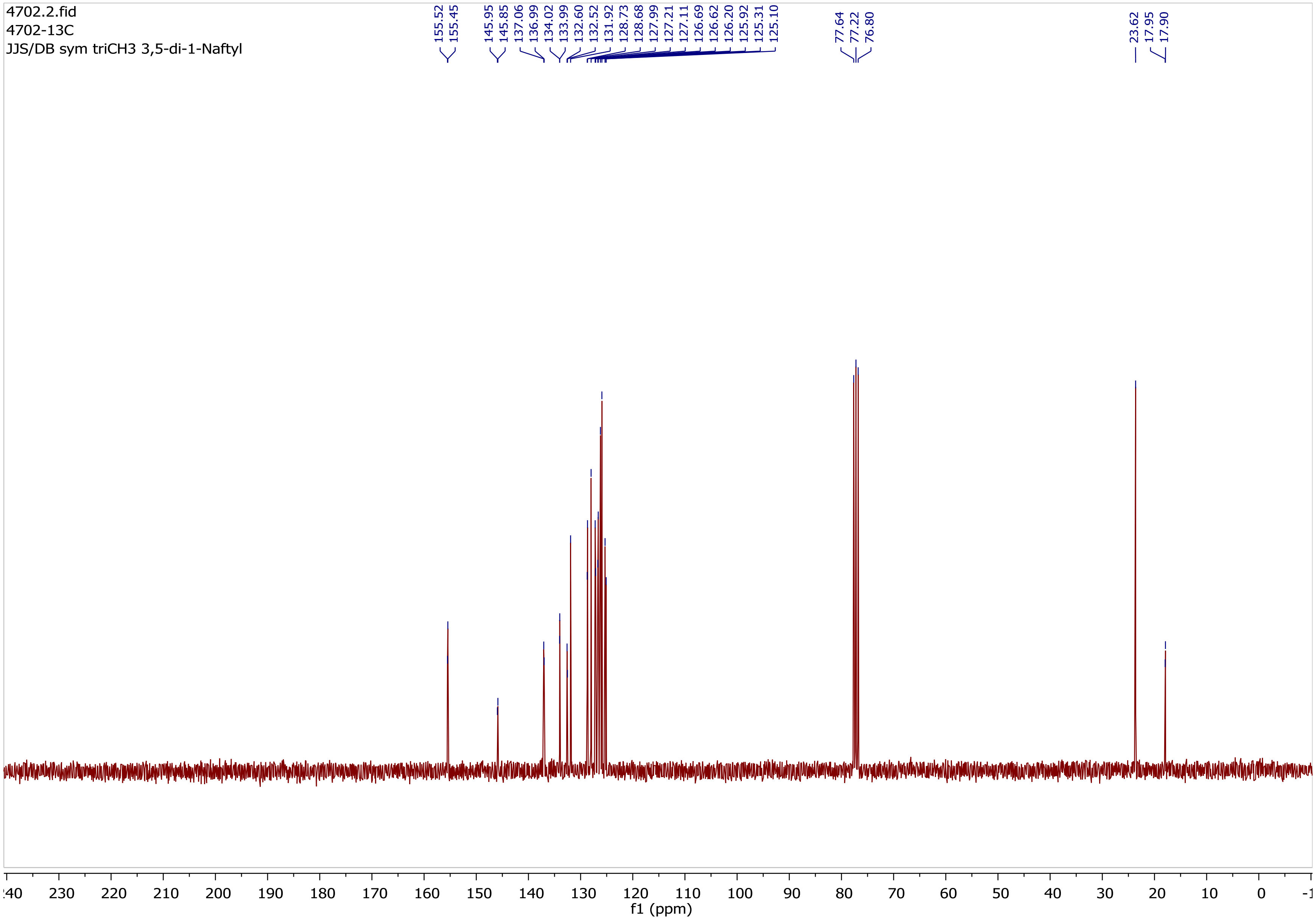

4702.2.fid
4702-13C
JJS/DB sym triCH3 3,5-di-1-Naftyl
155.52
155.45
145.95
145.85
137.06
136.99
134.02
133.99
132.60
132.52
131.92
128.73
128.68
127.99
127.21
127.11
126.69
126.62
126.20
125.92
125.31
125.10
77.64
77.22
76.80
23.62
17.95
17.90
240 230 220 210 200 190 180 170 160 150 140 130 120 110 100 90 80 70 60 50 40 30 20 10 0 -1
f1 (ppm)

176.1.fid
176-1H
DB/JJS-16B

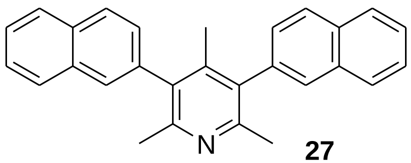

27

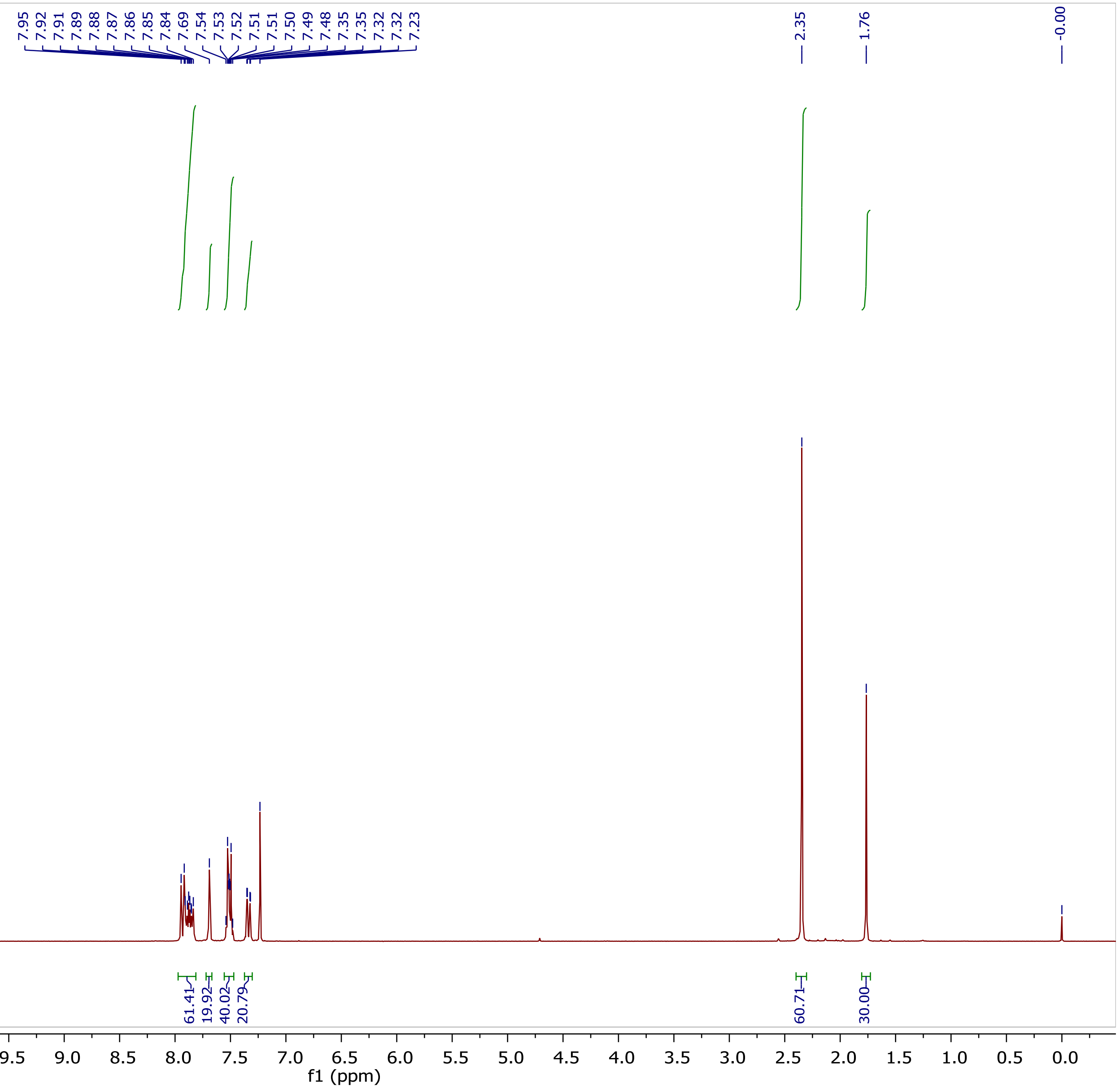

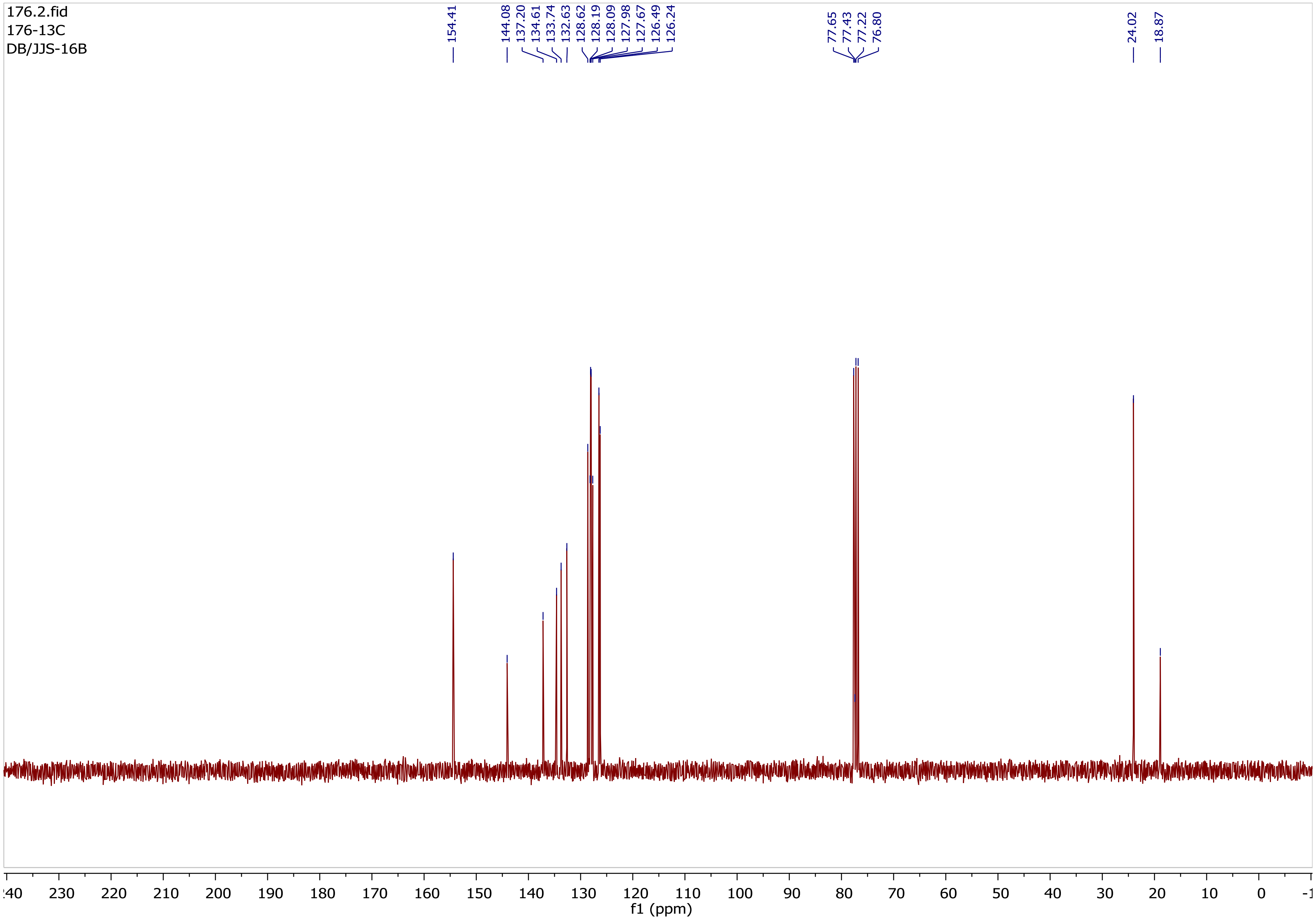
176.2.fid
176-13C
DB/JJS-16B
154.41
144.08
137.20
134.61
133.74
132.63
128.62
128.19
128.09
127.98
127.67
126.49
126.24
77.65
77.43
77.22
76.80
24.02
18.87
f1 (ppm)

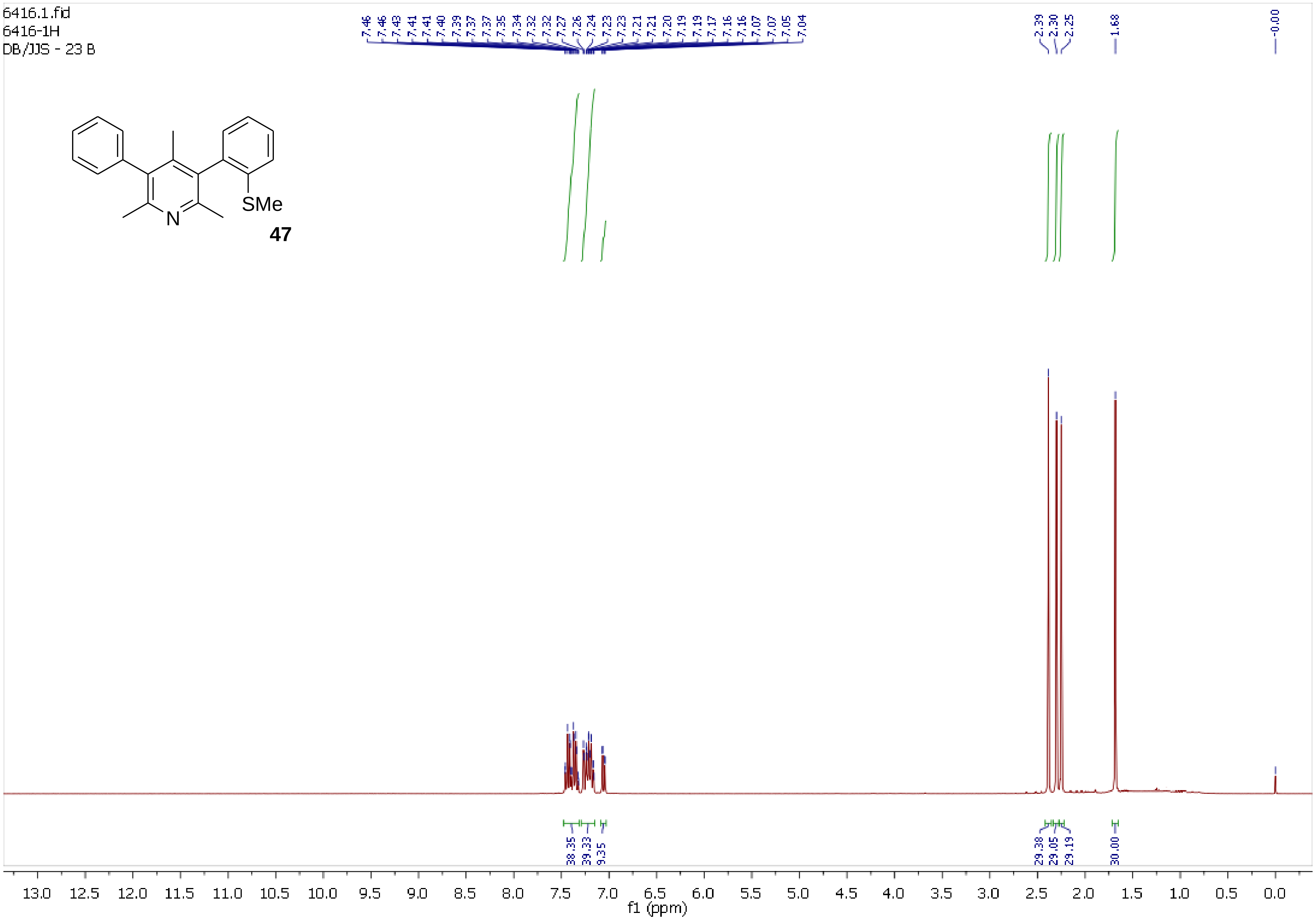
6416.1.fid
6416-1H
DB/JJS - 23 B
SMe
N
47
f1 (ppm)

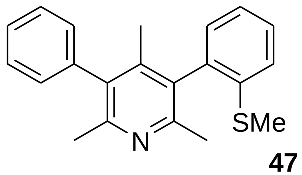

47

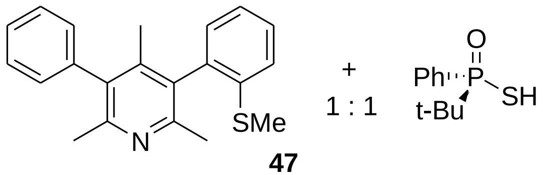

47

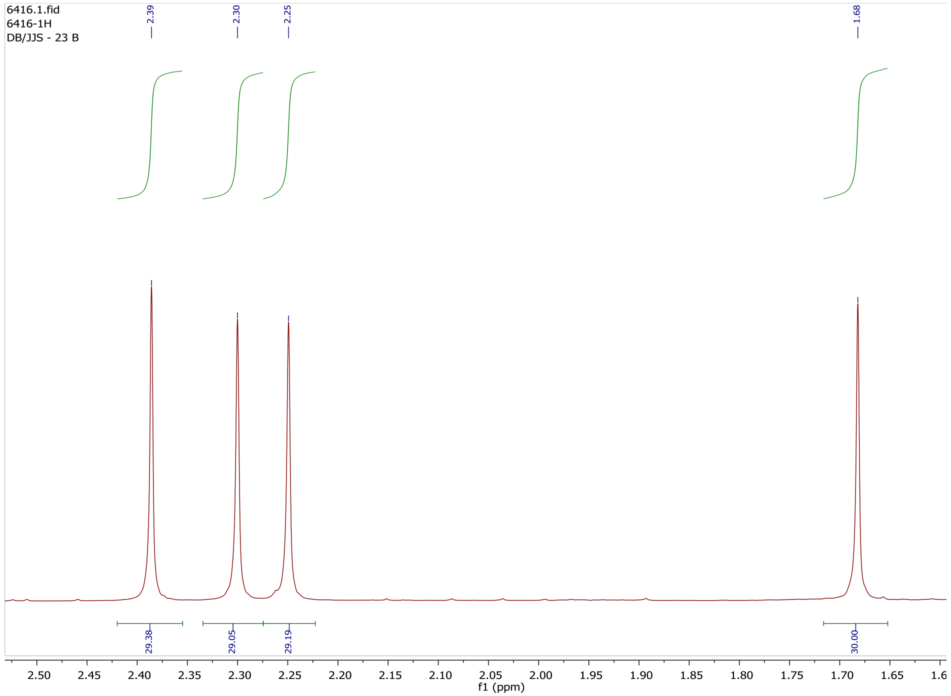
6416.1.fid
6416-1H
DB/JJS - 23 B
2.39
2.30
2.25
1.68
29.38
29.05
29.19
30.00
2.50 2.45 2.40 2.35 2.30 2.25 2.20 2.15 2.10 2.05 2.00 1.95 1.90 1.85 1.80 1.75 1.70 1.65 1.6
f1 (ppm)

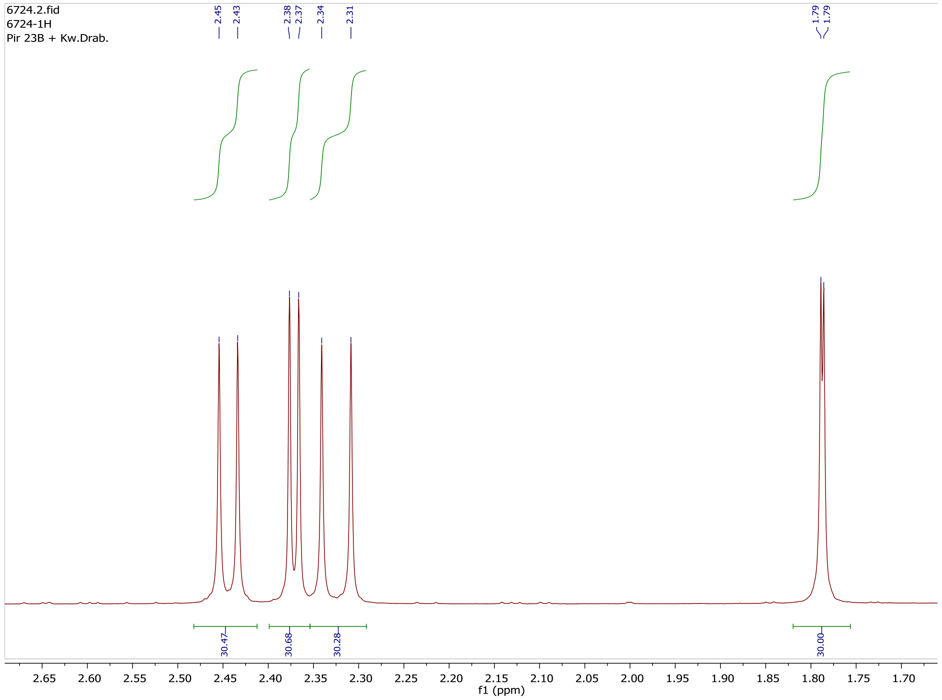
6724.2.fid
6724-1H
Pir 23B + Kw.Drab.
2.45
2.43
2.38
2.37
2.34
2.31
1.79
1.79
30.47
30.68
30.28
30.00
2.65 2.60 2.55 2.50 2.45 2.40 2.35 2.30 2.25 2.20 2.15 2.10 2.05 2.00 1.95 1.90 1.85 1.80 1.75 1.70
f1 (ppm)

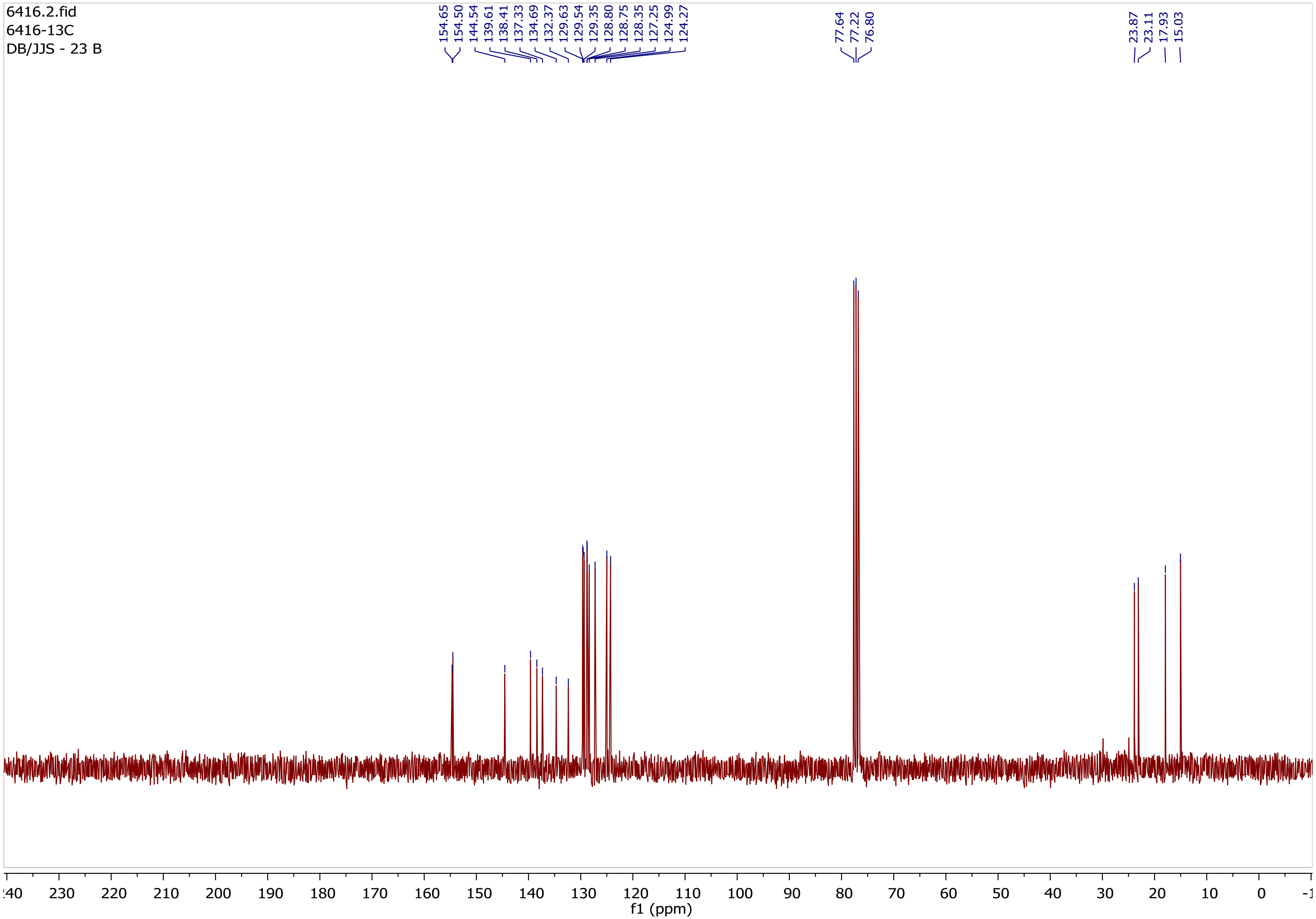
6416.2.fid
6416-13C
DB/JJS - 23 B
154.65
154.50
144.54
139.61
138.41
137.33
134.69
132.37
129.63
129.54
129.35
128.80
128.75
128.35
127.25
124.99
124.27
77.64
77.22
76.80
23.87
23.11
17.93
15.03
f1 (ppm)

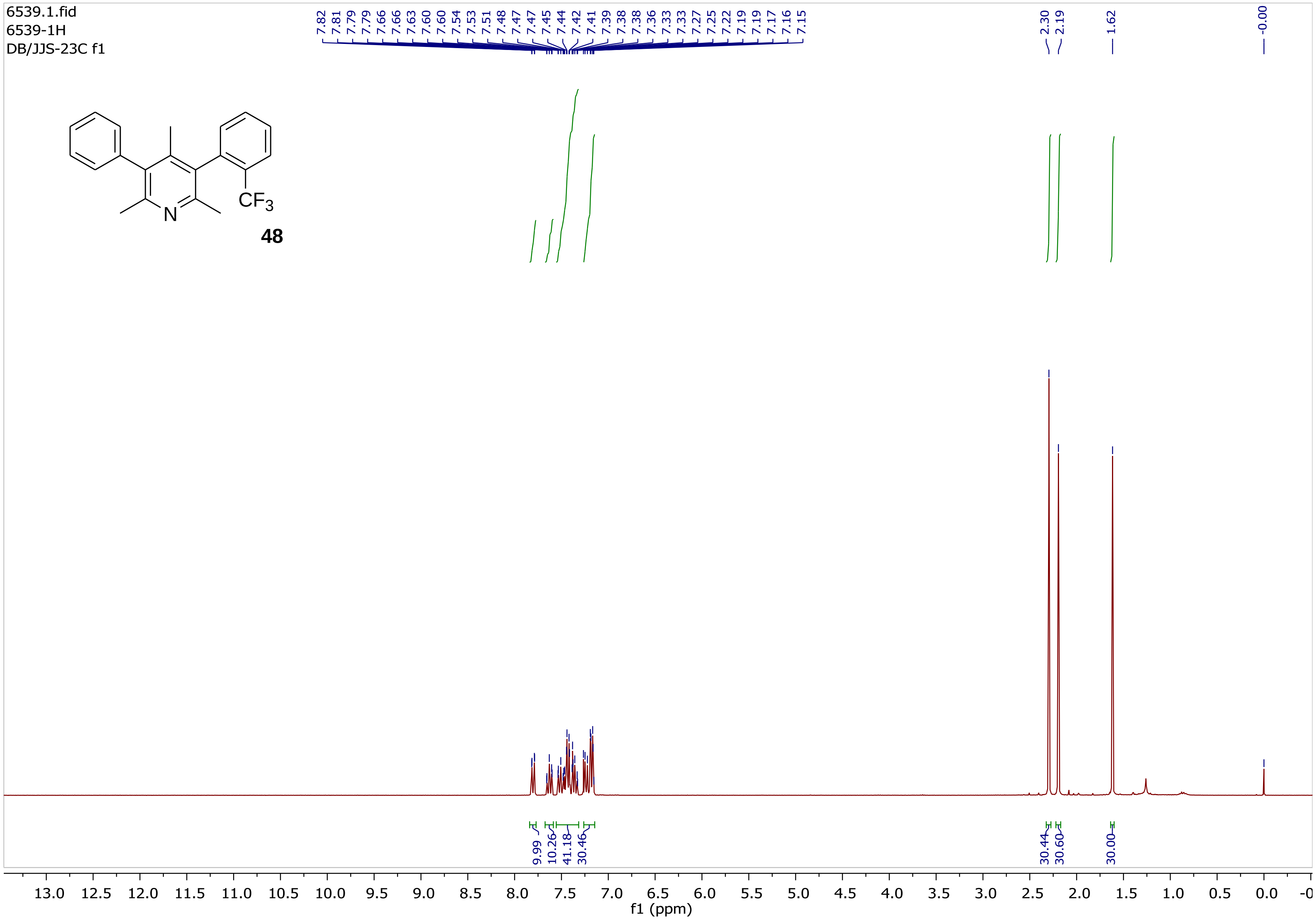
6539.1.fid
6539-1H
DB/JJS-23C f1
7.82 7.81 7.79 7.79 7.66 7.66 7.63 7.60 7.60 7.54 7.53 7.51 7.48 7.47 7.45 7.44 7.42 7.41 7.39 7.38 7.38 7.36 7.33 7.33 7.27 7.25 7.22 7.19 7.19 7.17 7.16 7.15
2.30
2.19
1.62
-0.00
CF3
N
48
9.99
10.26
41.18
30.46
30.44
30.60
30.00
13.0 12.5 12.0 11.5 11.0 10.5 10.0 9.5 9.0 8.5 8.0 7.5 7.0 6.5 6.0 5.5 5.0 4.5 4.0 3.5 3.0 2.5 2.0 1.5 1.0 0.5 0.0
f1 (ppm)

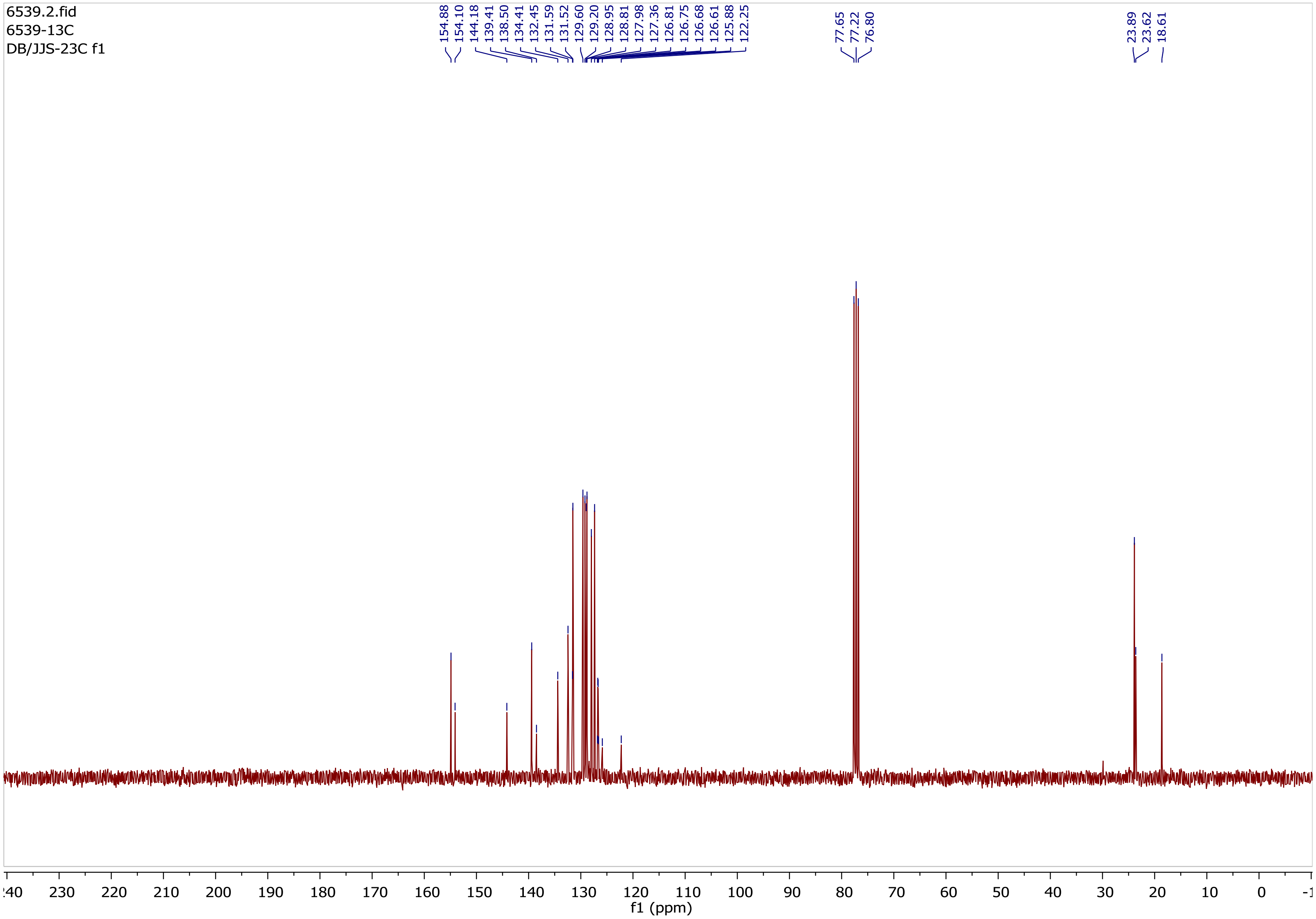
6539.2.fid
6539-13C
DB/JJS-23C f1
154.88
154.10
144.18
139.41
138.50
134.41
132.45
131.59
131.52
129.60
129.20
128.95
128.81
127.98
127.36
126.81
126.75
126.68
126.61
125.88
122.25
77.65
77.22
76.80
23.89
23.62
18.61
f1 (ppm)

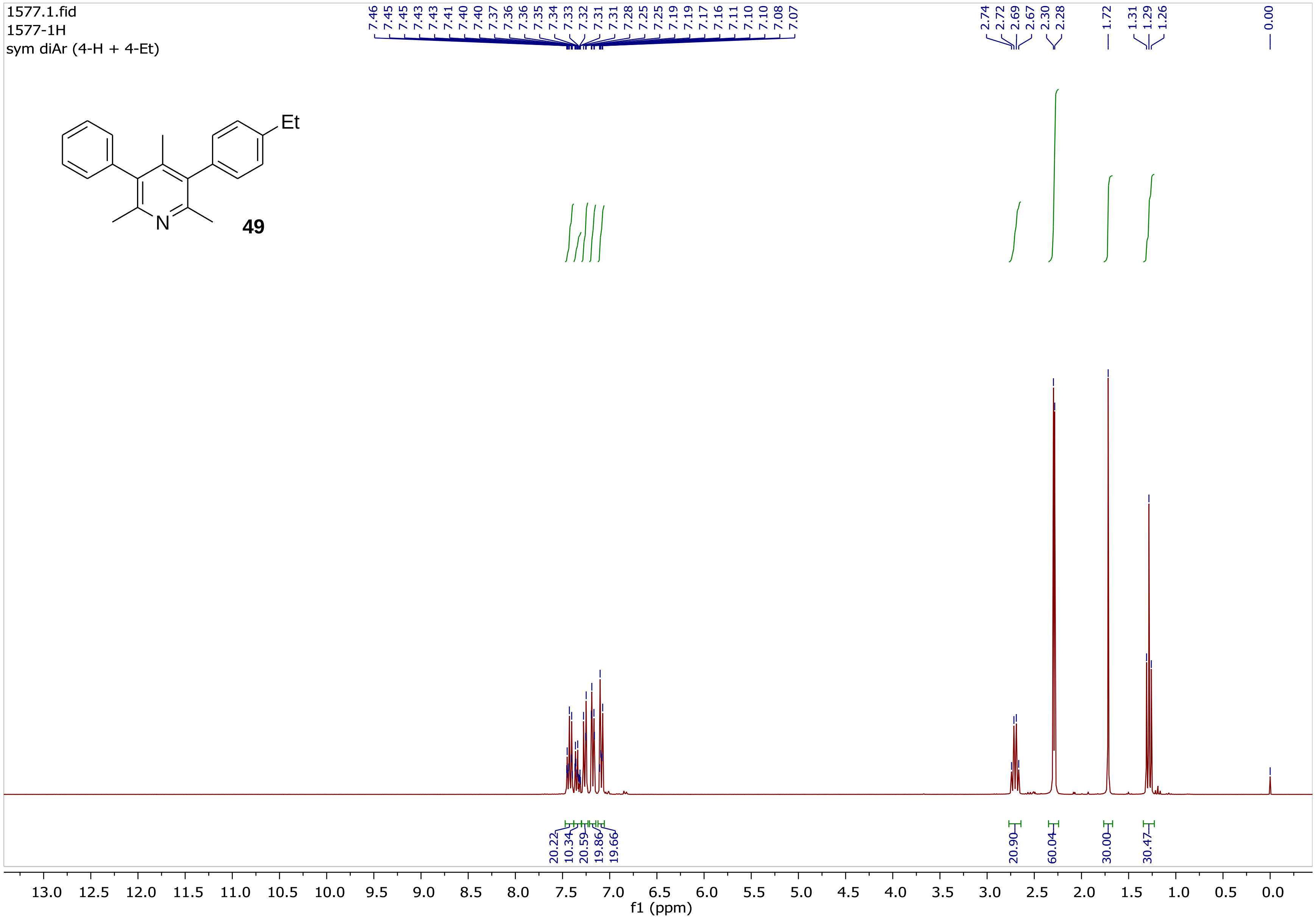
1577.1.fid
1577-1H
sym diAr (4-H + 4-Et)
Et
N
49
7.46 7.45 7.45 7.43 7.43 7.41 7.40 7.40 7.37 7.36 7.35 7.34 7.33 7.32 7.31 7.31 7.28 7.25 7.25 7.19 7.19 7.17 7.16 7.11 7.10 7.10 7.08 7.07
2.74 2.72 2.69 2.67 2.30 2.28
1.72
1.31 1.29 1.26
0.00
20.22 10.34 20.59 19.86 19.66
20.90 60.04 30.00 30.47
13.0 12.5 12.0 11.5 11.0 10.5 10.0 9.5 9.0 8.5 8.0 7.5 7.0 6.5 6.0 5.5 5.0 4.5 4.0 3.5 3.0 2.5 2.0 1.5 1.0 0.5 0.0
f1 (ppm)

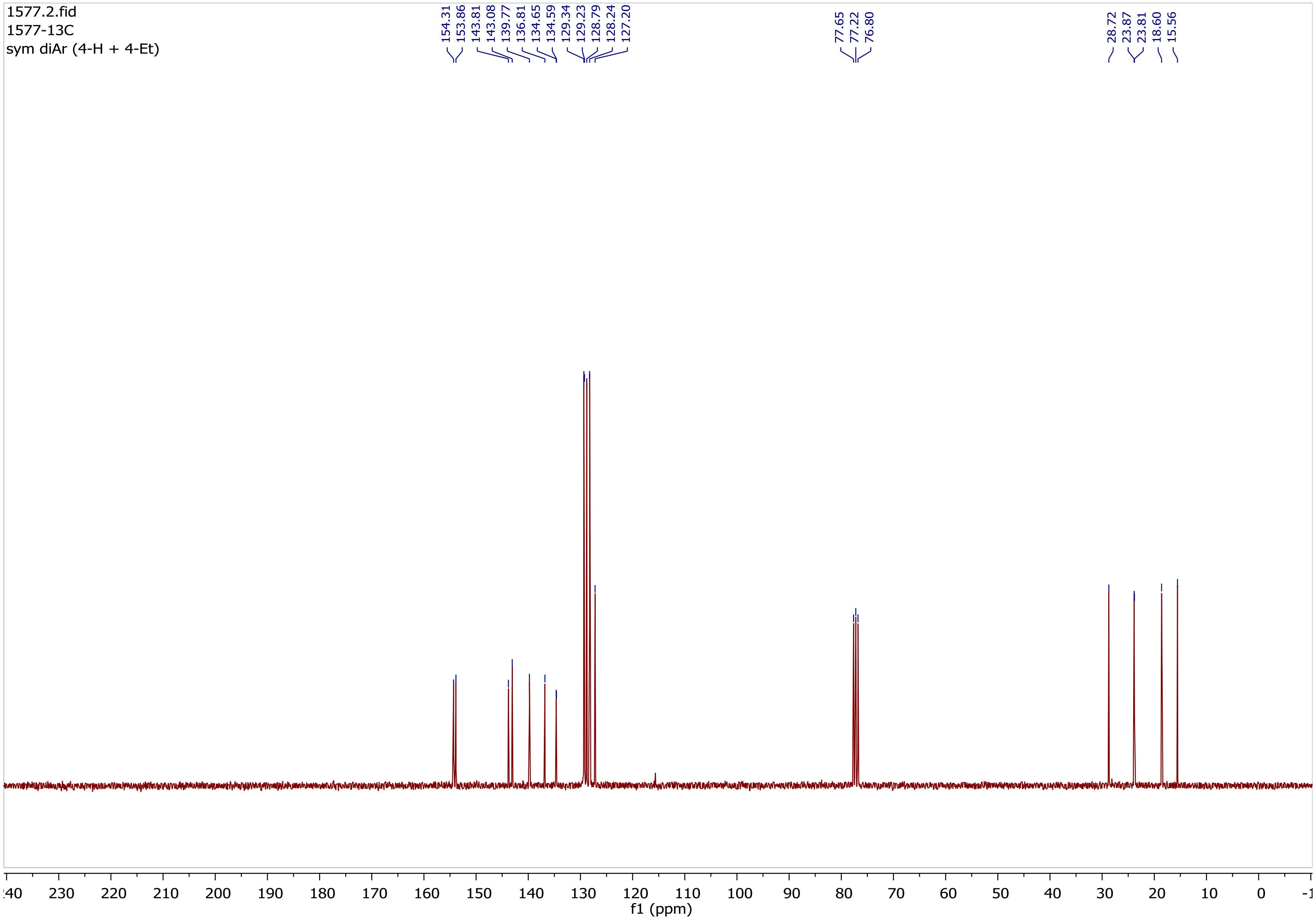
1577.2.fid
1577-13C
sym diAr (4-H + 4-Et)
154.31
153.86
143.81
143.08
139.77
136.81
134.65
134.59
129.34
129.23
128.79
128.24
127.20
77.65
77.22
76.80
28.72
23.87
23.81
18.60
15.56
f1 (ppm)

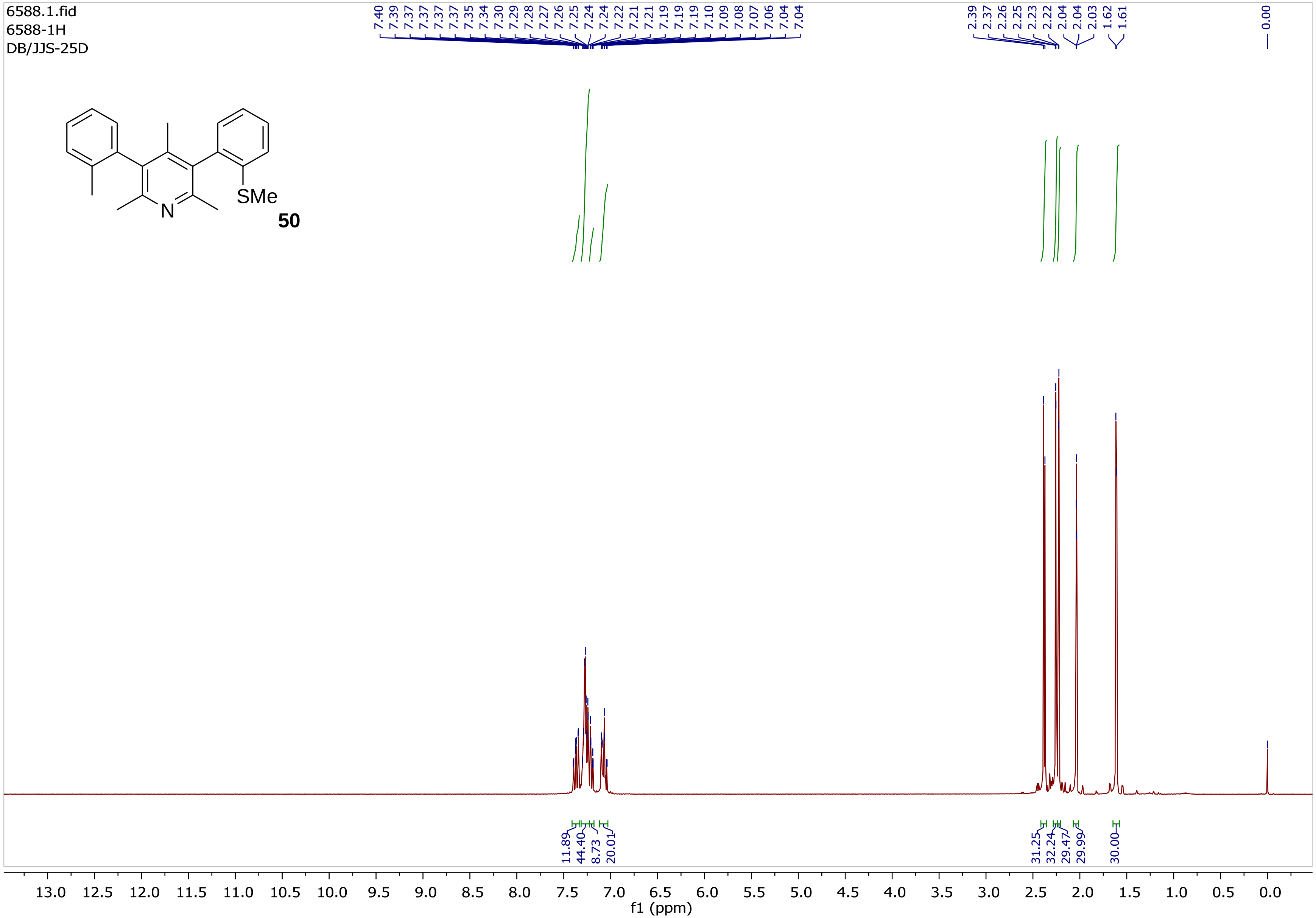
6588.1.fid
6588-1H
DB/JJS-25D
7.40 7.39 7.37 7.37 7.37 7.37 7.35 7.34 7.30 7.29 7.28 7.27 7.26 7.25 7.24 7.24 7.22 7.21 7.21 7.19 7.19 7.19 7.10 7.09 7.08 7.07 7.06 7.04 7.04
2.39 2.37 2.26 2.25 2.23 2.22 2.04 2.04 2.03 1.62 1.61
0.00
SMe
N
50
11.89 44.40 8.73 20.01
31.25 32.24 29.47 29.99 30.00
13.0 12.5 12.0 11.5 11.0 10.5 10.0 9.5 9.0 8.5 8.0 7.5 7.0 6.5 6.0 5.5 5.0 4.5 4.0 3.5 3.0 2.5 2.0 1.5 1.0 0.5 0.0
f1 (ppm)

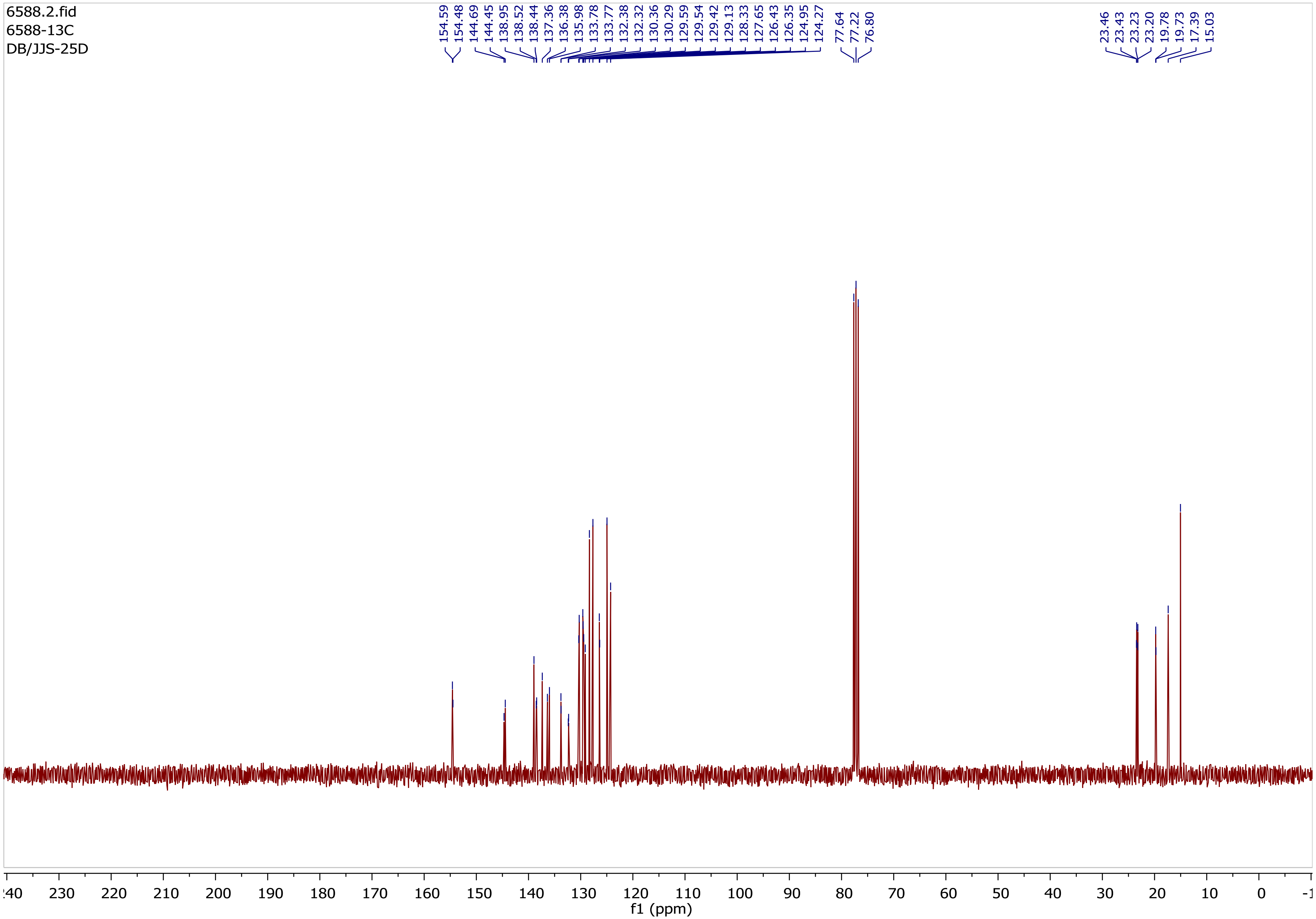
6588.2.fid
6588-13C
DB/JJS-25D
154.59
154.48
144.69
144.45
138.95
138.52
138.44
137.36
136.38
135.98
133.78
133.77
132.38
132.32
130.36
130.29
129.59
129.54
129.42
129.13
128.33
127.65
126.43
126.35
124.95
124.27
77.64
77.22
76.80
23.46
23.43
23.23
23.20
19.78
19.73
17.39
15.03
f1 (ppm)

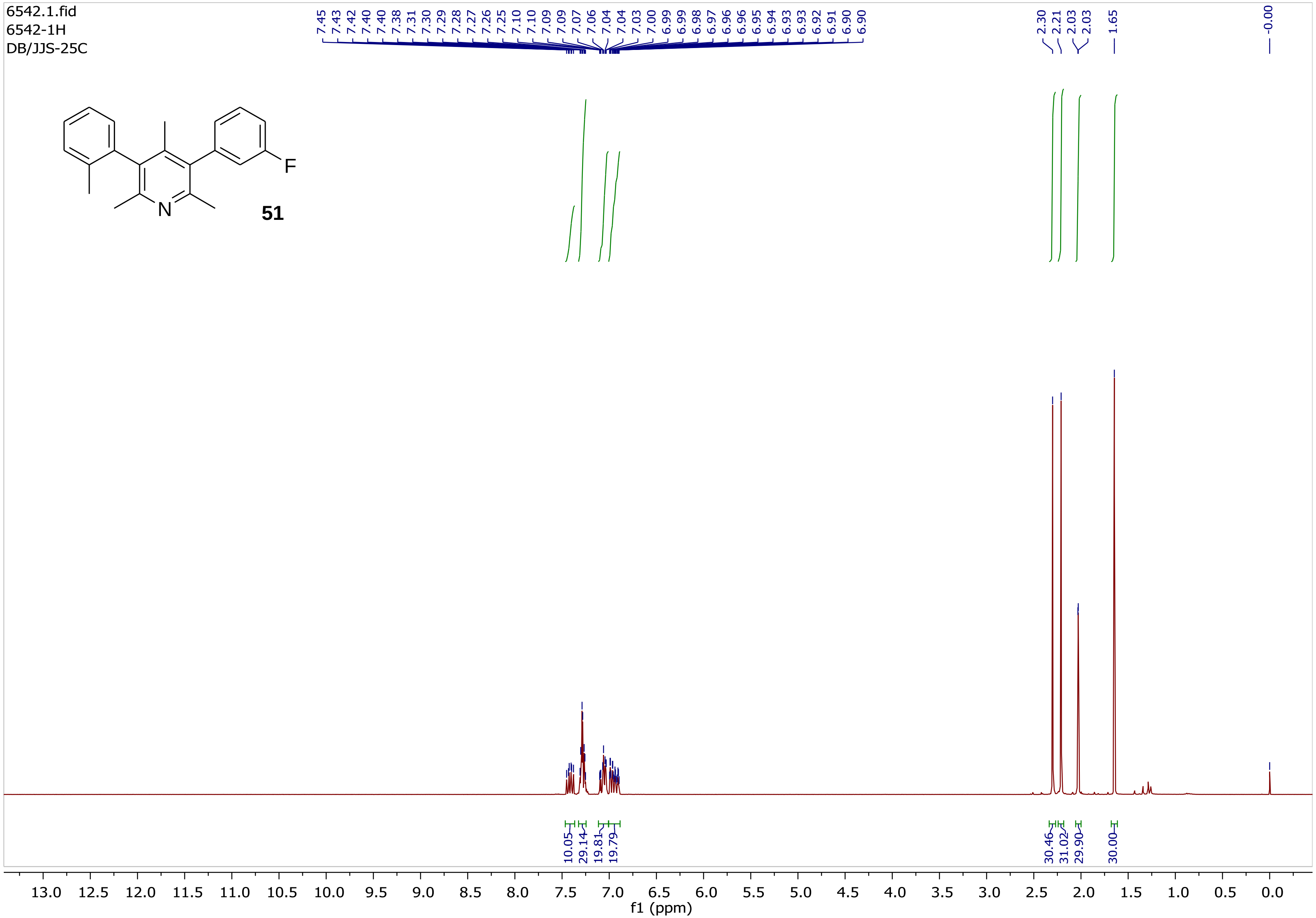
6542.1.fid
6542-1H
DB/JJS-25C
51
7.45 7.43 7.42 7.40 7.40 7.38 7.31 7.30 7.29 7.28 7.27 7.26 7.25 7.10 7.10 7.09 7.09 7.07 7.06 7.04 7.04 7.03 7.00 6.99 6.99 6.98 6.97 6.96 6.96 6.95 6.94 6.93 6.93 6.92 6.91 6.90 6.90
2.30 2.21 2.03 2.03 1.65
-0.00
10.05 29.14 19.81 19.79
30.46 31.02 29.90 30.00
f1 (ppm)

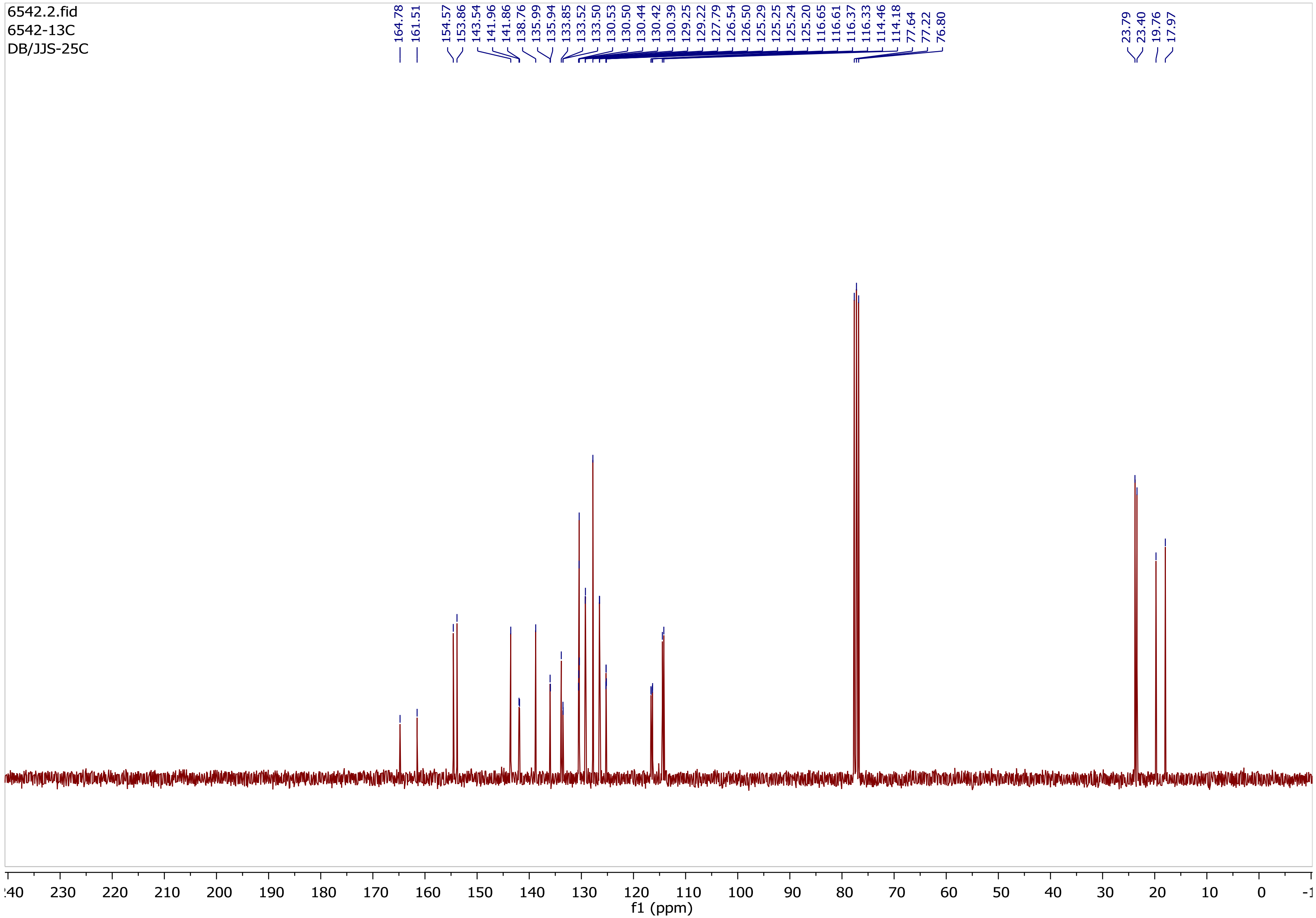
6542.2.fid
6542-13C
DB/JJS-25C
164.78
161.51
154.57
153.86
143.54
141.96
141.86
138.76
135.99
135.94
133.85
133.52
133.50
130.53
130.50
130.44
130.42
130.39
129.25
129.22
127.79
126.54
126.50
125.29
125.25
125.24
125.20
116.65
116.61
116.37
116.33
114.46
114.18
77.64
77.22
76.80
23.79
23.40
19.76
17.97
f1 (ppm)

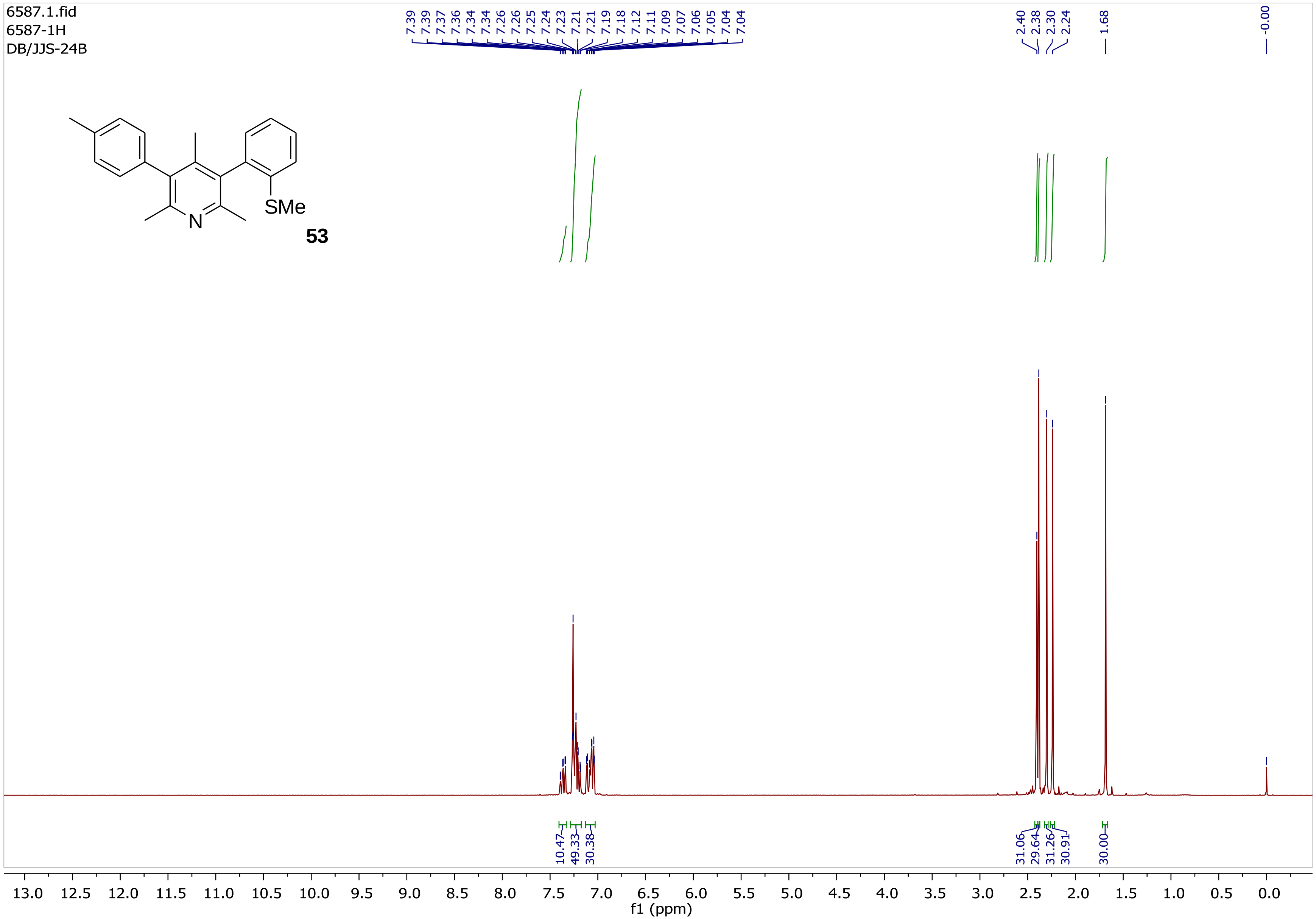
6587.1.fid
6587-1H
DB/JJS-24B
7.39
7.39
7.37
7.36
7.34
7.34
7.26
7.26
7.25
7.24
7.23
7.21
7.21
7.19
7.18
7.12
7.11
7.09
7.07
7.06
7.05
7.04
7.04
2.40
2.38
2.30
2.24
1.68
-0.00
SMe
N
53
10.47
49.33
30.38
31.06
29.64
31.26
30.91
30.00
13.0 12.5 12.0 11.5 11.0 10.5 10.0 9.5 9.0 8.5 8.0 7.5 7.0 6.5 6.0 5.5 5.0 4.5 4.0 3.5 3.0 2.5 2.0 1.5 1.0 0.5 0.0
f1 (ppm)

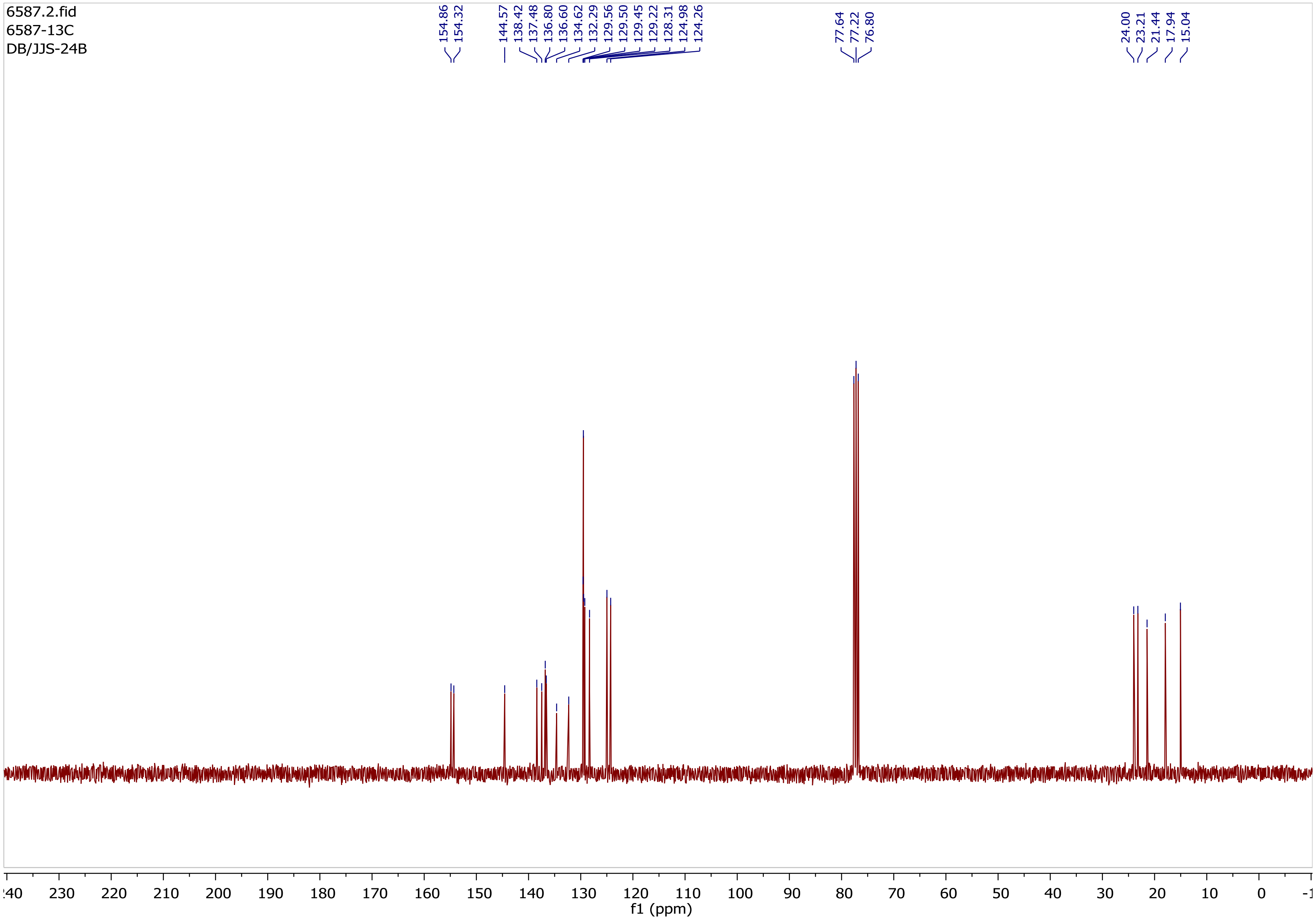
6587.2.fid
6587-13C
DB/JJS-24B
154.86
154.32
144.57
138.42
137.48
136.80
136.60
134.62
132.29
129.56
129.50
129.45
129.22
128.31
124.98
124.26
77.64
77.22
76.80
24.00
23.21
21.44
17.94
15.04
f1 (ppm)

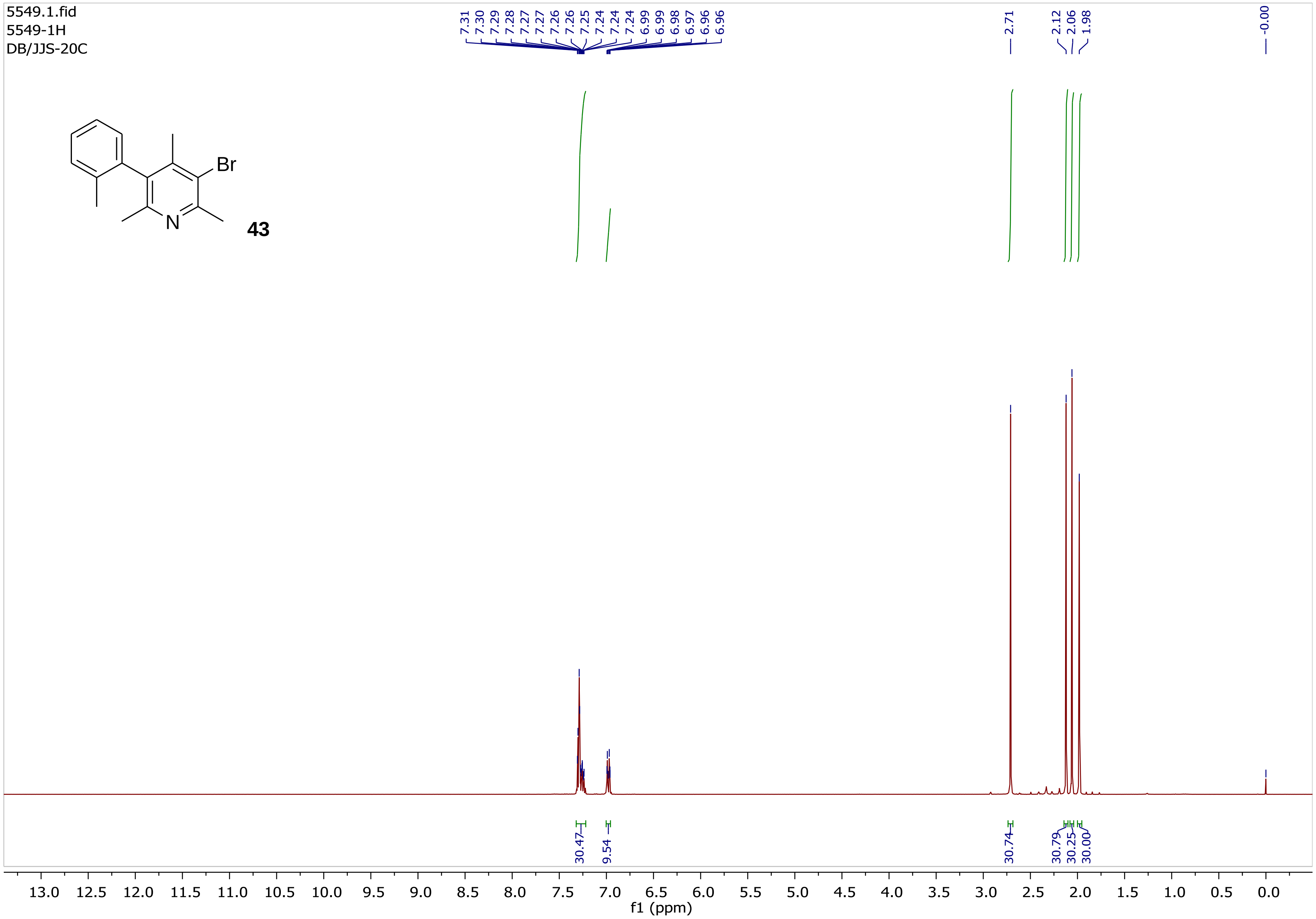
5549.1.fid
5549-1H
DB/JJS-20C
7.31 7.30 7.29 7.28 7.27 7.27 7.26 7.26 7.25 7.24 7.24 7.24 6.99 6.99 6.98 6.97 6.96 6.96
2.71
2.12 2.06 1.98
-0.00
Br
N
43
30.47 9.54 30.74 30.79 30.25 30.00
13.0 12.5 12.0 11.5 11.0 10.5 10.0 9.5 9.0 8.5 8.0 7.5 7.0 6.5 6.0 5.5 5.0 4.5 4.0 3.5 3.0 2.5 2.0 1.5 1.0 0.5 0.0
f1 (ppm)

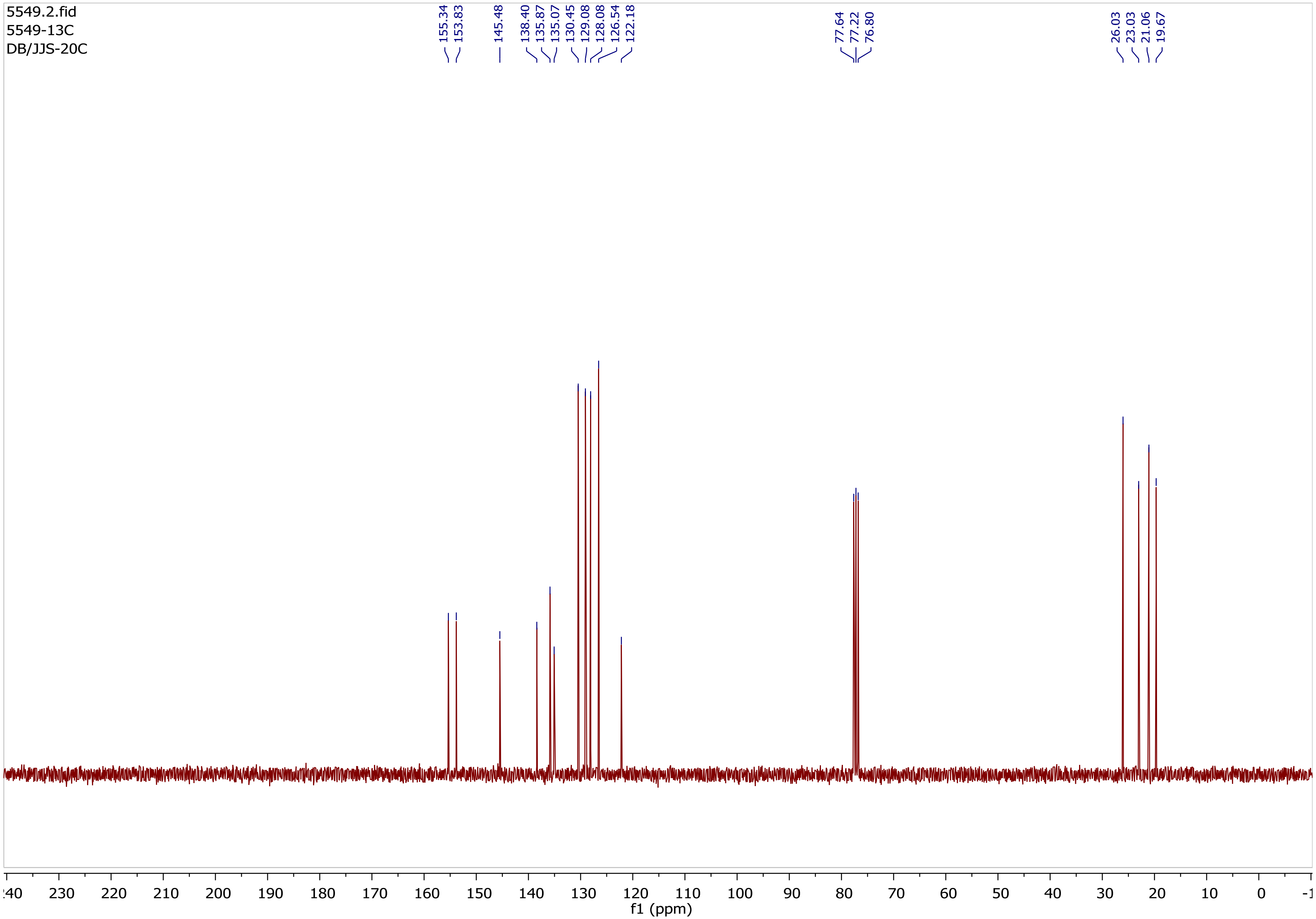
5549.2.fid
5549-13C
DB/JJS-20C
155.34
153.83
145.48
138.40
135.87
135.07
130.45
129.08
128.08
126.54
122.18
77.64
77.22
76.80
26.03
23.03
21.06
19.67
f1 (ppm)

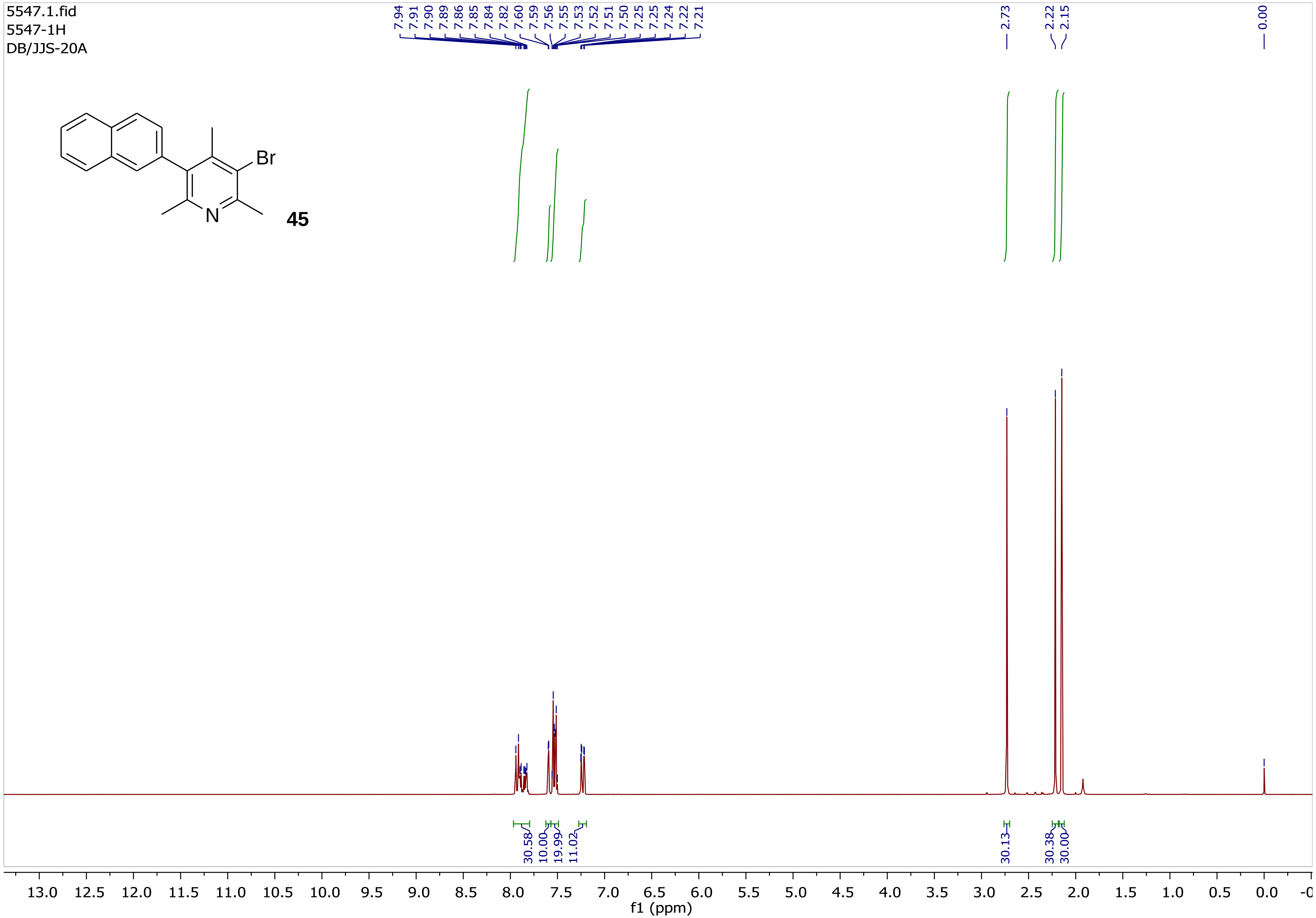
5547.1.fid
5547-1H
DB/JJS-20A
Br
N
45
7.94
7.91
7.90
7.89
7.86
7.85
7.84
7.82
7.60
7.59
7.56
7.55
7.53
7.52
7.51
7.50
7.25
7.25
7.24
7.22
7.21
2.73
2.22
2.15
0.00
30.58
10.00
19.99
11.02
30.13
30.38
30.00
13.0 12.5 12.0 11.5 11.0 10.5 10.0 9.5 9.0 8.5 8.0 7.5 7.0 6.5 6.0 5.5 5.0 4.5 4.0 3.5 3.0 2.5 2.0 1.5 1.0 0.5 0.0
f1 (ppm)

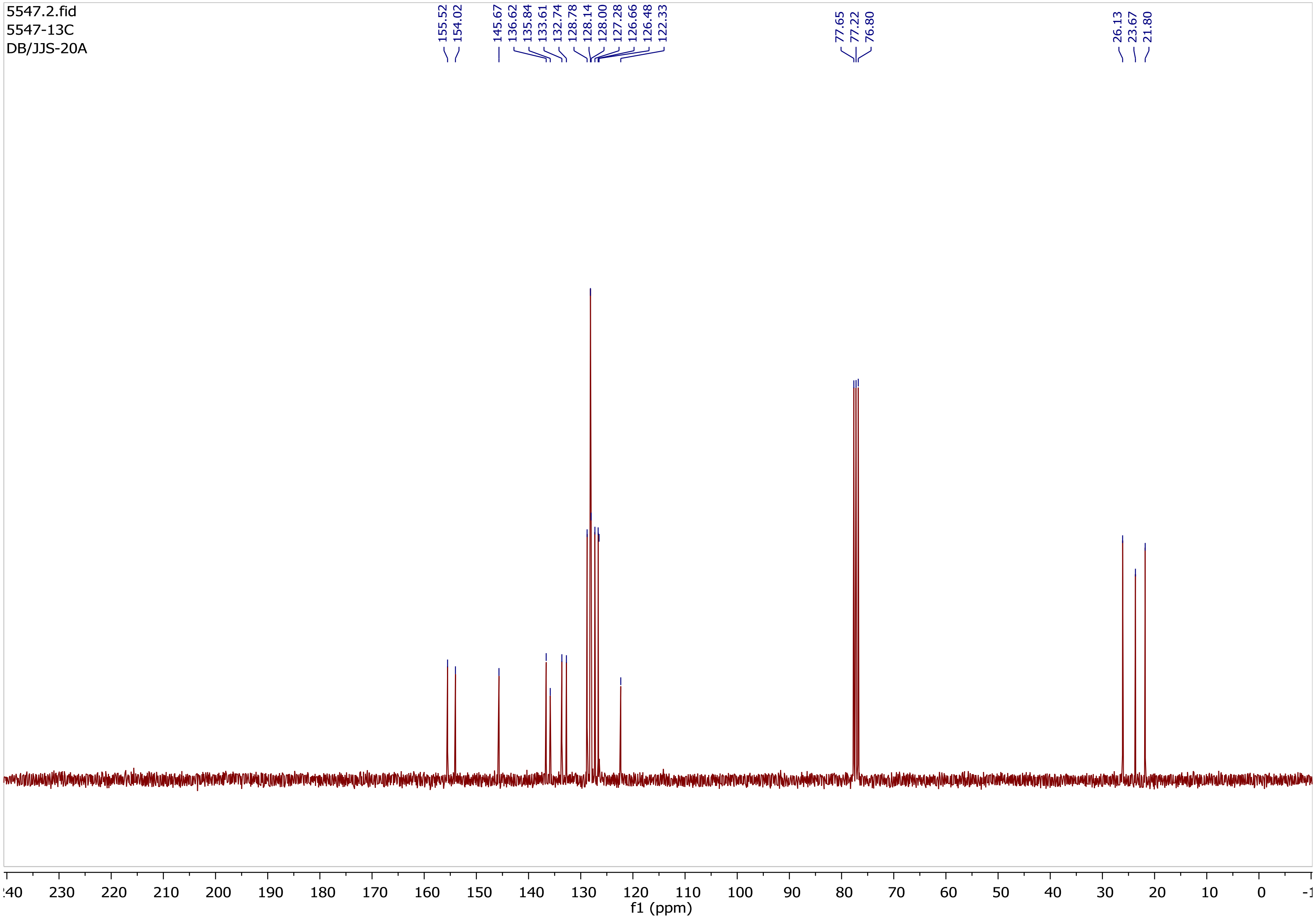
5547.2.fid
5547-13C
DB/JJS-20A
155.52
154.02
145.67
136.62
135.84
133.61
132.74
128.78
128.14
128.00
127.28
126.66
126.48
122.33
77.65
77.22
76.80
26.13
23.67
21.80
f1 (ppm)

6658.1.fid
6658-1H
DB/JJS-26 B

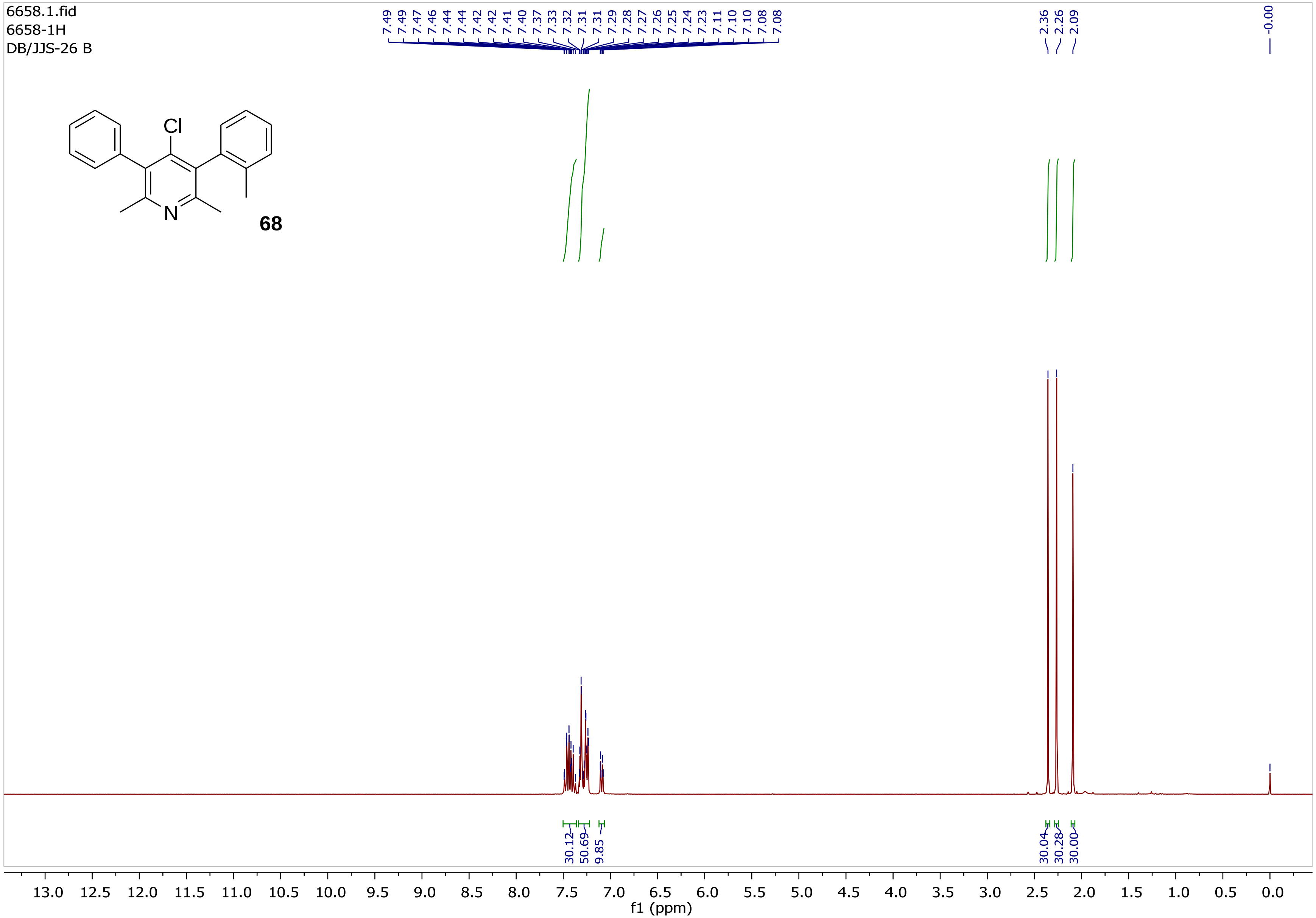

68

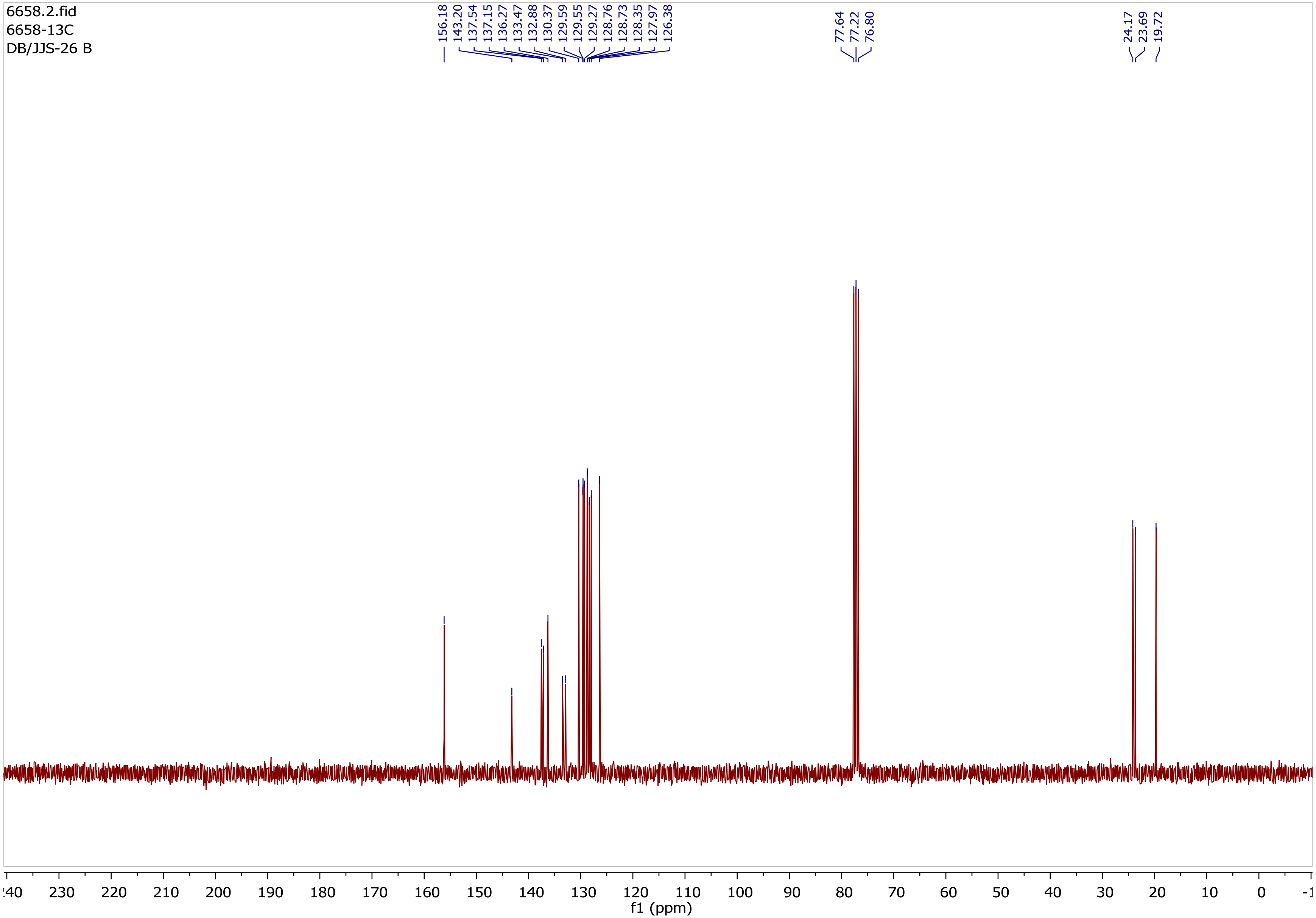
6658.2.fid
6658-13C
DB/JJS-26 B
156.18
143.20
137.54
137.15
136.27
133.47
132.88
130.37
129.59
129.55
129.27
128.76
128.73
128.35
127.97
126.38
77.64
77.22
76.80
24.17
23.69
19.72
f1 (ppm)

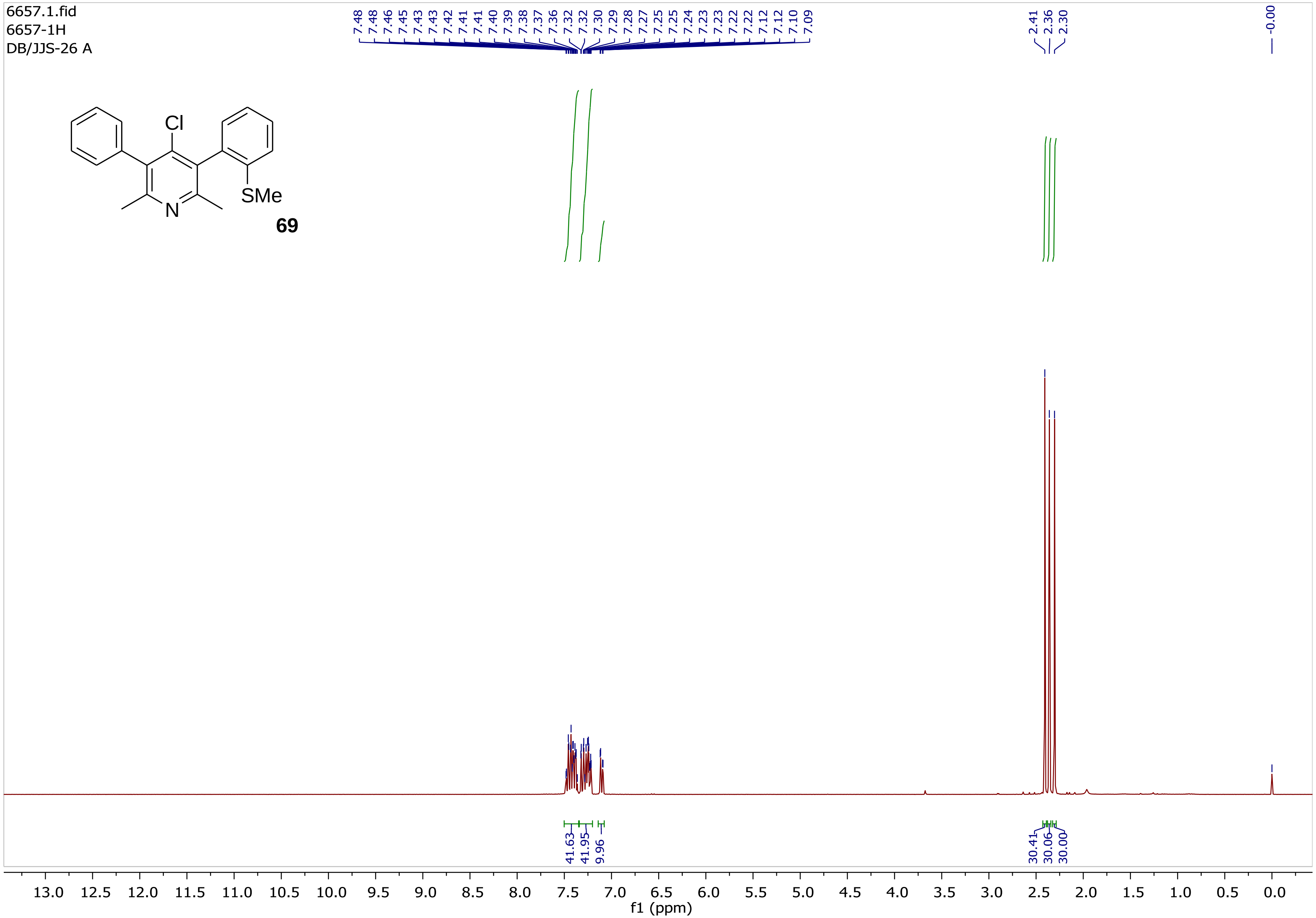
6657.1.fid
6657-1H
DB/JJS-26 A
Cl
N
SMe
69
7.48 7.48 7.46 7.45 7.43 7.43 7.42 7.41 7.41 7.40 7.39 7.38 7.37 7.36 7.32 7.32 7.30 7.29 7.28 7.27 7.25 7.25 7.24 7.23 7.23 7.22 7.22 7.12 7.10 7.09
2.41 2.36 2.30
-0.00
41.63 41.95 9.96
30.41 30.06 30.00
13.0 12.5 12.0 11.5 11.0 10.5 10.0 9.5 9.0 8.5 8.0 7.5 7.0 6.5 6.0 5.5 5.0 4.5 4.0 3.5 3.0 2.5 2.0 1.5 1.0 0.5 0.0
f1 (ppm)

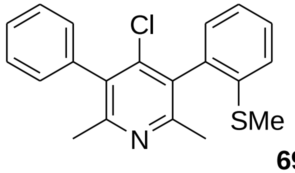
Cl
SMe
N

69

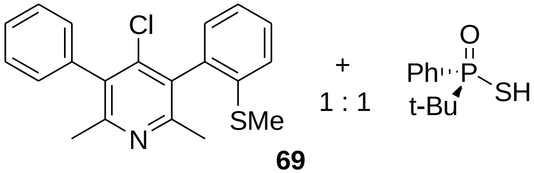
Cl
SMe
N
+
1 : 1
O
Ph
P
SH
t-Bu

69

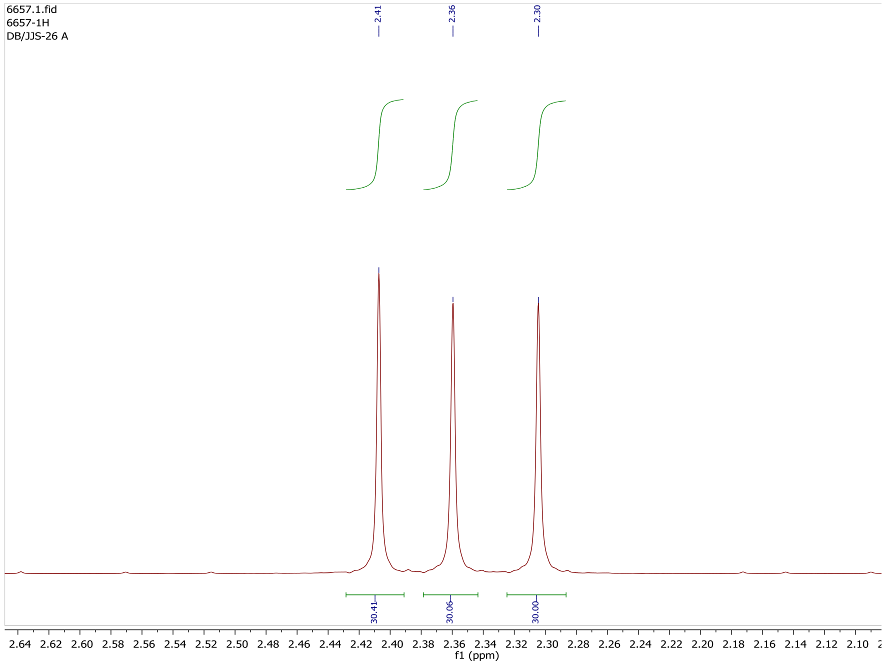
6657.1.fid
6657-1H
DB/JJS-26 A
2.41
2.36
2.30
30.41
30.06
30.00
2.64 2.62 2.60 2.58 2.56 2.54 2.52 2.50 2.48 2.46 2.44 2.42 2.40 2.38 2.36 2.34 2.32 2.30 2.28 2.26 2.24 2.22 2.20 2.18 2.16 2.14 2.12 2.10
f1 (ppm)

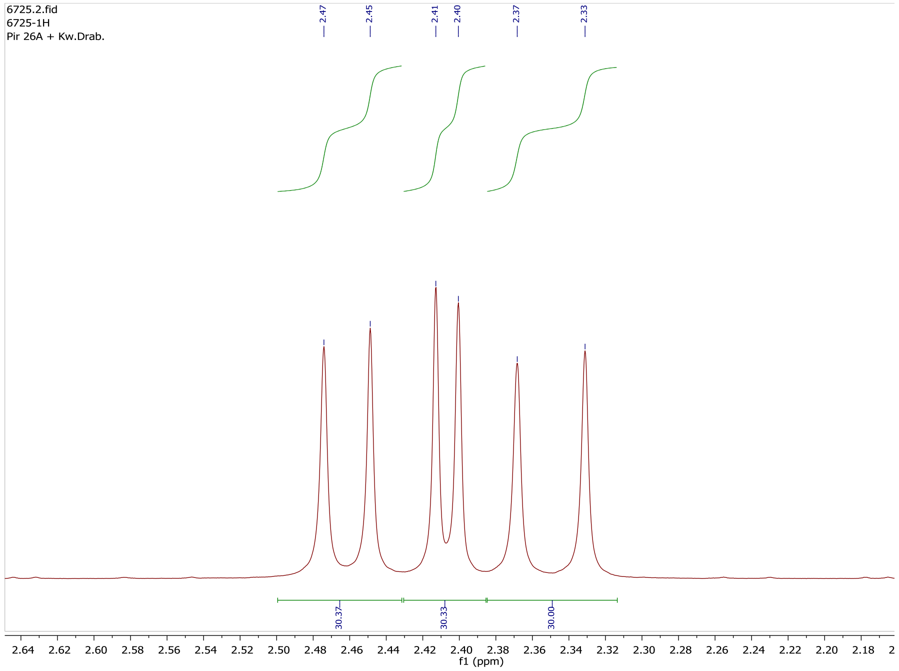
6725.2.fid
6725-1H
Pir 26A + Kw.Drab.
2.47
2.45
2.41
2.40
2.37
2.33
30.37
30.33
30.00
2.64 2.62 2.60 2.58 2.56 2.54 2.52 2.50 2.48 2.46 2.44 2.42 2.40 2.38 2.36 2.34 2.32 2.30 2.28 2.26 2.24 2.22 2.20 2.18
f1 (ppm)

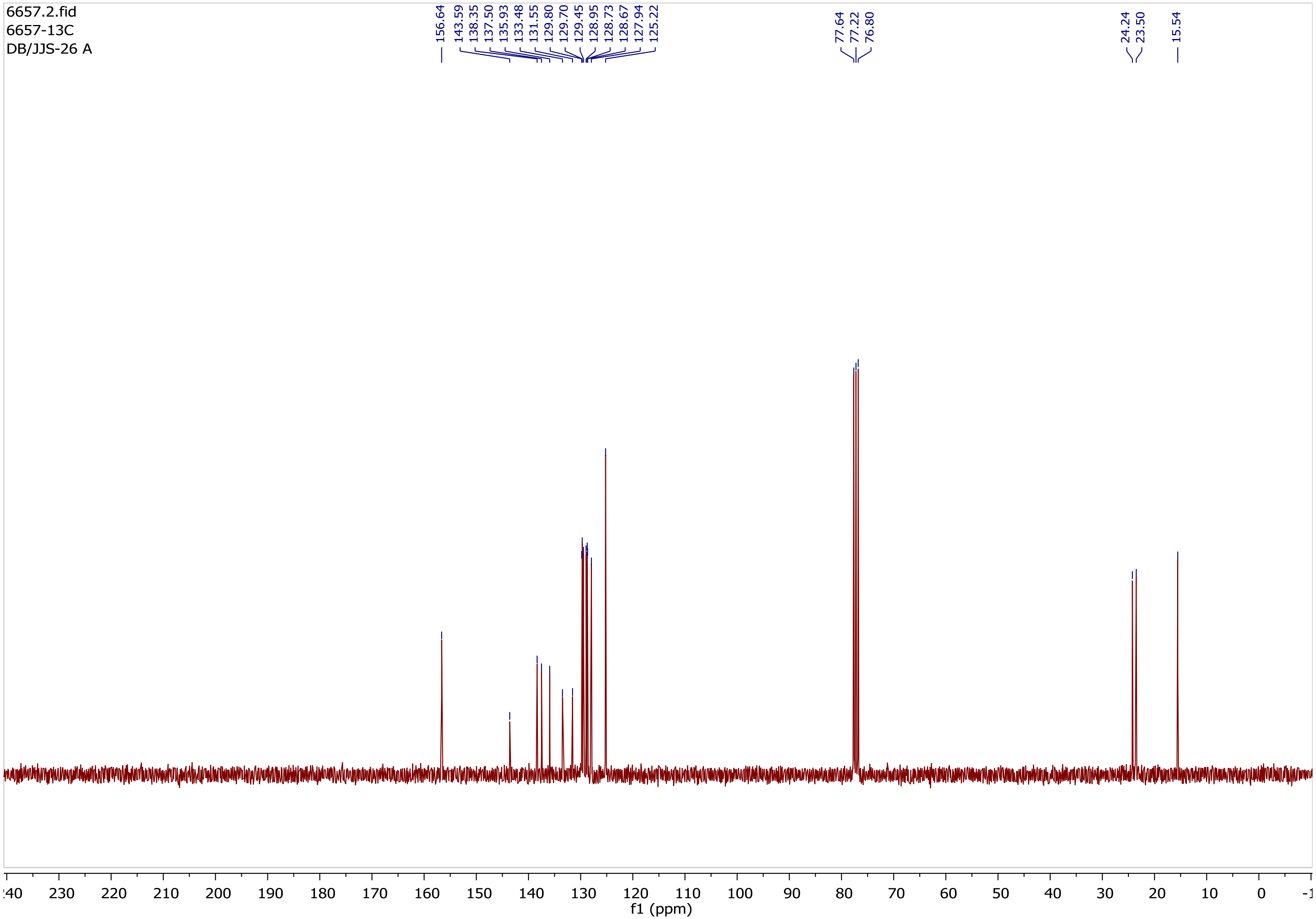

6657.2.fid
6657-13C
DB/JJS-26 A
156.64
143.59
138.35
137.50
135.93
133.48
131.55
129.80
129.70
129.45
128.95
128.73
128.67
127.94
125.22
77.64
77.22
76.80
24.24
23.50
15.54
240 230 220 210 200 190 180 170 160 150 140 130 120 110 100 90 80 70 60 50 40 30 20 10 0 -10
f1 (ppm)
